# A *MUC13* Variant Enhances Intestinal Ferric Iron Absorption in Unexplained Hyperferritinemia

**DOI:** 10.1111/liv.70901

**Published:** 2026-10-26

**Authors:** Jonas Callet, Yong H. Sheng, Yaowu He, Maël Silva Rodriguez, Céline Chéry, Ramia Safar, Françoise Barbé, Mouni Bensenane, Rosa‐Maria Guéant‐Rodriguez, Fares Namour, Guillaume Gauchotte, Janelle Louise Hancock, Hana Manceau, Thibaud Lefebvre, Dimitri Tchernitchko, Hervé Puy, Timothy H. Florin, Jakob Begun, John D. Hooper, Jean‐Pierre Bronowicki, Michael A. McGuckin, Jean‐Louis Guéant, Abderrahim Oussalah

**Affiliations:** ^1^ Department of Genomic Medicine, Division of Biochemistry Molecular Biology and Nutrition, University Hospital of Nancy Nancy France; ^2^ QIMR Berghofer Medical Research Institute, Cancer Program Herston Queensland Australia; ^3^ Cancer Biology Group, Mater Research Institute‐University of Queensland Woolloongabba Queensland Australia; ^4^ University of Lorraine, INSERM UMR_S 1256, Nutrition, Genetics, and Environmental Risk Exposure (NGERE), Faculty of Medicine of Nancy Nancy France; ^5^ Reference Center for Inborn Errors of Metabolism (ORPHA67872), University Hospital of Nancy Nancy France; ^6^ Department of Gastroenterology and Liver Diseases University Hospital of Nancy Nancy France; ^7^ Department of Pathology University Hospital of Nancy Nancy France; ^8^ Proteomics Core Facility, Translational Research Institute Woolloongabba Australia; ^9^ Centre de Recherches sur l'Inflammation, INSERM U1149, Université Paris Cité Paris France; ^10^ Laboratory of Biochemistry, Beaujon University Hospital, Assistance Publique‐Hôpitaux de Paris Clichy France; ^11^ Centre Français des Porphyries, Hôpital Louis Mourier, Assistance Publique‐Hôpitaux de Paris Colombes France; ^12^ Inflammatory Bowel Diseases Group, Mater Research Institute‐The University of Queensland, Translational Research Institute Woolloongabba Queensland Australia; ^13^ Faculty of Medicine, Dentistry and Health Sciences, the University of Melbourne Parkville Victoria Australia; ^14^ Joan Klein Jacobs Center for Precision Nutrition and Health, Cornell University Ithaca New York USA

**Keywords:** calreticulin, exome sequencing, ferritins, integrins, iron overload, mucins

## Abstract

**Background & Aims:**

Hyperferritinemia frequently remains unexplained despite comprehensive evaluation. We conducted a sequencing‐based exome association study to identify genetic variants associated with plasma ferritin levels in patients with unexplained hyperferritinemia.

**Methods:**

Gene‐based association testing using the combined multivariate and collapsing method with Bayesian inference was performed in patients referred for clinical exome sequencing (*n* = 392) after excluding known causes of hyperferritinemia. Findings were validated in an independent replication cohort (*n* = 263). Functional studies included *Muc13* knockout mice, intestinal cell models, proteomic analysis, and site‐directed mutagenesis.

**Results:**

The *MUC13* p.Arg503Ser variant showed extreme Bayesian evidence for association with increased plasma ferritin, iron, and transferrin saturation in the homozygous state. In the replication cohort, the homozygous 503Ser genotype was the strongest independent predictor of elevated transferrin saturation (odds ratio, 4.52; 95% CI, 1.55–13.19; *p* = 0.006). Conversely, *Muc13* knockout mice exhibited lower plasma iron and transferrin saturation, consistent with a role for MUC13 in intestinal iron absorption. In human intestinal cells, *MUC13* knockdown reduced ferric iron uptake, whereas *MUC13* overexpression increased it. Proteomic analysis identified integrin alpha 6 and integrin beta 4 as MUC13‐associated candidates. Cells expressing the homozygous 503Ser variant showed higher cell surface MUC13 expression and increased ferritin heavy chain 1, calreticulin, and nuclear factor erythroid 2‐related factor 2 levels after iron exposure.

**Conclusions:**

These findings support a role for MUC13 in intestinal ferric iron absorption and a causal link between the *MUC13* homozygous 503Ser genotype and enhanced cellular iron uptake, which could contribute to a better understanding of unexplained hyperferritinemia.

## Key Points

- The *MUC13* p.Arg503Ser variant was identified in a sequencing‐based exome association study of patients with unexplained hyperferritinemia as being strongly associated with plasma ferritin, iron, and transferrin saturation.
- The association was replicated in an independent cohort of 263 patients with unexplained hyperferritinemia and documented hepatic iron overload, in whom the homozygous 503Ser/Ser genotype was the strongest independent predictor of elevated transferrin saturation.
- Functional validation using knockout mice and human intestinal cell models demonstrated that MUC13 modulates ferric iron absorption, consistent with involvement of the mucin‐integrin‐calreticulin/mobilferrin pathway.
- Proteomic analysis identified integrin alpha 6 (ITGA6) and integrin beta 4 (ITGB4) as MUC13‐associated proteins, providing molecular evidence linking MUC13 to the integrin‐mediated ferric iron uptake pathway.
- Genotyping the *MUC13* p.Arg503Ser variant may refine the diagnostic assessment of unexplained hyperferritinemia.

**Lay Summary**

The *MUC13* p.Arg503Ser genetic variant was associated with elevated ferritin and iron levels in patients with unexplained hyperferritinemia. Using mouse models and human intestinal cells, we found that MUC13 promotes the absorption of a specific form of dietary iron (ferric iron) through a pathway involving integrins and calreticulin. These findings suggest that testing for this variant may help clarify why ferritin levels remain elevated when the standard work‐up is unremarkable.

**Abbreviations**
ACMG: American College of Medical Genetics and Genomics
ALAT: alanine aminotransferase
ANOVA: analysis of variance
ASAT: aspartate aminotransferase
BF[m]: Bayes factor
BMI: body mass index
BWA: Burrows‐Wheeler Aligner
CALR: calreticulin
cDNA: complementary DNA
CES: clinical exome sequencing
CI: confidence interval
CMC: combined multivariate and collapsing
Cr.I: credible interval
DCYTB: duodenal cytochrome b reductase 1
DMT1: divalent metal transporter 1
FTH1: ferritin heavy chain 1
GATK: Genome Analysis Toolkit
gnomAD: Genome Aggregation Database
HES: hematoxylin–eosin‐saffron
HGVS: Human Genome Variation Society
IPA: Ingenuity Pathway Analysis
IQR: interquartile range
ITGA6: integrin subunit alpha 6
ITGB4: integrin subunit beta 4
LC–MS/MS: liquid chromatography–tandem mass spectrometry
MFI: median fluorescence intensity
MRI: magnetic resonance imaging
MUC13: mucin 13
NRF2: nuclear factor erythroid 2‐related factor 2
OR: odds ratio
RR: relative risk
SEM: standard error of the mean
siRNA: small interfering RNA
TFRC: transferrin receptor
WT: wild type

## 1 Introduction

Hemochromatosis associated with iron overload represents a major inherited metabolic disorder that contributes to the development of cirrhosis and hepatocellular carcinoma. Iron metabolism disorders are assessed and diagnosed primarily through the quantification of plasma ferritin levels. Hyperferritinemia is commonly encountered in routine clinical practice and is generally associated with increased ferritin synthesis or release of ferritin from the cytosol of damaged cells [1]. From a pathophysiological standpoint, hyperferritinemia can be classified as reactive, typically observed in inflammatory conditions, or primary, which is associated with genetic disorders such as *HFE*‐related hemochromatosis. Identification of hyperferritinemia with iron overload in the setting of a genetic disorder is essential to prevent complications and end‐organ damage [2, 3, 4, 5, 6].

Despite comprehensive evaluation, hyperferritinemia often remains unexplained in clinical practice [1, 7], suggesting that unidentified genetic factors may contribute to this condition. Several genome‐wide association studies and meta‐analyses have reported multiple loci associated with biomarkers of iron homeostasis, including plasma ferritin [8, 9, 10, 11, 12, 13, 14, 15, 16]. The most recent of these studies identified up to 123 genetic loci associated with iron‐related biomarkers in cohorts comprising more than 250 000 individuals [8, 9]. However, these studies were conducted in general population cohorts without extensive clinical and laboratory phenotyping to exclude individuals with explainable reactive or primary hyperferritinemia. This approach may dilute genetic signals by including subjects whose hyperferritinemia has identifiable secondary causes. Furthermore, genome‐wide association studies primarily detect common variants captured by genotyping arrays, whereas rare coding variants with potentially larger effect sizes remain underexplored. To date, no sequencing‐based exome association study has investigated the contribution of coding genetic variations to plasma ferritin levels in a clinically characterized population with unexplained hyperferritinemia.

In the present study, we performed a sequencing‐based exome association study to identify genetic variants associated with plasma ferritin levels in patients in whom non‐genetic and known genetic causes of hyperferritinemia were excluded through stringent clinical and biological criteria. We identified a *MUC13* missense variant (p.Arg503Ser) associated with hyperferritinemia and increased transferrin saturation and validated this association in an independent replication cohort. To establish the functional relevance of this finding, we characterized the role of MUC13 in iron metabolism using cell and animal models.

## 2 Patients and Methods

### 2.1 Study Design, Setting, and Patient Selection Criteria

We performed a retrospective observational study to identify genetic variants associated with hyperferritinemia unexplained by known genetic or acquired causes. The study population (discovery cohort) was derived from consecutive adult patients referred for clinical exome sequencing (CES) at the Department of Genomic Medicine, University Hospital of Nancy (Nancy, France), to investigate suspected Mendelian phenotypes [17, 18]. The inclusion criteria were: (i) CES prescribed for any indication between January 2017 and June 2020; and (ii) availability of biochemical results for at least one iron metabolism parameter. To ensure that only patients with unexplained hyperferritinemia were retained, we applied the following exclusion criteria targeting known genetic and acquired causes of elevated ferritin: (i) previous genetic diagnosis related to iron overload; (ii) hepatic cytolysis (aspartate aminotransferase [ASAT] > 200 U/L and/or alanine aminotransferase [ALAT] > 200 U/L); (iii) biological inflammatory syndrome (C‐reactive protein > 10 mg/L); (iv) history of a chronic inflammatory disease; (v) history of chronic kidney disease; (vi) history of blood transfusion; (vii) history of oral or intravenous iron or erythropoietin therapy; (viii) history of blood removal to treat iron overload; (ix) no data available on plasma ferritin level; and (x) age < 18 years. Given the observational design, all clinical and biochemical investigations, notably those related to iron metabolism assessment, were performed at the discretion of treating physicians. Clinical and biochemical data were collected by electronic chart review, including demographics, iron metabolism parameters (ferritin, iron, transferrin, transferrin saturation), liver function tests, and iron‐related treatment history. Ethical approval for this study (approval no. 2020/264) was provided by the Ethics Committee of the University Hospital of Nancy. The Nancy Biochemical Database is registered with the French National Commission on Informatics and Liberty (CNIL N°1763197v0). Written informed consent was obtained from all participants. The study was conducted and reported in accordance with the STROBE guidelines for observational studies. Further details on the French framework for genetic diagnosis, data collection, and data sharing are provided in the Supplementary Appendix.

### 2.2 Next‐Generation Sequencing and Variant Analysis

CES was performed using the TruSight One panel (Illumina MiSeq) or the TruSight One Expanded panel (Illumina NextSeq 550), covering 4733 common disease‐associated genes (Table S1). Sequence alignment to the GRCh37/hg19 reference genome was performed using the Burrows‐Wheeler Aligner (BWA‐MEM algorithm), with variant calling using GATK HaplotypeCaller. Ancestry inference was performed using principal component analysis with 1000 Genomes Project reference populations. Variants were filtered based on quality metrics (read depth > 20, genotype quality score of 99) and confirmed by visual inspection of BAM files. Functional annotation was performed using MutationTaster, FATHMM, MetaSVM/MetaLR, SIFT, PROVEAN, and DANN, with pathogenicity classification according to ACMG criteria (see Supplementary Appendix for details on quality controls and variant filtering). Phosphorylation sites were predicted for the reference and the variant *MUC13* sequences using NetPhos 3.1 and GPS 6.0 (see Supplementary Appendix for details).

### 2.3 *Muc13* Knockout Mouse Model

*Muc13* ^−/−^ mice in the C57BL/6 background and their wild‐type littermates (*Muc13* ^+/+^) were bred at the Mater Research Institute, The University of Queensland. All experiments were approved by the University of Queensland Animal Experimentation Ethics Committee. Mice were sex‐ and age‐matched (12–13 weeks; *Muc13* ^−/−^, *n* = 6; *Muc13* ^+/+^, *n* = 7). Plasma iron, transferrin, and ferritin levels were measured using Atellica and Beckman Coulter analyzers. Plasma hepcidin was quantified by LC–MS/MS. Liver histopathology was assessed using HES, Sirius red, and Perls Prussian blue staining. Duodenal epithelial cells were isolated from *Muc13* ^−/−^ and *Muc13* ^+/+^ mice for protein expression analysis (see Supplementary Appendix for detailed protocols).

### 2.4 Cell Culture and Functional Assays

Human LS513 intestinal epithelial cells (high endogenous *MUC13* expression) and SW480 cells (low *MUC13* expression) were used for complementary loss‐of‐function and gain‐of‐function experiments. *MUC13* silencing was performed in LS513 cells using SMARTpool siRNA (Dharmacon), while stable *MUC13* overexpression was achieved in SW480 cells using retroviral transduction with FLAG‐tagged *MUC13* cDNA. Cells were treated with iron sucrose (Fe^3+^; 0–320 μg/mL) to assess iron metabolism responses. Western blotting was performed for ferritin heavy chain (FTH1), calreticulin (CALR), NRF2, and MUC13. Co‐immunoprecipitation experiments assessed physical interactions between MUC13 and calreticulin. Iron concentrations in cell culture supernatants were measured to evaluate cellular iron uptake, and real‐time cell proliferation was monitored using the Incucyte S3 Live‐Cell Analysis System to assess iron‐induced cytotoxicity (see Supplementary Appendix for detailed protocols).

### 2.5 Proteomic Analysis

MUC13‐associated proteins were identified by immunoprecipitation followed by LC–MS/MS on an Orbitrap Fusion mass spectrometer. Data were processed using MaxQuant software and analyzed using Ingenuity Pathway Analysis (IPA) to identify enriched pathways and biological functions (see Supplementary Appendix for details).

### 2.6 Generation and Characterization of the *MUC13* p.Arg503Ser Variant

The *MUC13* p.Arg503Ser variant was generated by site‐directed mutagenesis using Pfu Ultra polymerase (Agilent) and confirmed by DNA sequencing (Table S2). SW480 cells stably expressing wild‐type *MUC13* (503Arg/Arg) or mutant *MUC13* (503Ser/Ser) were generated by retroviral transduction. Cell surface and total cellular MUC13 expression were quantified by flow cytometry using an anti‐FLAG antibody on a CytoFLEX flow cytometer (see Supplementary Appendix for details).

### 2.7 Statistical Analysis

Gene‐based association testing was performed using the combined multivariate and collapsing (CMC) method with linear regression. Rare variants (minor allele frequency < 1% in gnomAD) were collapsed within each gene to increase statistical power for detecting gene‐level associations, as individual rare variants typically lack sufficient carrier frequency for reliable per‐variant testing. For subsequent per‐variant analysis, only variants with minor allele frequency greater than 1% were evaluated to ensure adequate genotype frequencies for robust effect estimation and Bayesian inference. Covariates included age, sex, ASAT, ALAT, C‐reactive protein, history of chronic liver disease, and the top five principal components from ancestry inference. Bonferroni correction was applied for multiple testing. Bayesian analysis of variance (ANOVA) was used to validate associations, with the Bayes factor (BF[m]) interpreted according to the Jeffreys scale: 3–10 (moderate), 10–30 (strong), 30–100 (very strong), and > 100 (extremely strong evidence). For animal and cellular experiments, comparisons were performed using the Student *t*‐test, the Mann–Whitney *U* test, or two‐way ANOVA with appropriate post hoc tests, as specified in figure legends. Analyses were performed using SNP and Variation Suite v8.9.0 (Golden Helix), MedCalc v20.014, JASP v0.14.1, and GraphPad Prism v8.0.0. Further details on the rationale for the statistical approach, handling of repeated measures, and Bayesian inference methodology are provided in the Supplementary Appendix.

## 3 Results

### 3.1 Patient Selection and Dataset Overview

Of 392 patients meeting inclusion criteria (1306 encounters), 483 encounters were retained after excluding 823 encounters meeting at least one exclusion criterion (Figure S1). The biochemical characteristics of the population are shown in Table S3. Median plasma ferritin was 120 μg/L (IQR, 43 to 270) and transferrin saturation was 26% (IQR, 19 to 34). Transaminase and C‐reactive protein levels were within ranges consistent with the absence of hepatic cytolysis or inflammatory syndrome. The genomic dataset contained 316 811 variants, of which 265 339 were covered by the sequencing panels. After excluding 71 029 synonymous variants, 91 301 of the remaining 194 310 variants had good quality metrics and 45 575 were non‐monomorphic and analyzed.

### 3.2 Exome Association Study Identifies Genes Associated With Plasma Ferritin Levels

Gene‐based testing using CMC regression analysis with covariate adjustment (age, sex, ASAT, ALAT, C‐reactive protein, history of chronic liver disease, and the top 5 principal components from the ancestry inference analysis [Figure S2]) identified 107 genes significantly associated with plasma ferritin levels after Bonferroni correction (Table S4). Among the 193 variants with minor allele frequency greater than 1% within these 107 genes, 31 were significantly associated with plasma ferritin levels after Bonferroni correction in per‐variant linear regression (Table 1). Rare variants (minor allele frequency < 1%) were evaluated collectively at the gene level during burden testing but were not assessed individually owing to insufficient carrier frequencies for reliable effect estimation.

**TABLE 1:** Linear regression reporting the 31 Bonferroni‐significant variants associated with plasma ferritin levels.

Variant	Chr	Position*	Ref/Alt	Gene	Sequence Ontology	HGVS c. HGVS p.	FvR Model Value *P*	Bonf. *p*‐value	Beta	Beta SE
2:190925077‐SNV	2	190 925 077	T/C	*MSTN*	missense_variant	NM_005259.3:c.458A>G NP_005250.1:p.Lys153Arg	6.71E‐12	1.30E‐09	312.9	43.4
13:28239999‐SNV	13	28 239 999	A/G	*POLR1D*	missense_variant	NM_152705.3:c.278A>G NP_689918.1:p.His93Arg	2.68E‐10	5.17E‐08	513.2	78.0
19:44605324‐SNV	19	44 605 324	A/G	*ZNF224*	missense_variant	NM_013398.5:c.181A>G NP_037530.2:p.Arg61Gly	4.60E‐10	8.88E‐08	265.4	40.9
3:43743914‐SNV	3	43 743 914	G/T	*ABHD5*	missense_variant	NM_016006.6:c.341G>T NP_057090.2:p.Arg114Leu	5.63E‐10	1.09E‐07	270.6	41.9
1:91404256‐SNV	1	91 404 256	C/A	*ZNF644*	missense_variant	NM_201269.3:c.2655G>T NP_958357.1:p.Glu885Asp	3.61E‐09	6.97E‐07	188.1	30.7
1:78392446‐SNV	1	78 392 446	G/A	*NEXN*	missense_variant	NM_144573.4:c.733G>A NP_653174.3:p.Gly245Arg	7.07E‐09	1.36E‐06	116.0	19.3
6:116446576‐SNV	6	116 446 576	A/G	*COL10A1*	missense_variant	NM_000493.4:c.80T>C NP_000484.2:p.Met27Thr	3.40E‐08	6.56E‐06	−89.1	15.6
2:109367977‐SNV	2	109 367 977	A/G	*RANBP2*	splice_region_variant	NM_006267.5:c.1456‐7A>G NA	4.85E‐08	9.36E‐06	−124.4	22.1
19:49671507‐SNV	19	49 671 507	G/A	*TRPM4*	intron_variant	NM_017636.4:c.449‐10G>A NA	7.82E‐08	1.51E‐05	300.5	54.3
14:35871217‐SNV	14	35 871 217	G/A	*NFKBIA*	3_prime_UTR_variant	NM_020529.3:c.*2C>T NA	2.96E‐07	5.71E‐05	−82.1	15.6
X:100662901‐SNV	X	100 662 901	G/A	*GLA*	5_prime_UTR_variant	NM_000169.3:c.‐10C>T NA	9.31E‐07	1.80E‐04	173.7	34.5
1:197150257‐Del	1	197 150 257	A/−	*ZBTB41*	intron_variant	NM_194314.3:c.1547‐10delT NA	1.11E‐06	2.14E‐04	241.6	48.4
1:35250457‐SNV	1	35 250 457	C/T	*GJB3*	missense_variant	NM_024009.3:c.94C>T NP_076872.1:p.Arg32Trp	1.13E‐06	2.18E‐04	270.7	54.2
X:41383298‐Ins	X	41 383 298	−/A	*CASK*	intron_variant	NM_001367721.1:c.2521‐11dupT NA	1.90E‐06	3.67E‐04	108.6	22.3
17:28548810‐SNV	17	28 548 810	C/G	*SLC6A4*	missense_variant	NM_001045.6:c.167G>C NP_001036.1:p.Gly56Ala	1.92E‐06	3.71E‐04	251.7	51.6
6:161006105‐SNV	6	161 006 105	C/T	*LPA*	missense_variant	NM_005577.4:c.4262G>A NP_005568.2:p.Arg1421Gln	2.03E‐06	3.92E‐04	257.8	53.0
12:56848079‐SNV	12	56 848 079	C/T	*MIP*	missense_variant	NM_012064.4:c.319G>A NP_036196.1:p.Val107Ile	2.09E‐06	4.03E‐04	295.2	60.8
2:120005312‐SNV	2	120 005 312	G/A	*STEAP3*	missense_variant	NM_182915.3:c.580G>A NP_878919.2:p.Ala194Thr	1.74E‐05	3.36E‐03	−172.0	39.3
5:149212430‐SNV	5	149 212 430	G/A	*PPARGC1B*	missense_variant	NM_133263.4:c.794G>A NP_573570.3:p.Arg265Gln	1.84E‐05	3.55E‐03	235.5	53.9
3:186443018‐SNV	3	186 443 018	T/C	*KNG1*	missense_variant	NM_001102416.3:c.533T>C NP_001095886.1:p.Met178Thr	2.62E‐05	5.06E‐03	−56.0	13.1
3:176765269‐SNV	3	176 765 269	T/C	*TBL1XR1*	splice_region_variant	NM_024665.7:c.766+5A>G NA	4.82E‐05	9.30E‐03	81.2	19.6
16:3293880‐SNV	16	3 293 880	A/G	*MEFV*	missense_variant	NM_000243.3:c.1772T>C NP_000234.1:p.Ile591Thr	6.74E‐05	1.30E‐02	−145.5	35.9
16:3294246‐SNV	16	3 294 246	G/A	*MEFV*	splice_region_variant	NM_000243.3:c.1759+8C>T NA	7.54E‐05	1.46E‐02	−450.9	112.0
3:46399208‐SNV	3	46 399 208	G/A	*CCR2*	missense_variant	NM_001123396.4:c.190G>A NP_001116868.1:p.Val64Ile	9.43E‐05	1.82E‐02	−107.4	27.1
17:57141769‐Del	17	57 141 769	A/−	*TRIM37*	splice_region_variant	NM_015294.6:c.810‐3delT NA	1.00E‐04	1.93E‐02	106.5	26.9
3:124627024‐SNV	3	124 627 024	T/G	*MUC13*	missense_variant	NM_033049.4:c.1509A>C† NP_149038.3:p.Arg503Ser†	1.17E‐04	2.26E‐02	79.2	20.2
17:80789468‐SNV	17	80 789 468	G/A	*ZNF750*	missense_variant	NM_024702.3:c.863C>T NP_078978.2:p.Pro288Leu	1.41E‐04	2.72E‐02	−109.7	28.4
17:1673276‐SNV	17	1 673 276	C/T	*SERPINF1*	missense_variant	NM_002615.7:c.215C>T NP_002606.3:p.Thr72Met	1.68E‐04	3.24E‐02	−58.6	15.3
1:197168625‐SNV	1	197 168 625	C/A	*ZBTB41*	missense_variant	NM_194314.3:c.979G>T NP_919290.2:p.Asp327Tyr	1.74E‐04	3.36E‐02	337.8	88.6
6:116441646‐SNV	6	116 441 646	C/G	*COL10A1*	missense_variant	NM_000493.4:c.1633G>C NP_000484.2:p.Gly545Arg	1.84E‐04	3.55E‐02	−95.9	25.3
2:202122995‐SNV	2	202 122 995	A/G	*CASP8*	missense_variant	NM_001080125.2:c.41A>G NP_001073594.1:p.Lys14Arg	1.92E‐04	3.71E‐02	−56.3	14.9

Abbreviations: Alt, alternative allele; Bonf, Bonferroni; Chr, chromosome; FvR, full versus reduced; HGVS, Human Genome Variation Society; Ref, reference allele; SE, standard error.

* Genomic positions are reported according to GRCh37.

^†^ Variant nomenclature according to RefSeq (NM_033049.4:c.1509A>C; NP_149038.3:p.Arg503Ser) and Ensembl (ENST00000311075.3:c.1506A>C, p.Arg502Ser) databases.

### 3.3 Bayesian Analyses Identify a Strong Association Between the *MUC13* p.Arg503Ser Variant and Iron Status Parameters

Bayesian ANOVA validated 11 of the 31 Bonferroni‐significant variants, with four showing extreme evidence (BF[m] greater than 100): *MUC13*, *ZNF644*, *TBL1XR1*, and *TRIM37* (Figure S3A–D and Table 2). The *MUC13* missense variant (RefSeq NM_033049.4:c.1509A>C; NP_149038.3:p.Arg503Ser; Ensembl ENST00000311075.3:c.1506A>C, p.Arg502Ser) demonstrated the strongest association with plasma ferritin levels: BF(m) = 5.26 × 10^19^; mean Arg/Arg, 185 μg/L (95% Cr.I, 156–213); mean Arg/Ser, 203 μg/L (95% Cr.I, 166–240); mean Ser/Ser, 782 μg/L (95% Cr.I, 513–1051) (Table 2 and Figure 1). Among the 11 cross‐validated variants, only *MUC13* p.Arg503Ser showed Bayesian evidence of association with iron status parameters, reaching extreme evidence for both plasma iron and transferrin saturation (Figure 1, Tables S5 and S6, and Figures S4 and S5). None of the 11 variants showed Bayesian evidence of an association with plasma transferrin concentration (Table S7 and Figure S6). None of the 11 variants showed Bayesian evidence of an association with body mass index (Table S8), alanine aminotransferase level (Table S9), or C‐reactive protein level (Table S10), indicating that ferritin associations were not confounded by metabolic syndrome, hepatic cytolysis, or inflammation.

**TABLE 2:** Bayesian analysis of variance reporting the 11 cross‐validated genetic variants associated with plasma ferritin levels among the 31 Bonferroni‐significant variants from the genotype association study using linear regression.

Variant	Chr	Position	Ref/Alt	dbSNP ID	Gene	AAF	ACMG	BF(m)	Ref‐Ref (AA)		Ref‐Alt (AB)		Alt‐Alt (BB)	
									N	Mean, Ferritin (μg/L) (95% Cr. I)	N	Mean, Ferritin (μg/L) (95% Cr. I)	N	Mean, Ferritin (μg/L) (95% Cr. I)
3:124627024‐SNV	3	124 627 024	T/G	rs1127233	*MUC13*	0.239	B	5.26 × 10^19^	276	185 (156–213)	169	203 (166–240)	38	782 (513–1051)
1:91404256‐SNV	1	91 404 256	C/A	rs41286761	*ZNF644*	0.022	B	75 370	410	202 (169–235)	73	440 (345–535)	—	—
3:176765269‐SNV	3	176 765 269	T/C	rs3749234	*TBL1XR1*	0.211	B	48 180	307	182 (157–207)	155	305 (248–363)	21	566 (70–1061)
17:57141769‐Del	17	57 141 769	A/−	rs367700401	*TRIM37*	0.254	VUS	137	346	199 (163–235)	137	338 (271–404)	—	—
1:78392446‐SNV	1	78 392 446	G/A	rs1166698	*NEXN*	0.182	B	51	307	197 (168–227)	159	289 (213–365)	17	498 (284–713)
3:43743914‐SNV	3	43 743 914	G/T	rs148743497	*ABHD5*	7.77 × 10^−3^	B	23	456	225 (192–258)	27	455 (310–601)	—	—
19:44605324‐SNV	19	44 605 324	A/G	rs118149610	*ZNF224*	0.014	B	10	460	227 (195–260)	23	451 (273–629)	—	—
17:28548810‐SNV	17	28 548 810	C/G	rs6355	*SLC6A4*	0.012	B	5	466	230 (197–262)	17	462 (255–668)	—	—
3:186443018‐SNV	3	186 443 018	T/C	rs1656922	*KNG1*	0.537	B	4	164	292 (238–346)	180	252 (184–319)	139	156 (130–184)
12:56848079‐SNV	12	56 848 079	C/T	rs74641138	*MIP*	0.024	B	4	465	230 (197–262)	18	451 (225–677)	—	—
6:161006105‐SNV	6	161 006 105	C/T	rs41272112	*LPA*	9.53 × 10^−3^	B	3	458	229 (196–262)	25	412 (259–564)	—	—

*Note:* Genomic positions are reported according to GRCh37.

Abbreviations: *ABHD5*, Abhydrolase Domain Containing 5, Lysophosphatidic Acid Acyltransferase; ACMG, American College of Medical Genetics and Genomics; Alt, alternative allele; B, benign; BF(m), Bayes factor; Chr, chromosome; Cr. I, credible interval; *KNG1*, Kininogen 1; *LPA*, Lipoprotein(a); *MIP*, Major Intrinsic Protein of Lens Fiber; *MUC13*, Mucin 13, Cell Surface Associated; N, number of observations; *NEXN*, Nexilin F‐Actin Binding Protein; Ref, reference allele; *SLC6A4*, Solute Carrier Family 6 Member 4; *TBL1XR1*, Transducin Beta Like 1 X‐Linked Receptor 1; *TRIM37*, Tripartite Motif Containing 37; VUS, variant of unknown significance; *ZNF224*, Zinc Finger Protein 224; *ZNF644*, Zinc Finger Protein 644.

**FIGURE 1:**
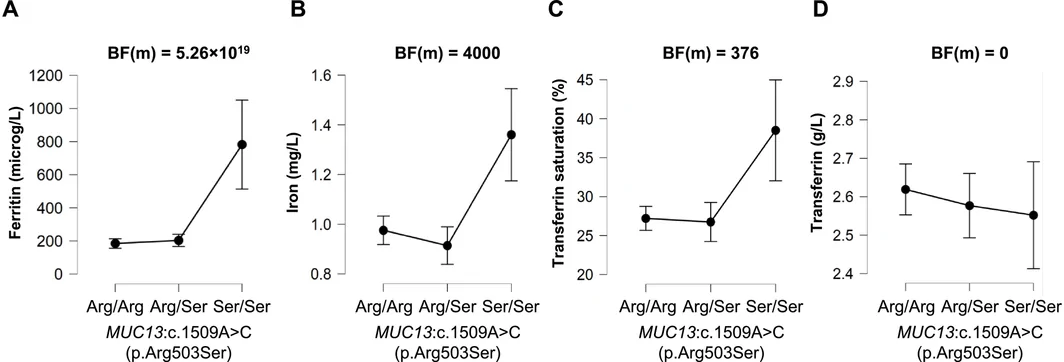
Bayesian analyses identify a strong association between the *MUC13* p.Arg503Ser variant and iron status parameters. Bayesian analysis of variance showing the association between the *MUC13* p.Arg503Ser variant and plasma levels of ferritin (μg/L), iron (mg/L), transferrin saturation (%), and transferrin (g/L). The Bayes factor (BF[m]) is shown at the top of each panel. The mean and 95% credible interval (95% Cr.I) are reported for each genotype subgroup (Arg/Arg, Arg/Ser, and Ser/Ser).

Given the strong genetic association and the known high expression of MUC13, a transmembrane mucin glycoprotein, in the intestine [19], we investigated its potential role in iron absorption via the mucin‐integrin‐calreticulin/mobilferrin pathway [20].

### 3.4 Independent Replication Confirms Enrichment of the *MUC13* p.Arg503Ser Ser/Ser Genotype in Patients With Unexplained Hyperferritinemia and Elevated Transferrin Saturation

The replication cohort comprised 263 patients with unexplained hyperferritinemia recruited from three university hospitals in the Ile‐de‐France region between 2014 and 2023 (Table S11). All patients were of self‐reported European ancestry. All patients had undergone comprehensive genetic testing that excluded pathogenic variants in *HFE* (C282Y homozygous, compound C282Y/H63D heterozygous), *HJV*, *TFR2*, *HAMP*, *SLC40A1*, and *BMP6* genes. Secondary causes of hyperferritinemia were systematically excluded, including chronic or acute inflammatory syndrome, history of transfusions, hematological diseases, history of phlebotomy and iron supplementation, cellular lysis, dysmetabolic syndrome, and excessive alcohol consumption. The cohort was predominantly male (77.2%; 95% CI, 72.1–82.3) with a median age of 66 years (IQR, 55 to 74). Median serum ferritin was 935 μg/L (IQR, 612 to 1350), and median transferrin saturation was 55% (IQR, 39 to 74). Among patients with available transferrin saturation data (*n* = 222), 68.0% (95% CI, 61.8–74.2) had values exceeding 45%, and 47.3% (95% CI, 40.7–53.9) had values exceeding 55%. MRI‐based liver iron quantification was available in 98 patients and demonstrated a median hepatic iron concentration of 120 μmol/g (IQR, 70 to 190); 96.9% (95% CI, 93.5–100) exceeded the normal threshold of 36 μmol/g, and 33.7% (95% CI, 24.1–43.2) exhibited severe iron overload (> 160 μmol/g).

Genotyping for *MUC13* p.Arg503Ser revealed a Ser allele frequency of 28.7%, with genotype frequencies (Arg/Arg, 53.2%; Arg/Ser, 36.1%; Ser/Ser, 10.6%) consistent with Hardy–Weinberg equilibrium (*p* = 0.07). Comparison with gnomAD reference populations demonstrated that the frequency of the Ser/Ser genotype in the replication cohort (10.6%) was significantly higher than in the European non‐Finnish reference population from gnomAD (6.5%; RR, 1.63; 95% CI, 1.08–2.46; *p* = 0.018).

The distribution of genotypes differed according to transferrin saturation status. Among patients with transferrin saturation greater than 55%, the proportion of Ser/Ser carriers was 15.2% (95% CI, 8.3–22.2), compared with 4.3% (95% CI, 0.6–8.0) among those with transferrin saturation of 55% or less (Table S12). Multivariable logistic regression adjusting for sex, age, and serum ferritin concentration identified the *MUC13* 503Ser/Ser genotype as the strongest independent predictor of elevated transferrin saturation (> 55%), with an odds ratio of 4.52 (95% CI, 1.55–13.19; *p* = 0.006), exceeding the effect size of all other covariates in the model (Table S13).

### 3.5 *Muc13* ^−/−^ Mice Exhibit Impaired Iron Homeostasis

Plasma iron levels were significantly lower in *Muc13* ^−/−^ mice (17.2 μmol/L; IQR, 15.8 to 20.0) than in *Muc13* ^+/+^ mice (22.2 μmol/L; IQR, 21.6 to 25.6; *p* = 0.03). Transferrin saturation was similarly reduced (*Muc13* ^−/−^: 15.9% [IQR, 14.9 to 17.8] vs. *Muc13* ^+/+^: 22.1% [IQR, 19.8 to 22.4]; *p* = 0.02) (Figures 2A,B and Table S14). Plasma transferrin and ferritin levels did not differ between groups (Figures 2C,D and Table S14). Plasma hepcidin levels showed no significant difference between *Muc13* ^−/−^ and *Muc13* ^+/+^ mice, but substantial variation was observed in *Muc13* ^−/−^ mice (Figure 2E). Half of the *Muc13* ^−/−^ mice (3/6) had plasma hepcidin 4.5 ng/mL or lower compared with 0% (0/7) of *Muc13* ^+/+^ mice (*p* = 0.07) (Figures 2E,F and Table S14). *Muc13* expression in intestinal cells from *Muc13* ^−/−^ and *Muc13* ^+/+^ mice confirmed reduced *Muc13* expression in *Muc13* ^−/−^ mice (Figures S7 and S8A–D). Hepatic iron and Sirius red staining did not differ between *Muc13* ^−/−^ and *Muc13* ^+/+^ liver sections (Figure S8E–H), and iron concentration was similar in liver tissues from both groups (Table S14). These findings suggest that MUC13 modulates iron absorption specifically at the intestinal level.

**FIGURE 2:**
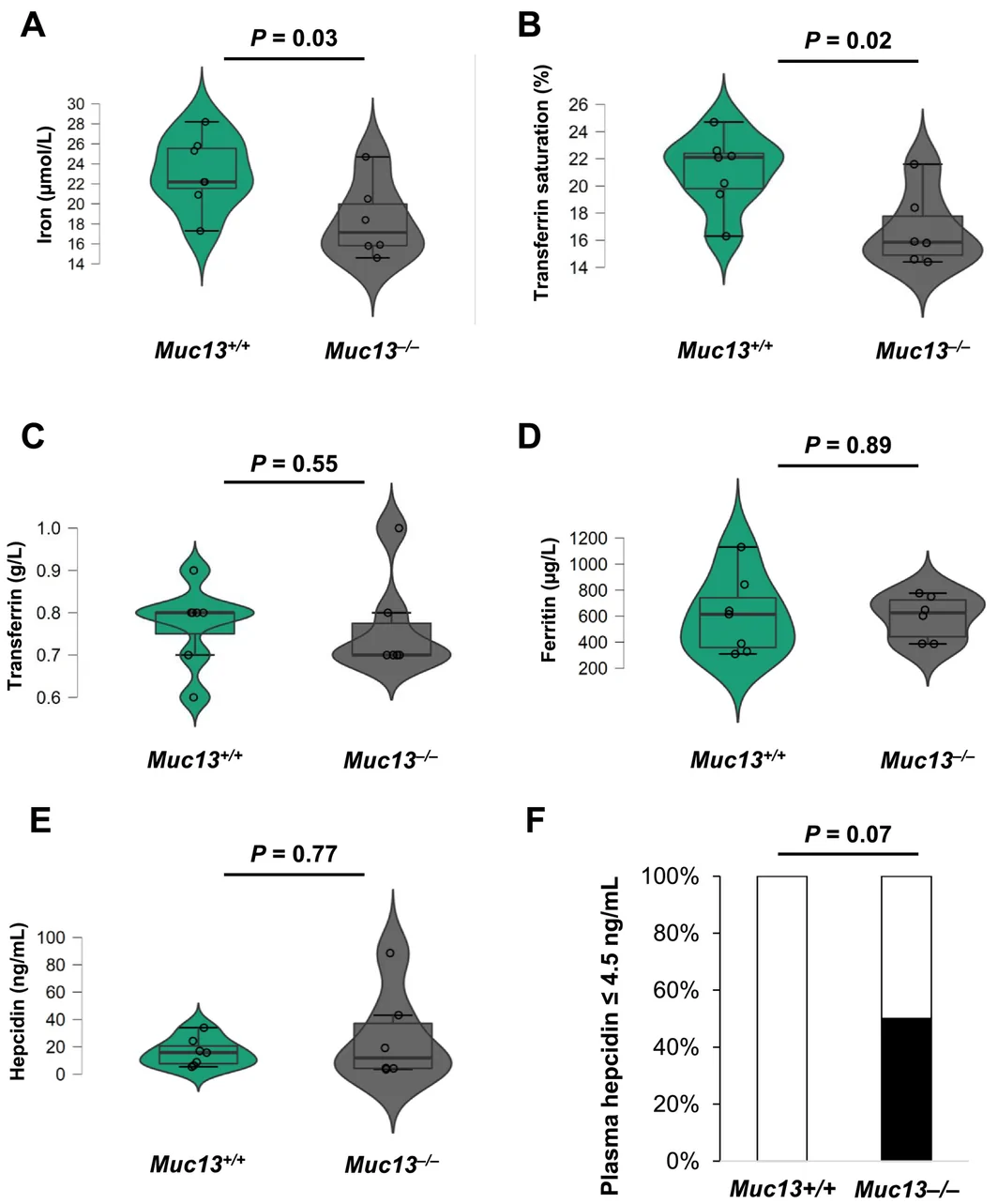
Iron metabolism parameters in *Muc13* ^*−/−*^ and *Muc13* ^*+/+*^ mice. Data are presented as violin plots with box plots indicating the interquartile range (*Muc13* ^*+/+*^, *n* = 7; *Muc13* ^*−/−*^, *n* = 6). (A) Plasma iron. (B) Transferrin saturation. (C) Plasma transferrin. (D) Plasma ferritin. (E) Plasma hepcidin. (F) Proportion of mice with plasma hepcidin ≤ 4.5 ng/mL (black column). Comparisons were performed using the Mann–Whitney U test (A‐E) and Fisher exact test (F).

### 3.6 MUC13 Regulates Iron Metabolism Proteins FTH1, CALR, and NRF2

In duodenal epithelial cells from *Muc13* ^−/−^ mice, levels of ferritin heavy chain 1 (*Fth1*), calreticulin/mobilferrin (*Calr*), and nuclear factor erythroid 2‐related factor 2 (*Nrf2*) were significantly lower than in *Muc13* ^+/+^ mice (Figure 3A). Consistent findings were observed in human intestinal cell models. In LS513 cells (high endogenous MUC13), siRNA‐mediated *MUC13* knockdown (greater than 90% reduction) decreased FTH1, CALR/mobilferrin, and NRF2 levels (Figure 3B). Conversely, *MUC13* overexpression in SW480 cells (low endogenous MUC13) significantly increased these proteins (Figure 3C).

**FIGURE 3:**
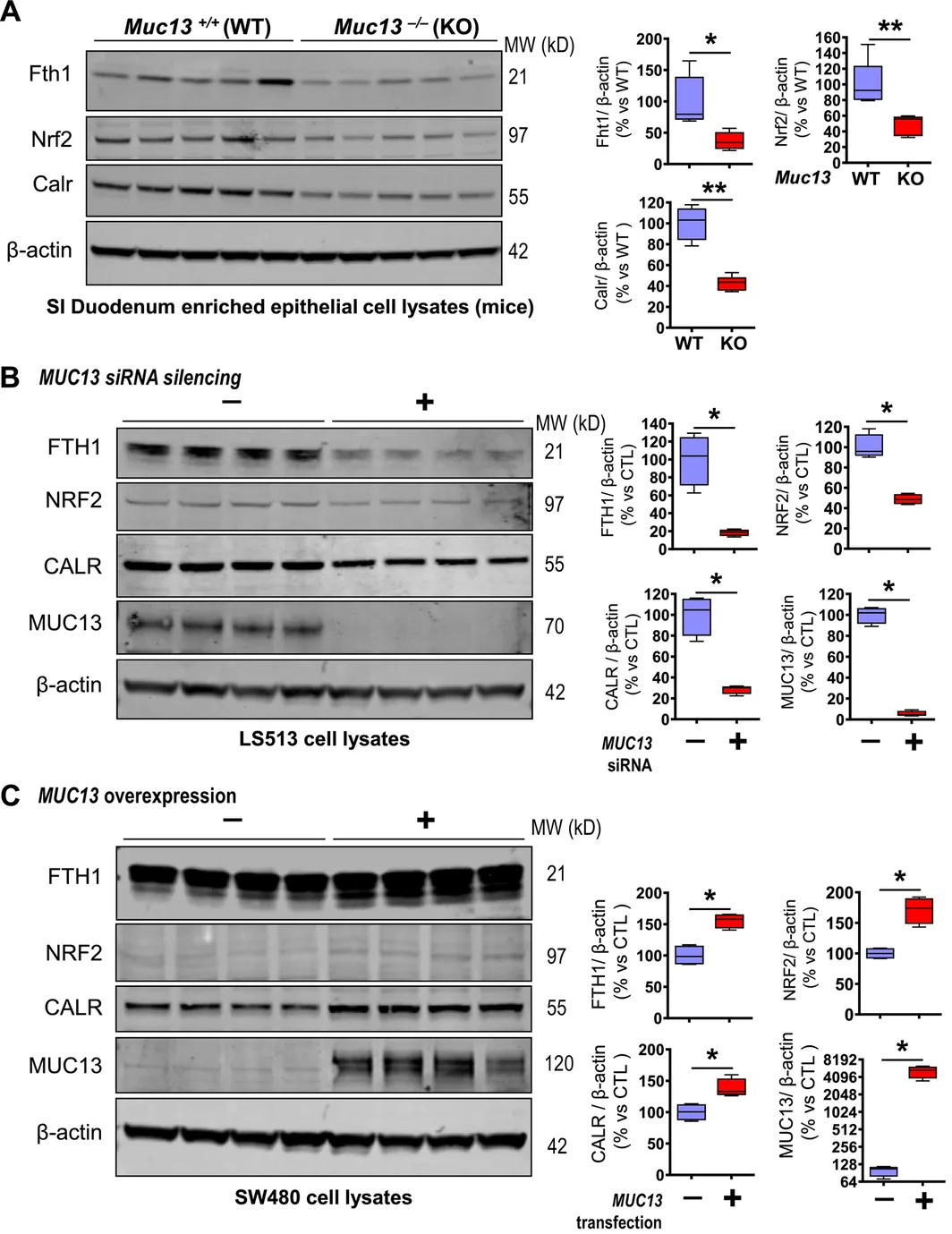
MUC13 upregulates the FTH1, CALR, and NRF2 proteins involved in iron metabolism. (A) Western blot analysis of small intestinal duodenal epithelial cells from *Muc13* ^−/−^ and *Muc13* ^+/+^ mice for ferritin heavy chain 1 (FTH1), calreticulin/mobilferrin (CALR/mobilferrin), and nuclear factor erythroid 2‐related factor 2 (NRF2) (*n* = 5). Densitometric analysis of FTH1, CALR/mobilferrin, and NRF2 levels normalized to β‐actin and expressed as a percentage of the level in WT is shown on the left. (B) LS513 cells were transfected with *MUC13* or control siRNA for 48 h (*n* = 4). FTH1, NRF2, CALR/mobilferrin, and MUC13 levels were then examined by Western blot analysis of LS513 cell lysates. Densitometric analysis of protein levels normalized to β‐Actin and expressed as a percentage of the level measured in control siRNA‐treated cells (shown on the left). (C) Control and MUC13‐overexpressing SW480 cells were harvested (*n* = 4). FTH1, NRF2, CALR/mobilferrin, and MUC13 levels were then examined by Western blot analysis of the SW480 cell lysates. Densitometric analysis of protein levels normalized to β‐Actin and expressed as a percentage of the level measured in control vector cells (shown on the left). The results are shown as box and whisker plots showing the median, quartiles, and range; the Mann–Whitney *U* test, * *p* < 0.05, **p < 0.01.

### 3.7 MUC13 Interacts With Integrin Family Proteins ITGA6 and ITGB4

Since Fe^3+^ is taken up by CALR/mobilferrin in the mucin‐integrin‐CALR/mobilferrin pathway, we performed co‐immunoprecipitation assays to assess whether MUC13 directly interacts with CALR/mobilferrin. No evidence of co‐immunoprecipitation was observed in either LS513 cells with endogenous MUC13 or SW480 cells with vector‐driven MUC13 expression under low or high iron conditions (Figure S9).

We next sought to identify MUC13‐interacting proteins using an unbiased MS‐based immunoprecipitation proteomic approach in *MUC13*‐overexpressing SW480 cells and SW480 vector control cells. Ingenuity Pathway Analysis identified 37 high‐confidence protein candidates in *MUC13*‐overexpressing cells (Figure S10A and Table S15). Among them, integrin alpha‐6 (ITGA6) and integrin beta‐4 (ITGB4) were associated with the integrin signaling pathway, which showed the highest enrichment (z score, 3.545; *p* = 4.17 × 10^−11^) (Figure S10B). Of the 26 proteins in the integrin signaling pathway, ITGA6 and ITGB4 showed the largest fold changes, with approximately 46‐fold and 38‐fold increases, respectively (Table S16). IPA‐tox analysis identified liver inflammation, cirrhosis, hyperproliferation, and hepatocellular carcinoma as major associated biological functions (Figure S10C,D). These results suggest that MUC13 may interact with integrin family proteins and contribute to Fe^3+^ uptake through the mucin‐integrin‐CALR/mobilferrin pathway.

### 3.8 MUC13 Promotes Ferric Iron Uptake in Human Intestinal Cells

Iron treatment increased intracellular FTH1 levels dose‐dependently in both LS513 and SW480 cells, with significantly greater increases in cells expressing higher MUC13 levels (Figure 4A–D). Compared to control siRNA treatment, *MUC13* knockdown in LS513 cells resulted in a significant decrease in iron uptake (Figures 4A,C and Figure S11A). Consistent with this, MUC13 overexpression promoted iron uptake in SW480 cells (Figures 4B,D and Figure S11B). Iron concentration in the culture supernatant was higher in *MUC13* siRNA LS513 cells than in control cells on day 4, consistent with reduced iron uptake (Figure 4E). Conversely, iron concentration was lower in *MUC13*‐overexpressing SW480 cells than in control cells on day 3, consistent with increased iron uptake (Figure 4F).

**FIGURE 4:**
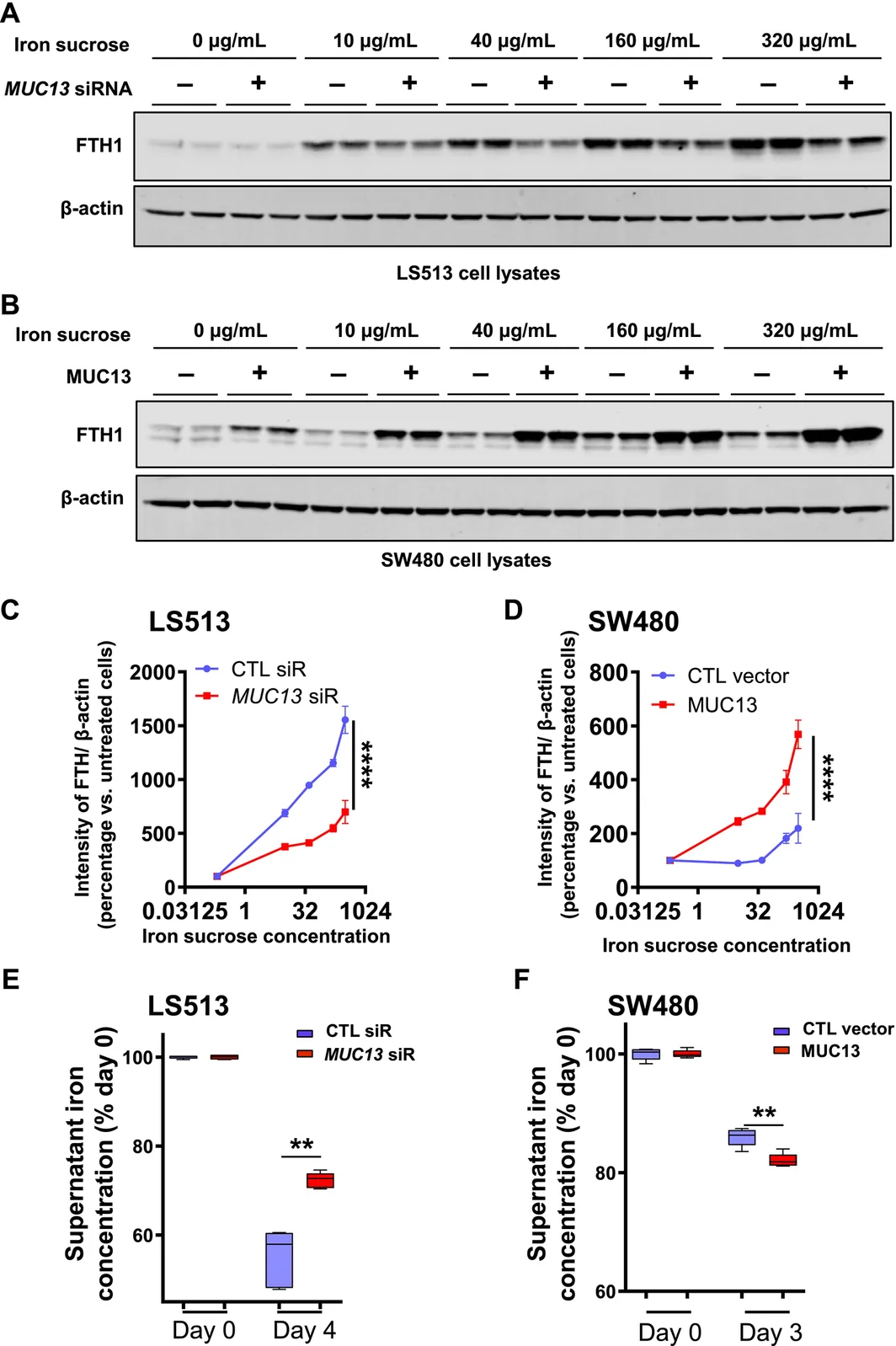
MUC13 promotes ferric iron (Fe^3+^) uptake and results in higher iron storage protein (FTH1) levels in response to iron sucrose treatment in human intestinal epithelial cells. (A) LS513 cells were transfected with *MUC13* or control siRNA for 48 h and then treated for an additional 6 h with the indicated concentration of iron sucrose (Fe^3+^). Western blot analysis of LS513 cell lysates was performed for FTH1 (*n* = 2). (B) Control and MUC13‐overexpressing SW480 cells were treated for 6 h with the indicated concentration of iron sucrose (Fe^3+^). Western blot analysis of FTH1 levels in the SW480 cell lysates was performed (*n* = 2). (C) Densitometric analysis of the blots shown in 4A and Figure S11A (*n* = 4 in total) reporting the amount of FTH1 normalized to β‐Actin and expressed as a percentage of the expression in untreated cells. (D) Densitometric analysis of Figure 4B and Figure S11B (*n* = 4 in total) reporting the amount of FTH1 normalized to β‐Actin and expressed as a percentage of the expression in untreated cells. (E, F) Supernatants were collected at the indicated time, and iron concentrations were measured and expressed as a percentage of the expression on day 0. (E) LS513 cells were transfected with *MUC13* or control siRNA for 48 h, and then 4 × 10^6^ control or *MUC13* siRNA cells were seeded into 90 mm plates and treated with 320 μg/mL iron sucrose at day 0 (*n* = 5). (F) A total of 4 × 10^6^ control or MUC13‐overexpressing SW480 cells were seeded into 90 mm plates and treated with 320 μg/mL iron sucrose at day 0 (*n* = 5). Statistics: Mean ± SEM (*n* ≥ 4), 2‐way ANOVA with Tukey multiple comparisons test (C, D); One‐way ANOVA with Kruskal‐Wallis test (E, F), **p < 0.01, ****p < 0.0001. See Figure S11 for the replicate experiments (*n* = 2) contributing to the quantitative assessments.

### 3.9 *MUC13* Silencing Protects Against Ferric Iron Toxicity

Having established the importance of MUC13 in promoting iron uptake in human intestinal cells, we next assessed the impact of MUC13 modulation on iron‐induced toxicity. Exposure to iron sucrose (320 μg/mL) significantly impaired proliferation of control LS513 cells but not of *MUC13* siRNA‐treated cells, indicating protection against iron toxicity (Figure 5A). Conversely, *MUC13* overexpression in SW480 cells potentiated iron‐induced growth inhibition (Figure 5B).

**FIGURE 5:**
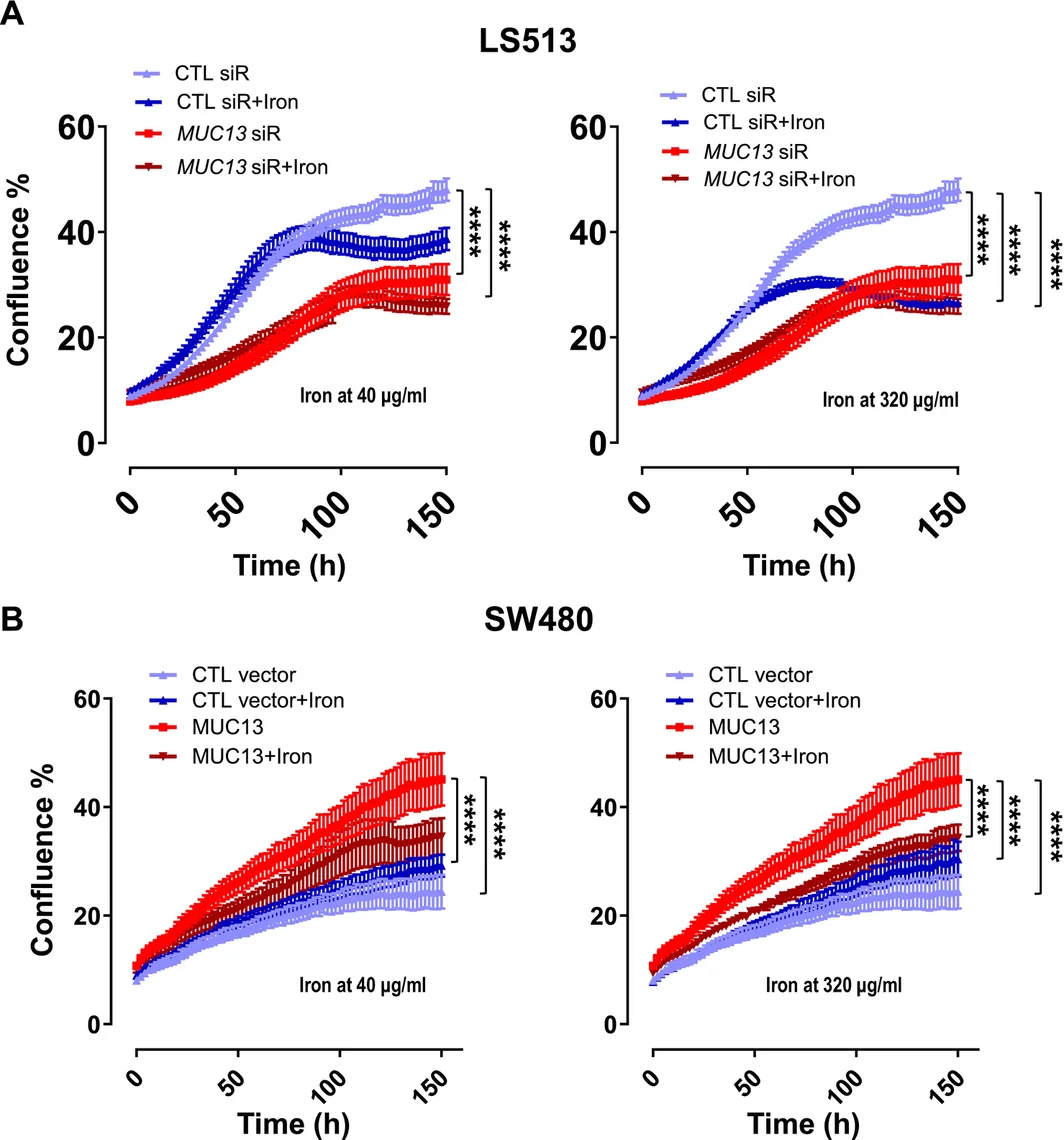
*MUC13* silencing protects human intestinal cells from ferric iron (Fe^3+^) toxicity, and its overexpression potentiates ferric iron (Fe^3+^) toxicity. (A) LS513 cells were transfected with *MUC13* or control siRNA for 48 h, and then 1 × 10^4^ control or *MUC13* siRNA cells were seeded into 96‐well plates and treated with 40 or 320 μg/mL iron sucrose or vehicle. Real‐time cell growth was monitored in the IncuCyte S3 Live‐Cell Analysis Instrument. (B) A total of 1 × 10^4^ control or MUC13‐overexpressing SW480 cells were seeded into 96‐well plates and treated with 40 or 320 μg/mL iron sucrose or vehicle. Real‐time cell growth was monitored in the IncuCyte S3 Live‐Cell Analysis Instrument. Statistics: Mean ± SEM (*n* = 4), 2‐way ANOVA with Tukey multiple comparisons test. * *p* < 0.05 vs. control siRNA (CTL siR) or control (CTL) vector. ****p < 0.0001.

### 3.10 The *MUC13* 503Ser/Ser Variant Increases Cell Surface MUC13 Expression and Iron Uptake

Site‐directed mutagenesis was used to generate the *MUC13* p.Arg503Ser variant in SW480 cells. Flow cytometry revealed significantly higher cell surface MUC13 expression in 503Ser/Ser cells compared with 503Arg/Arg (wild‐type) cells, with no difference in total cellular MUC13 levels (Figure 6A). Following iron exposure (320 μg/mL), 503Ser/Ser cells exhibited significantly higher levels of FTH1, CALR, and NRF2 compared with wild‐type cells (Figure 6B,C and Figure S12). No significant effect was observed on transferrin receptor protein levels. These findings support a causal relationship between the *MUC13* 503Ser/Ser genotype and enhanced intestinal ferric iron uptake.

**FIGURE 6:**
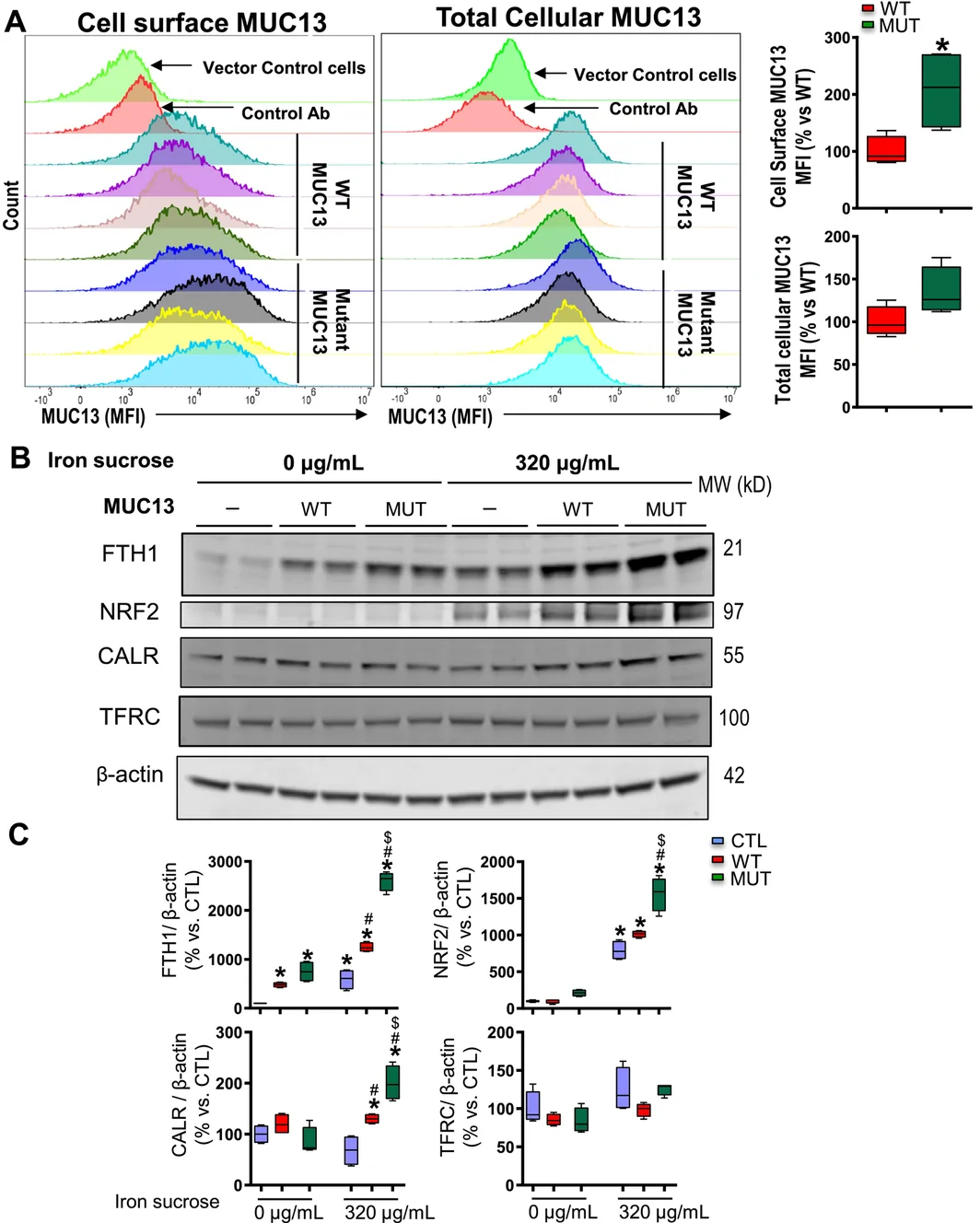
Mutant *MUC13* 503Ser/Ser human intestinal cells overexpress MUC13 on their surface and have increased iron uptake compared with *MUC13* WT cells. Evaluation of MUC13 expression by flow cytometry in MUC13 WT (503Arg/Arg) or mutant (503Ser/Ser)‐expressing intestinal cells. (A) Vector control, WT MUC13‐overexpressing (WT), and mutant 503Ser/Ser MUC13‐overexpressing (MUT) SW480 human intestinal epithelial cells were harvested. The amounts of cell surface MUC13 per viable cell and total cellular MUC13 determined in permeabilized cells were measured by flow cytometry using an antibody that detects an N‐terminal FLAG tag on the extracellular domain of MUC13. Median fluorescence intensity (MFI) in viable cells was determined after subtraction of the MFI using a negative control antibody and expressed as the fold difference relative to the mean MFI of WT cells. Representative flow cytometry histograms are shown. Each replicate represents an independent pool of clones selected after transfection, allowing multiple assessments of gene expression. (B) Cells from A were untreated or treated with 320 μg/mL iron sucrose (Fe^3+^) for 6 h. Western blot analysis of SW480 cell lysates (*n* = 2) was performed with the indicated antibodies. (C) Densitometric analysis of Figure 6 and Figure S12 (*n* = 4) showing the amount of FTH1, NRF2, CALR, and transferrin receptor (TFRC) normalized to β‐Actin and expressed as a percentage of level measured in untreated vector cells. Data are presented as box and whisker plots displaying the median, quartiles, and range; Mann–Whitney *U* test (A, C), * *p* < 0.05 vs. untreated vector cells, # *p* < 0.05 vs. Fe^3+^‐treated vector cells, $ *p* < 0.05 vs. Fe^3+^‐treated WT cells.

## 4 Discussion

In this study, we identified an association between a common variant in *MUC13* and plasma ferritin levels in patients with unexplained hyperferritinemia. This association was replicated in an independent cohort of patients with unexplained hyperferritinemia in whom comprehensive genetic testing had excluded pathogenic variants in known iron overload genes, with the *MUC13* 503Ser/Ser genotype emerging as the strongest independent predictor of elevated transferrin saturation. These results position *MUC13* within the intestinal Fe^3+^ absorption pathway and link the p.Arg503Ser variant to increased iron uptake. The clinical relevance of the *MUC13* p.Arg503Ser variant extends beyond laboratory abnormalities. In the replication cohort, the majority of patients exhibited hepatic iron overload on MRI, with one‐third demonstrating severe iron accumulation. Although we did not assess clinical liver disease outcomes directly (e.g., fibrosis staging, cirrhosis), the association between the 503Ser/Ser genotype and elevated transferrin saturation suggests a mechanism that could contribute to progressive hepatic iron accumulation over time. Future longitudinal studies should evaluate whether *MUC13* genotyping can identify patients at higher risk of liver disease progression among those with unexplained hyperferritinemia.

The causal effect of MUC13 modulation on Fe^3+^ absorption was confirmed in intestinal cells, with downstream effects on three major proteins involved in iron transport and metabolism. Ferritin heavy chain 1 (FTH1) serves as the principal cellular iron storage protein. Calreticulin, also known as mobilferrin, participates in the mucin‐integrin‐calreticulin/mobilferrin pathway for Fe^3+^ absorption [21, 22] (see Supplementary Appendix for extended references). This molecular chaperone facilitates the folding of proteins synthesized in the endoplasmic reticulum and functions as a stress response protein induced by heat, food deprivation, and chemical insults. Intracellular calreticulin levels correlate with increased intracellular iron and are associated with protection against oxidative stress [23]. Iron‐induced calreticulin expression has been proposed as a component of the cellular response to iron‐induced oxidative stress [22]. Nuclear factor erythroid 2‐related factor 2 (NRF2, encoded by *NFE2L2*) is a transcription factor that controls the expression of detoxification and antioxidant enzymes in response to iron‐induced electrophilic stress [24, 25]. In mice, Nrf2 is activated by iron‐induced, mitochondria‐derived pro‐oxidants and drives *Bmp6* expression and subsequent hepcidin synthesis in hepatic sinusoidal endothelial cells [24]. In our mouse model, *Muc13* ^−/−^ mice tended to exhibit lower plasma hepcidin levels, possibly reflecting compensatory downregulation in response to reduced iron absorption.

Three pathways have been described for iron reduction and absorption by enterocytes. First, Fe^3+^ is enzymatically reduced by duodenal cytochrome b reductase 1 (DCYTB, encoded by *CYBRD1*) and transported by the specific membrane transport molecule divalent metal transporter 1 (DMT1) [26]. Second, heme iron uptake by enterocytes occurs via the proton‐coupled folate transporter (SLC46A1, formerly known as HCP1) [26]. Third, Fe^3+^ is absorbed directly via iron‐binding components, mainly intestinal brush border mucin, through the mucin‐integrin‐calreticulin/mobilferrin pathway [20]. However, while the first two pathways have been extensively characterized at the molecular level, the specific mucin protein involved in direct Fe^3+^ uptake remained unknown. Our findings provide evidence that MUC13 is potentially involved in this pathway.

*MUC13* encodes a transmembrane glycoprotein of the mucin family that is highly expressed in the gastrointestinal tract [19]. In healthy tissues, MUC13 is a substantial constituent of the enterocyte glycocalyx decorating the microvilli [27]. Mucins are large glycoproteins endowed with sulfate and carboxyl groups that play a role in metal binding by forming colloidal polyhydroxy iron cores that prevent further polymerization and precipitation [20]. Fe^3+^, which is insoluble at physiological pH, is released from the dietary matrix in the acidic environment of the stomach and chelated by mucins on the surface of the duodenal brush border, maintaining iron in the trivalent state. The iron‐mucin complex is readily dissociated, and the released Fe^3+^ is available to beta‐integrin for membrane uptake [20]. Fe^3+^ enters enterocytes through the apical membrane via interaction with beta3‐integrin and calreticulin/mobilferrin [21, 22, 23, 28, 29, 30, 31, 32, 33]. In the cytosol, this complex combines with flavin monooxygenase and beta2‐microglobulin to form a 520 kDa protein complex known as paraferritin, which has iron reductase activity to convert the absorbed Fe^3+^ to Fe^2+^ [34, 35, 36, 37, 38]. The paraferritin complex may also contain DMT1, allowing the delivery of Fe^2+^ to intracellular organelles [34]. Calreticulin/mobilferrin has been proposed as a carrier for iron that has recently entered intestinal cells [21, 22].

Like classical immune receptors, the intracellular tails of transmembrane mucins are linked to signaling pathways [27]. MUC13 is phosphorylated in unstimulated intestinal epithelial cells [27, 39]. Phosphorylation of several tyrosine, threonine, and serine residues in the tails of various transmembrane mucins has been confirmed by mass spectrometry, as reported in the PhosphoSitePlus database [27]. The *MUC13* variant identified in the exome association study, p.Arg503Ser, is located in the cytoplasmic domain of MUC13. According to the PhosphoSitePlus database, the cytoplasmic domain of MUC13 contains eight phosphorylation sites [27]. The p.Arg503Ser variant may introduce an additional phosphorylation site at position 503 by replacing arginine with serine, potentially increasing the fitness of intestinal epithelial cells by altering the activities of signaling pathways that regulate apoptosis and proliferation [40]. In silico analyses with NetPhos 3.1 and GPS 6.0 predicted a phosphorylation site at position 503 in the variant sequence (NetPhos score, 0.801 [threshold, 0.5]; GPS score, 0.680 [cutoff, 0.504]), with no corresponding prediction at the arginine residue of the reference sequence. This is consistent with the recent demonstration that the MUC13 cytoplasmic tail contains a predicted protein kinase C (PKC) consensus phosphorylation motif and that deletion of the cytoplasmic tail alters PKC‐dependent tight junction regulation and barrier permeability in intestinal epithelial cells [41]. MUC13 was the first transmembrane mucin shown to negatively regulate tight junction function and increase intestinal epithelial barrier permeability, establishing it as a central modulator of paracellular transport in the intestinal epithelium [41]. Whether the p.Arg503Ser substitution modifies PKC‐mediated signaling through the MUC13 cytoplasmic tail remains to be determined. An alternative consequence of altered phosphorylation is the inhibition of enterocyte shedding, which could lead to iron accumulation.

MUC13 also promoted intestinal Fe^3+^ uptake, suggesting one mechanism by which these events may contribute to iron overload. An alternative hypothesis is that the *MUC13* p.Arg503Ser variant increases the activity of the mucin‐integrin‐calreticulin/mobilferrin pathway. We demonstrated a causal association between the 503Ser/Ser genotype and increased MUC13 expression on the surface of intestinal epithelial cells. The p.Arg503Ser variant has the potential to enhance mucin stability on the cell surface by either facilitating intracellular interactions with cytoskeletal proteins within the microvilli or diminishing interactions with proteins involved in mucin recycling from the cell surface. This interpretation is supported by the finding that MUC13 localizes not only to the apical surface but also to the tight junction region of the lateral membrane in intestinal epithelial cells and that its cytoplasmic tail is required for the regulation of tight junction protein composition and paracellular permeability [41]. Proteomic analysis revealed significant enrichment of two transmembrane receptor integrin proteins (ITGA6 and ITGB4) in MUC13‐overexpressing cells. Because beta‐integrins are established components of the mucin‐integrin‐calreticulin/mobilferrin pathway for Fe^3+^ uptake [20, 21, 32], the identification of ITGA6 and ITGB4 as MUC13‐associated proteins is consistent with a model in which MUC13 modulates integrin accessibility and clustering at the enterocyte surface, potentially facilitating Fe^3+^ transfer to the integrin‐calreticulin/mobilferrin complex. To our knowledge, the proteomic enrichment of ITGA6 and ITGB4 in association with MUC13 has not been previously described. Whether MUC13 interacts directly with these integrin subunits or influences their spatial organization indirectly through the enterocyte glycocalyx remains to be determined. Additional evidence supporting the involvement of MUC13 in hyperferritinemia mechanisms in vivo is provided by mass spectrometry‐based immunoprecipitation proteomics on MUC13‐associated proteins, which revealed liver inflammation, liver cirrhosis, hepatic hyperproliferation, and hepatocellular carcinoma as the main associated diseases and biological functions.

In our study, cell surface MUC13 expression was higher in intestinal epithelial cells carrying the p.Arg503Ser substitution, as were FTH1, CALR, and NRF2 levels after iron exposure, consistent with increased Fe^3+^ uptake. Patients with the *MUC13* 503Ser/Ser genotype may therefore absorb more dietary Fe^3+^, with iron accumulating gradually over time. Such gradual accumulation would differ from the iron overload observed in genetic disorders affecting the hepcidin‐ferroportin signaling axis, which are expressed with variable penetrance [2]. The contribution of a common variant such as *MUC13* p.Arg503Ser to iron status would also depend on time and on other determinants of iron balance.

Several limitations should be considered when interpreting our results. First, the discovery cohort comprised patients referred for CES for diverse indications, which could affect the distribution of plasma ferritin levels. However, we addressed this potential source of confounding through both the selection criteria and the analytical strategy. Known genetic and acquired causes of elevated ferritin were excluded before analysis, and association testing was adjusted for potential confounders (age, sex, ASAT, ALAT, C‐reactive protein, history of chronic liver disease, and the top 5 principal components from the ancestry inference analysis). Among the 11 cross‐validated variants, only *MUC13* p.Arg503Ser was associated with plasma iron and transferrin saturation, and none of the 11 variants was associated with body mass index, alanine aminotransferase level, or C‐reactive protein level, indicating that the ferritin associations were not confounded by metabolic syndrome, hepatic cytolysis, or inflammation. Second, although we validated our findings in an independent replication cohort, further confirmation in additional populations with different ethnic backgrounds would strengthen the generalizability of our results. Third, we did not evaluate the effects of other potential *MUC13* variants on iron metabolism or the interaction between the 503Ser/Ser variant and variants of other genes involved in iron metabolism. Future studies should evaluate the effects of different *MUC13* variants and the effect of changes in the functional domains of the protein on intestinal iron absorption. Fourth, future studies using primary intestinal organoids would complement our findings, which were consistent across knockout mice, siRNA silencing, overexpression, and site‐directed mutagenesis in intestinal cell lines. Fifth, the co‐immunoprecipitation experiments did not demonstrate a direct physical interaction between MUC13 and calreticulin, which suggests that the functional connection between these proteins may be indirect, potentially mediated through integrin complexes or other intermediate adaptor proteins. Sixth, ITGA6 and ITGB4 were identified among high‐confidence protein candidates using an unbiased MS‐based immunoprecipitation proteomic approach in *MUC13*‐overexpressing cells. Whether these integrin subunits mediate the MUC13‐dependent increase in Fe^3+^ uptake was not tested. Targeted co‐immunoprecipitation of MUC13 with these subunits and integrin knockdown combined with iron uptake assays would address the proposed mucin‐integrin‐calreticulin/mobilferrin mechanism. Seventh, the *MUC13* p.Arg503Ser variant introduces a serine at a position predicted to be phosphorylated. Phosphorylation levels were not compared between intestinal cells expressing the 503Arg/Arg and the 503Ser/Ser genotypes, and quantification of MUC13 phosphorylation in both genotypes would test this prediction. Future clinical studies should address the effect of the *MUC13* p.Arg503Ser variant on hepcidin modulation and the long‐term consequences of hyperferritinemia.

Our study has several strengths. First, it represents the first sequencing‐based exome association study of rare and common genetic variants associated with plasma ferritin levels in patients with unexplained hyperferritinemia. Second, it incorporated a robust bioinformatic approach using gene burden testing, per‐variant testing, and Bayesian analysis of variance inference to assess effect estimates and Bayes factors of the top significant variants associated with plasma ferritin and other iron metabolism markers. Third, the association between the *MUC13* 503Ser/Ser genotype and elevated transferrin saturation was validated in an independent replication cohort of patients with unexplained hyperferritinemia in whom known genetic causes of iron overload had been excluded. Fourth, multiple animal and cellular models demonstrated the modulation of ferric iron transport, absorption, and metabolism by MUC13 and supported a causal relationship between the *MUC13* 503Ser/Ser variant and increased ferric iron absorption by intestinal epithelial cells. Fifth, our results identify MUC13 as the previously unknown mucin component of the Fe^3+^ absorption pathway, providing new insights into a mechanism that has been less explored than DMT1‐mediated Fe^2+^ transport.

In conclusion, the present study provides novel evidence supporting the involvement of MUC13 in intestinal ferric iron uptake and a causal link between the *MUC13* homozygous 503Ser genotype and enhanced cellular iron absorption, which could contribute to a better understanding of unexplained hyperferritinemia.

## Author Contributions

J.C.: Investigation, Writing (original draft), Writing (review and editing), Visualization. Y.H.S.: Investigation, Resources, Writing (original draft), Writing (review and editing), Visualization. Y.H.: Investigation, Resources, Writing (review and editing). M.S.R.: Investigation, Resources, Writing (review and editing). C.C.: Investigation, Resources, Writing (review and editing). R.S.: Investigation, Resources, Writing (review and editing). F.B.: Investigation, Resources, Writing (review and editing). M.B.: Investigation, Resources, Writing (review and editing). R‐M.G‐R.: Investigation, Resources, Writing (review and editing). F.N.: Investigation, Writing (review and editing). G.G.: Investigation, Resources, Writing (original draft), Writing (review and editing), Visualization. J.L.H.: Investigation, Resources, Writing (review and editing). H.M.: Investigation, Resources, Writing (review and editing). T.L.: Investigation, Resources, Writing (review and editing). D.T.: Investigation, Resources, Writing (review and editing). H.P.: Investigation, Resources, Writing (review and editing). T.H.F.: Investigation, Resources, Writing (review and editing). J.B.: Investigation, Resources, Writing (review and editing). J.D.H.: Investigation, Resources, Writing (review and editing). J‐P.B.: Investigation, Resources, Writing (review and editing). M.A.M.: Investigation, Resources, Writing (review and editing), Visualization. J‐L.G.: Investigation, Resources, Writing (original draft), Writing (review and editing), Funding acquisition. A.O.: Conceptualization, Methodology, Software, Validation, Formal analysis, Investigation, Resources, Writing (original draft), Writing (review and editing), Visualization, Supervision, Project administration, Funding acquisition.

## Funding

This work was supported by INSERM UMR_S 1256, Nutrition, Genetics, and Environmental Risk Exposure (NGERE); and the Department of Genomic Medicine, Division of Biochemistry, Molecular Biology, and Nutrition, University Hospital of Nancy.

## Disclosure

Both male and female mice were included in the animal experiments. The human clinical cohorts included both male and female participants.

## Ethics Statement

Ethical approval for this study (approval no. 2020/264) was provided by the Ethics Committee of the University Hospital of Nancy. The Nancy Biochemical Database is registered with the French National Commission on Informatics and Liberty (CNIL N°1763197v0). Written informed consent was obtained from all participants. All animal experiments were approved by the University of Queensland Animal Experimentation Ethics Committee.

## Conflicts of Interest

A.O. reports consulting fees from Mirum, Dexter, and Atheneum Partners; lecture fees from Bayer Healthcare and Mirum; travel support from Bayer Healthcare, Gilead Sciences, Mirum, AstraZeneca, Pfizer, and Thermo Fisher Diagnostics; and two pending patents: DNA Methylation Signature for Diagnosing Hepatocellular Carcinoma (EP21306459) and Computer‐Implemented Method for Quality Control Processing of High‐Throughput Sequenced Genomic Data (EP24306620), filed with INSERM Transfert, Satt, Incubateur Lorrain, and CHRU Nancy. J.‐P.B. reports grants from Roche, AstraZeneca, and MSD (payments to institution); consulting fees from Roche, AstraZeneca, and Bayer; lecture fees from Roche, AstraZeneca, and Bayer; and travel support from Roche and AstraZeneca. M.S.R. reports travel support from Ipsen. J.C., Y.H.S., Y.H., C.C., R.S., F.B., M.B., R.‐M.G.‐R., F.N., G.G., J.L.H., H.M., T.L., D.T., H.P., T.H.F., J.B., J.D.H., M.A.M., and J.‐L.G. declare no conflicts of interest.

## Supporting information


**Appendix S1:** Supplementary appendix.

## Data Availability

Under French law, genetic data are subject to specific regulatory restrictions that preclude their sharing outside the institution where they were generated. Anonymized clinical and biochemical data from this study may be available to qualified researchers upon reasonable request. Requests will be reviewed by the Institutional Review Board of the University Hospital of Nancy and require submission of a research proposal, a statistical analysis plan, and execution of a data‐sharing agreement.
