# Supplementary material for: A MUC13 Variant Enhances Intestinal Ferric Iron Absorption in Unexplained Hyperferritinemia
Source: Liver Int. 2026 Oct 26;46(11):e70901. doi: 10.1111/liv.70901 (PMC13702042; doi:10.1111/liv.70901)
Supplement: Supplementary file 1 [file LIV-46-0-s001.zip › liv70901-sup-0001-TableS1-S16-FigureS1-S12@Supplementary appendix_3338727.docx]

**SUPPLEMENTARY APPENDIX**

**Article title**

A *MUC13* variant enhances intestinal ferric iron absorption in unexplained hyperferritinemia

**Short title**

*MUC13* and intestinal ferric iron absorption

**Author names and affiliations**

Jonas Callet, MD^1*^; Yong H. Sheng, PhD^2*^; Yaowu He, PhD^3^; Maël Silva Rodriguez, MD, MSc^1,4,5^; Céline Chéry, PhD^1,4,5^; Ramia Safar, PhD^4^; Françoise Barbé, PharmD, PhD^1^; Mouni Bensenane, MD^6^; Rosa-Maria Guéant-Rodriguez, MD, PhD^1,4,5^; Fares Namour, MD, PhD^1,4,5^; Guillaume Gauchotte, MD, PhD^4,7^; Janelle Louise Hancock, PhD^8^; Hana Manceau, PharmD, PhD^9,10^; Thibaud Lefebvre, PharmD, PhD^9,11^; Dimitri Tchernitchko, MD, PhD^9^; Hervé Puy, MD, PhD^9,11^; Timothy H. Florin, MD, PhD^12^; Jakob Begun, MD, PhD^12^; John D. Hooper, PhD^3^; Jean-Pierre Bronowicki, MD, PhD^4,6^; Michael A. McGuckin, PhD^2,13^; Jean-Louis Guéant, MD, DSc, AGAF^14^; and Abderrahim Oussalah, MD, PhD^1,4,5^

** Authors with equal contribution*

^1^ Department of Molecular Medicine, Division of Biochemistry, Molecular Biology, Nutrition, and Metabolism, University Hospital of Nancy, F-54000 Nancy, France

^2^ QIMR Berghofer Medical Research Institute, Cancer Program, Herston, Queensland 4006, Australia

^3^ Cancer Biology Group, Mater Research Institute-University of Queensland, Woolloongabba, Queensland 4101, Australia

^4^ University of Lorraine, INSERM UMR_S 1256, Nutrition, Genetics, and Environmental Risk Exposure (NGERE), Faculty of Medicine of Nancy, F-54000 Nancy, France

^5^ Reference Center for Inborn Errors of Metabolism (ORPHA67872), University Hospital of Nancy, F-54000 Nancy, France

^6^ Department of Gastroenterology and Liver Diseases, University Hospital of Nancy, F-54000 Nancy, France

^7^ Department of Pathology, University Hospital of Nancy, F-54000 Nancy, France

^8^ Proteomics Core Facility, Translational Research Institute, 37 Kent Street, Woolloongabba, QLD 4102, Australia

^9^ Centre de Recherches sur l'Inflammation, INSERM U1149, Université Paris Cité, 75018 Paris, France

^10^ Laboratory of Biochemistry, Beaujon University Hospital, Assistance Publique-Hôpitaux de Paris, 92110 Clichy, France

^11^ Centre Français des Porphyries, Hôpital Louis Mourier, Assistance Publique-Hôpitaux de Paris, 178 rue des Renouillers, 92701 Colombes, France

^12^ Inflammatory Bowel Diseases Group, Mater Research Institute-The University of Queensland, Translational Research Institute, Woolloongabba, Queensland 4102, Australia

^13^ Faculty of Medicine, Dentistry and Health Sciences, The University of Melbourne, Parkville, VIC 3010, Australia

^14^ Joan Klein Jacobs Center for Precision Nutrition and Health, Cornell University, Ithaca, NY, USA

**Corresponding authors**

**Prof. Abderrahim Oussalah, MD, PhD, FEBGH**

Department of Genomic Medicine, Division of Biochemistry, Molecular Biology, and Nutrition, University Hospital of Nancy, INSERM UMR_S 1256, Nutrition, Genetics, and Environmental Risk Exposure (NGERE), Faculty of Medicine of Nancy, 9 Avenue de la Forêt de Haye, F-54000 Nancy, France

Telephone: +33 3 83 15 36 29

E-mail: abderrahim.oussalah@univ-lorraine.fr

**Dr. Yonghua Sheng, PhD**

QIMR Berghofer Medical Research Institute, Cancer Program, Herston, Queensland 4006, Australia

Telephone: +61 0419757550

E-mail: yong.sheng@mater.uq.edu.au

**SUMMARY**

[SUPPLEMENTARY METHODS	7](#_Toc238140045)

[French framework for genetic diagnosis	7](#_Toc238140046)

[Data collected for the study.	7](#_Toc238140047)

[Next-generation sequencing.	7](#_Toc238140048)

[Quality controls on genetic data and variant filtering.	8](#_Toc238140049)

[Muc13 knockout mouse model.	9](#_Toc238140050)

[Biochemical assays on samples from *Muc13-/-* and *Muc13+/+* mice.	9](#_Toc238140051)

[Histopathology of liver biopsies from *Muc13-/-* and *Muc13+/+* mice.	9](#_Toc238140052)

[Isolation of duodenal epithelial cells from *Muc13-/-* and *Muc13+/+* mice.	10](#_Toc238140053)

[Cell culture, *MUC13* siRNA silencing, and *MUC13* vector transfection.	10](#_Toc238140054)

[Western blotting.	11](#_Toc238140055)

[Co-immunoprecipitation.	11](#_Toc238140056)

[Proteomic analysis of MUC13-associated proteins.	12](#_Toc238140057)

[Determination of iron in cell culture supernatants.	13](#_Toc238140058)

[Cell growth analysis.	13](#_Toc238140059)

[Generation of the *MUC13*-Arg503Ser variant by site-directed mutagenesis.	13](#_Toc238140060)

[Evaluation of *MUC13* expression by flow cytometry in intestinal cells expressing *MUC13* WT (503Arg/Arg) or mutant (503Ser/Ser).	14](#_Toc238140061)

[Statistical analysis.	14](#_Toc238140062)

[Study design and setting of the replication study	16](#_Toc238140063)

[MUC13 genotyping in the replication study	16](#_Toc238140064)

[Statistical analysis of the replication study	17](#_Toc238140065)

[In silico prediction of phosphorylation sites	17](#_Toc238140066)

[Artificial intelligence disclosure	17](#_Toc238140067)

[Supplementary Table S1. List of the 4733 genes assessed in the exome-based association study	18](#_Toc238140068)

[Supplementary Table S2. Primers used for site-directed mutagenesis to generate the *MUC13* missense variant (p.Arg503Ser)	28](#_Toc238140069)

[Supplementary Table S3. Descriptive statistics of biochemical parameters in the study cohort	29](#_Toc238140070)

[Supplementary Table S4. Results of the clinical exome-based association study reporting the top loci associated with plasma ferritin levels in combined multivariate and collapsing regression analysis	30](#_Toc238140071)

[Supplementary Table S5. Results of the Bayesian analysis of variance for the association between the 11 cross-validated genetic variants and plasma iron concentration	34](#_Toc238140072)

[Supplementary Table S6. Results of the Bayesian analysis of variance for the association between the 11 cross-validated genetic variants and transferrin saturation	35](#_Toc238140073)

[Supplementary Table S7. Results of the Bayesian analysis of variance for the association between the 11 cross-validated genetic variants and plasma transferrin concentration	36](#_Toc238140074)

[Supplementary Table S8. Results of the Bayesian analysis of variance for the association between the 11 cross-validated genetic variants and body mass index	37](#_Toc238140075)

[Supplementary Table S9. Results of the Bayesian analysis of variance for the association between the 11 cross-validated genetic variants and alanine aminotransferase	38](#_Toc238140076)

[Supplementary Table S10. Results of the Bayesian analysis of variance for the association between the 11 cross-validated genetic variants and C-reactive protein	39](#_Toc238140077)

[Supplementary Table S11. Baseline characteristics of patients with unexplained hyperferritinemia in the replication cohort	40](#_Toc238140078)

[Supplementary Table S12. Distribution of genetic, demographic, and biochemical characteristics according to transferrin saturation in the replication cohort	41](#_Toc238140079)

[Supplementary Table S13. Multivariable logistic regression analysis of predictors of elevated transferrin saturation (greater than 55%) in the replication cohort	42](#_Toc238140080)

[Supplementary Table S14. Assessment of iron metabolism markers and liver histopathology in *Muc13*^−/−^ and *Muc13*^+/+^ mice	43](#_Toc238140081)

[Supplementary Table S15. High-confidence protein candidates identified in association with *MUC13* overexpression using Ingenuity Pathway Analysis	44](#_Toc238140082)

[Supplementary Table S16. Proteins associated with the integrin signaling pathway identified by Ingenuity Pathway Analysis	45](#_Toc238140083)

[Supplementary Figure S1. Flow diagram of patient selection	47](#_Toc238140084)

[Supplementary Figure S2. Principal component analysis according to patient ancestry	48](#_Toc238140085)

[Supplementary Figure S3. The exome association study identified several genes associated with plasma ferritin levels.	49](#_Toc238140086)

[Supplementary Figure S4. Results of the Bayesian analysis of variance for the association between the 11 cross-validated genetic variants and plasma iron concentration	51](#_Toc238140087)

[Supplementary Figure S5. Results of the Bayesian analysis of variance for the association between the 11 cross-validated genetic variants and transferrin saturation	53](#_Toc238140088)

[Supplementary Figure S6. Results of the Bayesian analysis of variance for the association between the 11 cross-validated genetic variants and plasma transferrin concentration	55](#_Toc238140089)

[Supplementary Figure S7. Representative immunohistochemistry images of small intestine sections from *Muc13*^+/+^ and *Muc13*^−/−^ mice stained with anti-MUC13 antibody	57](#_Toc238140090)

[Supplementary Figure S8. Assessment of liver histopathology in *Muc13*^−/−^ and *Muc13*^+/+^ mice	58](#_Toc238140091)

[Supplementary Figure S9. Co-immunoprecipitation analysis of the interaction between *MUC13* and calreticulin	60](#_Toc238140092)

[Supplementary Figure S10. Ingenuity Pathway Analysis of *MUC13*-associated proteins	62](#_Toc238140093)

[Supplementary Figure S11. *MUC13* promotes ferric iron uptake and increases ferritin heavy chain expression in response to iron sucrose treatment in intestinal epithelial cells	64](#_Toc238140094)

[Supplementary Figure S12. Effect of the *MUC13* p.Arg503Ser variant on ferritin heavy chain expression in response to iron treatment	65](#_Toc238140095)

[Extended references for the mucin-integrin-calreticulin/mobilferrin pathway	66](#_Toc238140096)

[REFERENCES	67](#_Toc238140097)

# SUPPLEMENTARY METHODS

# French framework for genetic diagnosis

In France, patients with suspected genetic disorders are referred to specialized reference centers established under the French National Plan for Rare Diseases. The Reference Center for Inborn Errors of Metabolism at the University Hospital of Nancy (ORPHA67872) serves as one such center, receiving patients referred by their treating physicians for genetic evaluation. Written informed consent is obtained prior to genetic testing, which is fully reimbursed by the French health care system ^1-3^.

# Data collected for the study.

The following CES-related administrative and clinical data were collected by electronic chart review using DxCare® software (Dedalus France, Le Plessis Robinson, France) for patients followed at the University Hospital of Nancy or by physician prescription for patients outside the University Hospital of Nancy: **demographics**: age at CES prescription and sex; **clinical findings**: weight (kg), height (m), history of chronic kidney disease, history of blood transfusion, previous genetic diagnosis related to iron overload, and history of a severe chronic inflammatory disease; **biological findings**: ferritin (µg/L), iron (mg/L), transferrin (g/L), transferrin saturation (%), ASAT (U/L), ALAT (U/L), γ-glutamyltransferase (U/L), and alkaline phosphatase (U/L) (for patients with multiple encounters, serial biochemical measurements were considered); **iron-related therapies**: history of oral or intravenous iron therapy, erythropoietin therapy, and blood removal for iron overload.

# Next-generation sequencing.

We performed CES using the TruSight One panel on an Illumina MiSeq platform (Illumina, Evry, France) or the TruSight One expanded panel on an Illumina NextSeq 550 platform, in accordance with the requirements of the French Accreditation Committee at the University Hospital of Nancy and the Functional Genomics Facility of INSERM Unit UMR_S 1256 (NGERE) (UMS2008/US40 IBSLor) as previously described ^1,4-6^. At the Department of Molecular Medicine of the University Hospital of Nancy and the National Reference Center for Inborn Errors of Metabolism, we opted for a strategy based on Illumina TruSight captures, which covers many disease-associated genes, instead of a full-exome-based approach ^1^. The TruSight One Sequencing Panel provides comprehensive coverage of 4813 disease-associated genes, while the TruSight One Expanded Sequencing Panel targets ~1900 additional genes with recent disease associations in the scientific literature. The two captures cover 4733 common genes. The list of genes is available in **Supplementary Table 1**. We used Nextera and Nextera Flex enrichment solutions with TruSight One and TruSight One Expanded captures, respectively. Bioinformatics analysis was performed as previously described ^5-8^. The Burrows–Wheeler Aligner (BWA) software package was used to align all reads to the GRCh37/hg19 annotation using the default parameters of the BWA-MEM algorithm. PCR duplicates were removed from the alignments using the Picard (MarkDuplicates) tool (http://broadinstitute.github.io/picard). Indels were realigned using the Genome Analysis Toolkit (GATK) ^9^. SNVs and short indels were called using GATK HaplotypeCaller software ^9^. Copy number variants were not assessed.

# Quality controls on genetic data and variant filtering.

We performed ancestry inference using principal component analysis (PCA) on the samples merged with the 1092 subjects from the 1kG phase 1 superpopulations as reference populations (Africans, n = 246; Admixed Americans, n = 181; Asians, n = 286; and Europeans, n = 379) (data available at: http://www.internationalgenome.org/data/). PCA was performed under the additive model to find up to 10 components (eigenvalues). Duplicated samples and those with cryptic relatedness were assessed by calculating identity by descent (IBD). By estimating the probability of sharing zero, one, or two alleles for any two individuals as P(Z=0), P(Z=1), and P(Z=2), respectively, a proportion of IBD was calculated using the following formula: PI-HAT = PI = P(Z=1)/2 + P(Z=2). A PI-HAT threshold >0.2 was considered to suggest cryptic relatedness.

We used the following strategy for variant and sample selection. Step #1: Import all retrieved genetic variants. Step #2: Retain genetic variants located in genomic regions overlapping the TruSight One and TruSight One expanded captures. Step #3: Exclude synonymous variants using the RefSeq Genes 105v2, NCBI gene source annotation. Step #4: Exclude variants with a mean GQ score (GQx̄) <85. Step #5: Exclude variants with a GQ score variance (GQσ^2^) > 800. Step #6: Exclude monomorphic variants. All genetic variants of interest were confirmed to meet validated quality metrics (read depth [RD] >20, Alt-Read ratio [ARR] >0.2, genotype quality score [GQ] of 99). All genetic variants retained in the bioinformatic analysis were confirmed by visual inspection of the read-level data of the BAM files. Functional variant annotation was performed with the following tools: MutationTaster ^10^, Functional Analysis through Hidden Markov Models (FATHMM) ^11^, MetaSVM/MetaLR ^12^, Sorting Tolerant From Intolerant (SIFT) ^13^, Protein Variation Effect Analyzer (Provean) ^14^, and Deleterious Annotation of genetic variants using Neural Networks (DANN) ^15^. Variant pathogenicity was reported according to the ACMG criteria ^16^. All retained and top-priority variants were assessed for their ClinVar annotation ^17^. All bioinformatic analyses were performed using SNP & Variation Suite (v8.9.0; Golden Helix, Inc., Bozeman, MT, USA).

# Muc13 knockout mouse model.

The generation of *Muc13-/-* mice in the C57BL/6 background was described previously ^18^. *Muc13*-/- mice and their *Muc13*+/+ (wild type) littermates were bred at the Mater Research Institute, The University of Queensland. All experiments involving mice were conducted in accordance with the Australian Code for the Care and Use of Animals for Scientific Purposes 8th edition (2013) and were approved by the University of Queensland Animal Experimentation Ethics Committee. Mice were sex- and age-matched within experiments (12-13 weeks of age; *Muc13-/-*, n = 6; *Muc13*+/+, n = 7).

# Biochemical assays on samples from *Muc13-/-* and *Muc13+/+* mice.

Mouse liver tissues were collected and weighed. Water was added (100 µL of water per 10 mg of tissue), and samples were homogenized using a tissue homogenizer (Precellys® 24, Bertin Instruments, Montigny-le-Bretonneux, France) in 2 mL tubes prefilled with 1.4 mm ceramic beads (Precellys lysing kit, Soft tissue homogenizing CK14, Bertin Instruments); three homogenization steps of 60 seconds at 6500 rpm and a 30-second pause between each step were performed. We measured plasma iron and transferrin levels in the mice studied using Atellica immunoassay (IM) and clinical chemistry (CH) multiparametric analyzers (Siemens Healthcare SAS, France), respectively. Plasma ferritin levels were measured using a BECKMAN COULTER AU480 (Beckman Coulter France S.A.S., Villepinte, France). As previously described, we measured plasma hepcidin levels by liquid chromatography coupled to tandem mass spectrometry (LC–MS/MS) ^19^. The liver iron concentration (µmol/L) in the supernatants was determined on an Atellica CH multiparametric analyzer (Siemens Healthcare SAS, France).

# Histopathology of liver biopsies from *Muc13-/-* and *Muc13+/+* mice.

Liver tissues were fixed in neutral buffered 4% formalin, embedded in paraffin, sectioned at 5 μm, and subsequently stained with hematoxylin-eosin, saffron (HES), or Sirius red to assess inflammation and fibrosis or Perls Prussian blue staining to assess iron deposition. Sections were examined by light microscopy.

# Isolation of duodenal epithelial cells from *Muc13-/-* and *Muc13+/+* mice.

Small intestinal epithelial cells (IECs) were isolated from the duodenum tissues of *Muc13-/-* and *Muc13+/+* mice, as previously described ^20^. Briefly, *Muc13-/-* and *Muc13+/+* (n=5) were fully backcrossed to a C57BL/6 background and bred in a dedicated pathogen-free animal facility at the Translational Research Institute. The mice were littermates, matched for sex and age, with an age range of 8 to 10 weeks. The duodenum was opened longitudinally, washed in PBS, and cut into 5 mm pieces. The pieces were then incubated in extraction solution (5 mM EDTA and 2 mM DTT in PBS) for 30 min at 37°C with continuous vigorous stirring. The tissue suspension was rapidly filtered through a 40-μm strainer to remove debris and centrifuged at 100 xg for 5 min at 4°C. Dissociated IEC clusters were washed by removing the supernatant. The cells were resuspended in 10 mL DPBS and centrifuged at 100 xg for 5 min at 4°C to recover the enriched IEC population.

# Cell culture, *MUC13* siRNA silencing, and *MUC13* vector transfection.

Human LS513 intestinal epithelial cells (cat no CRL-2134), expressing high levels of MUC13 ^21,22^, and SW480 intestinal epithelial cells (cat no CCL-228), expressing low levels of MUC13 ^22^ were purchased from ATCC (Manassas, Virginia, United States) and cultured in RPM1-1640 (Thermo Scientific, MA, United States) supplemented with 10% heat-inactivated fetal calf serum, 100 U/mL penicillin, 100 µg/mL streptomycin, and 2 mM L-glutamine.

*MUC13 siRNA* silencing. *MUC13* silencing experiments were performed in LS513 cells using SMARTpool and nontargeted control siRNA (Dharmacon™, Lafayette, USA). Oligonucleotide transfection was performed using Lipofectamine™ 2000 transfection reagent (Thermo Scientific) at a final concentration of 100 nM, and cells were harvested for the assays 48 h after transfection.

*MUC13* vector transfection. Human *MUC13* cDNA with a FLAG tag at the N-terminus of the extracellular domain was cloned into a retroviral expression construct (pRUFneo) and then transfected into Bing retrovirus packaging cells using Lipofectamine™ 2000 transfection reagent (Thermo Scientific). Viral supernatants were harvested at 48 and 72 h posttransfection. SW480 cells were infected with filtered viral supernatants in the presence of 4 µg/mL polybrene (Sigma‒Aldrich, Castle Hill, Australia) for 24 h, and then the infected cells were selected in 800 µg/mL geneticin (G418) for five days. Cells that survived geneticin selection were examined for MUC13 expression by Western blot analysis to confirm stable *MUC13* expression.

*MUC13*-silenced and nonsilenced LS513 cells were treated with a range of concentrations of iron sucrose (0, 10, 40, 160, and 320 µg/mL) in the form of polynuclear iron (III) hydroxide (Fe^3+^) core superficially surrounded by a larger number of noncovalently bound sucrose molecules (Venofer, Vifor Pharma Pty Ltd., Melbourne, Australia) for various times as indicated. *MUC13*-overexpressing and control vector-transfected SW480 cells were similarly treated with a range of concentrations of iron sucrose at various times, as indicated.

# Western blotting.

Proteins were extracted from *Muc13-/-* and *Muc13+/+* cells lysed using RIPA buffer containing 20 mM Tris-HCl (pH 7.5), 150 mM NaCl, 1% NP-40, 0.5% sodium deoxycholate, 0.1% SDS and fresh 1x proteinase inhibitor. Protein concentrations were measured using the BCA Protein Assay Kit (Thermo Scientific) with BSA as a standard. Aliquots of the lysate containing 30 µg of total protein were mixed in SDS‒PAGE Laemmli buffer (0.05 M Tris-HCl, pH 6.8, 0.1% 2-mercaptoethanol, 1.0% SDS, 5% glycerol, and 0.15% bromophenol blue), boiled for 5 min, and resolved on 4-12% acrylamide gels. The resolved proteins were transferred to polyvinylidene difluoride (PVDF) membranes and incubated overnight at 4°C in the dark with primary antibodies against ferritin heavy chain (Cat. No.: A19544, ABclonal Technology, Woburn, MA, United States), β-actin (Cat. No.: NB600-501, Novus Biologicals, Centennial, CO, United States), calreticulin (Cat. No.: NB600-101C4, Novus Biologicals), NRF2 (Cat. No.: A21508, ABclonal Technology), MUC13 (Cat. No.: H-300, Santa Cruz Biotechnology, Dallas, Texas, United States) and FLAG (Cat. No.: F7425, Sigma‒Aldrich, St. Louis, MO, United States). IRDye fluorescence-conjugated secondary antibodies were used, and Western blot signals were detected on the Odyssey®DLx imaging system (LI-COR, Lincoln, NE, United States). Densitometric analysis was performed using Image Studio™ Lite Quantification Software (LI-COR).

# Co-immunoprecipitation.

LS513 cells with endogenous MUC13 or SW480 cells with vector-driven MUC13 expression were grown on 10 or 15 cm plates, treated as indicated, washed twice with cold PBS, and harvested with 0.75 or 1.5 ml of Triton X-100 cell lysate buffer (20 mM Tris-HCl, pH 7.5, 150 mM NaCI, 1% Triton X-100, 10% glycerol, 2 mM EDTA with complete protease inhibitor cocktail and PhosSTOP). Cells were lysed on ice for 20 min with stirring every 5 min and centrifuged at 16,000 × g for 20 min at 4°C to remove cell debris. One milligram of protein lysate was used for immunoprecipitation with 3 µg of the following antibodies: calreticulin, MUC13 cytoplasmic domain, and FLAG incubated overnight at 4°C. The next day, 25 µl of Protein A/G UltraLink resin (#53135, Thermo Scientific, MA) was added and incubated for 4 h at 4°C, followed by four washes with lysis buffer. The immunoprecipitate was eluted with Laemmli sample buffer by boiling at 95°C for 5 min. Samples were then analyzed by Western blotting.

# Proteomic analysis of MUC13-associated proteins.

MUC13-overexpressing or vector control SW480 cells were grown to 80% confluence. After several gentle washes in PBS, the cells were harvested. Samples were assayed in biological triplicates. Cells were lysed in TBS with 1% NP40 and protease inhibitor, followed by immunoprecipitation with anti-FLAG antibody (to pull down all proteins interacting with MUC13). The eluents were digested with 40 ng of trypsin overnight and then subjected to mass spectrometry (MS). MS was outsourced to a professional proteomics laboratory with validated protocols (TRI, Qld, Australia).

Briefly, tryptic peptides were resolved by C-18 separation on an UltiMate 3000 HPLC nanoflow HPLC and analyzed on a Q Exactive Plus Orbitrap mass spectrometer running in positive ion data-dependent analysis (DDA) mode, with settings typical for peptide analyses. Raw data were searched against the *Homo sapiens* proteome (Swiss-Prot Taxonomy 9606, database release v2017-10-25) using Proteome Discoverer 2.4. Sample input was normalized to the total peptide amount. Peptide abundance was calculated by precursor intensity. Protein abundance was calculated by summed peptide intensity, and all matching peptides were used for protein quantification. The maximum allowable fold change was set to 100. Abundance comparisons were performed using a background-based *t*-test.

A set of high-confidence proteins physically associated with overexpressed MUC13 was generated by analyzing the entire dataset using two algorithms: false discovery rate (FDR) analysis by Percolator using the reversed concatenated database and peptide ranking by q-value. FDR targets were 0.01 to 0.05 using stringent criteria (FDR: high, noncontaminant, sumPEP score above 20, grouped MUC13 immune complexes (positive samples) abundance above 100000, fold-enrichment in positive samples versus the negative control above 30-fold enriched and detected in all three positive samples).

Ingenuity Pathway Analysis (IPA) (QIAGEN Inc., https://digitalinsights.qiagen.com/IPA) was used to analyze differentially expressed proteins in MUC13-overexpressing and vector control SW480 cells. The Knowledge Base includes biological interaction data for 19,600 human and 14,700 mouse genes. IPA was used to construct molecular networks of direct physical, transcriptional, and enzymatic interactions. Hub proteins that interacted directly with other proteins in IPA were identified. For each hub protein, the specificity of connections was calculated by the percentage of its connections to other significant proteins. Network scores were calculated based on statistical likelihood ^23^. The score indicates the likelihood that the assembly of a set of hub proteins in a network could not be explained by random chance alone.

# Determination of iron in cell culture supernatants.

LS513 cells were transfected with *MUC13* or control siRNA for 48 h, and then 4x10^6^ control or *MUC13* siRNA cells were seeded into 90 mm plates and treated with 320 μg/mL iron sucrose on day 0. *MUC13*-overexpressing and control SW480 cells were seeded into 90 mm plates and treated with 320 μg/mL iron sucrose on day 0. Supernatants were collected every 24 h from day 0, and the iron concentrations were measured on an Atellica clinical chemistry (CH) multiparametric analyzer (Siemens Healthcare SAS, France).

# Cell growth analysis.

The Incucyte® S3 Live-Cell Analysis System (ESSEN BioSCIENCE, Ann Arbor, MI, United States) was used to monitor the proliferation of LS513 cells transfected with *MUC13* or control siRNA for 48 h and *MUC13*-overexpressing or control SW480 cells in real time. Cells were seeded into a 96-well Image Lock plate (ESSEN BioSCIENCE, Ann Arbor, MI), and real-time cell growth was visualized using the IncuCyte® S3 Live-Cell Imaging System. Growth curves were constructed from confluence measurements taken at 2 h intervals throughout the experiment for a total duration of 150 h. Data were then analyzed using a built-in metric that considers the shape of each cell in the field of view. Cell growth analyses were performed on *MUC13*-silenced and nonsilenced cells (LS513) and *MUC13*-overexpressing SW480 cells and control vector-transfected SW480 cells with two iron-sucrose exposure regimens: 40 and 320 μg/mL. Four conditions were compared in each experiment using a factorial design: condition #1: with/without iron sucrose; condition #2: with/without MUC13 silencing (for LS513) or MUC13 overexpression (for SW480 cells).

# Generation of the *MUC13*-Arg503Ser variant by site-directed mutagenesis.

Human *MUC13* cDNA with a FLAG tag at the N-terminus of the extracellular domain was first cloned into a retroviral expression construct (pRUFneo) and then used as a template to generate a *MUC13* missense variant (RefSeq NM_033049.4:c.1509A>C; NP_149038.3:p.Arg503Ser; Ensembl ENST00000311075.3:c.1506A>C, p.Arg502Ser). Site-directed mutagenesis was performed to introduce the *MUC13* mutation p.Arg503Ser using the primers listed in **Supplementary Table S2** and a proofreading polymerase Pfu Ultra (Agilent). The introduction of the desired mutation was confirmed by DNA sequencing at the Australian Genomic Research Facility (St. Lucia, Australia). The *MUC13* missense variant (p.Arg503Ser) was then transfected into Bing retrovirus packaging cells, and the resultant viral supernatants were used to infect SW480 cells as described in METHODS 2.9 and 2.10.

# Evaluation of *MUC13* expression by flow cytometry in intestinal cells expressing *MUC13* WT (503Arg/Arg) or mutant (503Ser/Ser).

For MUC13 detection by flow cytometry, MUC13 WT or MUC13 missense variant (p.Arg503Ser) SW480 cells were harvested with trypsin and stained with BD Horizon^TM^ Fixable Viability Stain 575V (BD) either without fixation (to stain cell surface structures) or after fixation in 1% paraformaldehyde for 10 min on ice and permeabilization with 0.5% saponin (intracellular and extracellular staining) for 10 min on ice. Cells were then incubated with the anti-FLAG antibody (Cat. no.: F3165, Sigma, St. Louis, Missouri, United States) or an isotype control of 401.21 at a final concentration of 10 µg/mL in 1% BSA in PBS for 60 minutes at 4°C, followed by APC-conjugated anti-mouse antibody (Invitrogen). To detect intracellular antigens, washes and antibody incubations were performed in the presence of 0.5% saponin. After staining, all cells were fixed with 2% paraformaldehyde. The analysis was gated to viable cells, and staining scoring was performed on a CytoFLEX flow cytometer (Beckman Coulter, Brea, CA, US) using FlowJo v10 software (FlowJo, LLC, Ashland, Oregon, US).

# Statistical analysis.

Our analytical approach employed a two-stage design combining gene burden testing with per-variant analysis. In the first stage, rare variants (minor allele frequency <1% in gnomAD ^24^) were aggregated within each gene using the combined multivariate and collapsing (CMC) method as described by Li and Leal ^25^. This strategy increases statistical power for detecting gene-level associations when individual rare variants are too infrequent for reliable per-variant testing. In the second stage, per-variant analysis was restricted to variants with minor allele frequency greater than 1% to ensure adequate genotype counts across all three genotype classes (homozygous reference, heterozygous, homozygous alternate) for robust linear regression and Bayesian inference. This threshold is consistent with established practices in exome-wide association studies, where per-variant analysis of rare variants is typically underpowered except in very large sample sizes ^25^. We performed gene-based tests to investigate the influence of rare and low-frequency variants on plasma ferritin levels. Exome data were analyzed using the CMC method with regression analysis ^25^. We collapsed the identified genetic variants using the threshold of alternative allele frequency (AAF) < 0.01 in the gnomAD Exomes Variant Frequencies 2.0.1 v2, BROAD ^24^. We performed CMC linear regression using the following covariates: age, sex, ASAT, ALAT, C-reactive protein, history of chronic liver disease, and the top 5 principal components from the ancestry inference analysis. We used Bonferroni correction to define the most significant loci (Bonf-*P* < 0.05). To increase the power of the study, we used a repeated-measures design, as previously described ^26-28^.

We used linear regression under an additive model to assess the association between genetic variants in Bonferroni-significant loci and plasma ferritin levels. We selected variants with a minor allele frequency > 1% and used the same covariates as those used in the gene-based analysis (CMC regression). For each variant, we reported the full versus reduced model *P* value and the corresponding Bonferroni-corrected *P* value, regression beta coefficient, and standard error (SE).

We used Bayesian analysis of variance (ANOVA) to further assess the validity of the association between Bonferroni-significant variants and plasma ferritin levels and to account for multiple observations ^29,30^. We used genotypes (AA, AB, BB) as fixed factors and the plasma ferritin level as the outcome variable. For each variant, we reported the mean and 95% credible interval (95% Cr.I) per genotype subgroup and the Bayes factor [BF(m)], which represents the change from the prior odds to the posterior odds for the model ^31^. As a Bayesian inference metric, BF (m) provides a natural ratio to compare the marginal likelihoods between a null and an alternative hypothesis. We used the following categories of evidence for the Bayes factor BF(m) according to the Jeffreys scale: BF(m) between 3-10 (moderate evidence), BF(m) between 10-30 (strong evidence), BF(m) between 30-100 (very strong evidence), and BF(m) >100 (extremely strong evidence) ^32^. Categorical variables are summarized as frequency counts and percentages with 95% confidence intervals (95% CI). Quantitative variables are expressed as the mean and standard deviation (SD) or the median and interquartile range (IQR), as appropriate. In animal experiments, comparisons of iron biochemical parameters between subgroups were performed using Student’s *t-*test or the Mann‒Whitney *U* test, depending on the parametric or nonparametric distribution of the variables, respectively. All bioinformatic analyses were performed using SVS (v8.9.0; Golden Helix, Inc., Bozeman, MT, USA), MedCalc (v20.014; MedCalc Software Ltd., Ostend, Belgium), and JASP (v0.14.1) software ^33^. For the remaining analyses on cellular models, we utilized GraphPad Prism version (v8.0.0) (GraphPad Software, San Diego, California, USA).

# Study design and setting of the replication study

We performed a replication analysis in an independent prospective cohort of patients with unexplained hyperferritinemia recruited between 2014 and 2023 in the Ile-de-France region, France. Patients were recruited from the Gastroenterology departments of Louis Mourier, Beaujon, and Bichat Hospitals (Université Paris Cité) and referred to the Department of Genetics at Bichat University Hospital, a national reference laboratory for heme and iron metabolism certified by the French Ministry of Health. The inclusion criterion was unexplained hyperferritinemia after comprehensive clinical and molecular evaluation. Genetic analyses had previously excluded defects in *HFE* (C282Y homozygous, compound C282Y/H63D heterozygous), *HJV*, *TFR2*, *HAMP*, *SLC40A1*, and *BMP6* genes. Exclusion criteria included hyperferritinemia of other known origin: chronic or acute inflammatory syndrome, history of transfusions, hematological diseases, history of phlebotomy and iron supplementation, cellular lysis, dysmetabolic syndrome, and excessive alcohol consumption. Written informed consent was obtained from all participants. The study was approved by the local ethics committees.

# MUC13 genotyping in the replication study

Genomic DNA was extracted from 200 µL of whole blood using a Chemagic Star DNA extractor (Hamilton, Reno, NV, USA), and DNA quantification was performed using Qubit equipment (Invitrogen, Carlsbad, CA, USA). Patients were initially screened using a custom next-generation sequencing panel targeting genes implicated in iron overload. The sequencing library was generated from 10 ng of genomic DNA using the Ion AmpliSeq Library Kit 2.0 and Ion PGM Template 200 Kit (Thermo Fisher Scientific, Waltham, MA, USA). Sequencing was performed using Ion PGM 200 V2 sequencing kit chemistry on an Ion PGM 316 chip on an Ion Torrent PGM (Thermo Fisher Scientific). Post-run quality control and filtering were performed using Torrent Suite (v3.6). Bioinformatic analyses were conducted using Ion Torrent Browser and Ion Reporter Software (Thermo Fisher Scientific). Sequence data were visually confirmed with the Integrative Genomics Viewer (IGV), and any sequence, alignment, or variant call artifacts were discarded.

In patients without detected variants in *HFE*, *HJV*, *TFR2*, *HAMP*, *SLC40A1*, and *BMP6* genes, genotyping for the *MUC13* p.Arg503Ser variant was performed by Sanger sequencing. Primers were designed as follows: forward, 5'-TCAAGAAACTTTCTGCCATTGTCA-3' (Tm: 59.4°C); reverse, 5'-TGAAGATTCAGCAACCAGGTCT-3' (Tm: 59.6°C). Both strands of PCR products were sequenced using the BigDye Terminator Cycle Sequencing Ready Reaction kit on an ABI PRISM 3130xl sequencer (Applied Biosystems, Foster City, CA, USA).

# Statistical analysis of the replication study

Continuous variables are presented as median (25th–75th percentile), and categorical variables are presented as number (percentage) with 95% confidence intervals. Genotype frequencies were tested for Hardy-Weinberg equilibrium using the chi-square test. Allele and genotype frequencies were compared between the replication cohort and gnomAD reference populations using the chi-square test or Fisher exact test, as appropriate, with calculation of risk ratios and 95% confidence intervals. Multivariable logistic regression was performed to identify independent predictors of elevated transferrin saturation (>55%), with candidate variables including the *MUC13* p.Arg503Ser genotype (recoded as Ser/Ser vs. other genotypes), sex, age, and serum ferritin (>1500 µg/L). Model fit was assessed using the Hosmer-Lemeshow goodness-of-fit test and Nagelkerke R². Receiver operating characteristic curve analysis was performed to evaluate the discriminative ability of the model. Statistical analyses were performed using MedCalc Statistical Software (MedCalc Software Ltd, Ostend, Belgium). A two-sided *P* value of less than 0.05 was considered statistically significant.

# In silico prediction of phosphorylation sites

The full-length reference sequence of human MUC13 (UniProt Q9H3R2, 512 amino acids) and the corresponding p.Arg503Ser sequence were submitted in the same run to NetPhos 3.1^34,35^ and to GPS 6.0^36^. The two sequences differed only at position 503. For NetPhos, generic and kinase-specific predictions were retrieved, and a score above 0.5 defined a positive prediction. For GPS, the medium stringency setting was applied, and predictions were retained when the score exceeded the cutoff returned by the software for the corresponding kinase group. The cutoff was 0.504 for the TKL group, to which the prediction at position 503 belongs.

# Artificial intelligence disclosure

The first draft was written by the authors. No external writing assistance was provided. Claude (Anthropic) was used for quality control during manuscript preparation. The authors take full responsibility for the content.

**SUPPLEMENTARY TABLES**

# Supplementary Table S1. List of the 4733 genes assessed in the exome-based association study

*A2M, A4GALT, A4GNT, AAAS, AADAC, AADACL2, AANAT, AARS, AARS2, AASS, ABAT, ABCA1, ABCA10, ABCA12, ABCA13, ABCA2, ABCA3, ABCA4, ABCA7, ABCB1, ABCB11, ABCB4, ABCB6, ABCB7, ABCC1, ABCC11, ABCC2, ABCC3, ABCC4, ABCC6, ABCC8, ABCC9, ABCD1, ABCD3, ABCD4, ABCG1, ABCG2, ABCG5, ABCG8, ABHD12, ABHD5, ABI3BP, ABO, ACACA, ACACB, ACAD8, ACAD9, ACADL, ACADM, ACADS, ACADSB, ACADVL, ACAN, ACAT1, ACAT2, ACBD5, ACBD6, ACCS, ACE, ACHE, ACLY, ACO2, ACOX1, ACP5, ACSF3, ACSL4, ACSL5, ACSM2B, ACSM3, ACTA1, ACTA2, ACTB, ACTC1, ACTG1, ACTN2, ACTN3, ACTN4, ACVR1, ACVR1B, ACVR1C, ACVR2B, ACVRL1, ACY1, ADA, ADAM10, ADAM12, ADAM17, ADAM19, ADAM33, ADAM7, ADAM9, ADAMTS10, ADAMTS13, ADAMTS16, ADAMTS17, ADAMTS18, ADAMTS2, ADAMTSL2, ADAMTSL3, ADAMTSL4, ADAR, ADARB1, ADCY10, ADCY3, ADCY5, ADCY6, ADCY9, ADCYAP1, ADD1, ADH1B, ADH1C, ADH4, ADH5, ADH7, ADIPOQ, ADIPOR1, ADK, ADM, ADNP, ADORA1, ADORA2A, ADORA3, ADRA1A, ADRA2A, ADRA2B, ADRA2C, ADRB1, ADRB2, ADRB3, ADSL, ADTRP, AFF2, AFF3, AFG3L2, AFP, AGA, AGBL4, AGER, AGGF1, AGK, AGL, AGMO, AGO1, AGO2, AGPAT2, AGPS, AGRN, AGRP, AGT, AGTR1, AGTR2, AGXT, AGXT2, AHCY, AHI1, AHR, AHRR, AHSG, AHSP, AICDA, AIF1, AIFM1, AIMP1, AIP, AIPL1, AIRE, AK1, AK2, AKAP10, AKAP13, AKAP2, AKAP9, AKR1B1, AKR1C2, AKR1C3, AKR1C4, AKR1D1, AKR7A2, AKR7A3, AKT1, AKT2, AKT3, ALAD, ALAS2, ALB, ALCAM, ALDH16A1, ALDH18A1, ALDH1A1, ALDH1A2, ALDH2, ALDH3A2, ALDH4A1, ALDH5A1, ALDH6A1, ALDH7A1, ALDOA, ALDOB, ALG1, ALG10B, ALG11, ALG12, ALG13, ALG2, ALG3, ALG6, ALG8, ALG9, ALK, ALMS1, ALOX12, ALOX12B, ALOX15, ALOX5, ALOX5AP, ALOXE3, ALPL, ALS2, ALS2CL, ALX1, ALX3, ALX4, AMACR, AMELX, AMELY, AMER1, AMH, AMHR2, AMN, AMPD1, AMPD3, AMT, ANAPC1, ANG, ANGPT1, ANGPTL3, ANGPTL4, ANGPTL5, ANK1, ANK2, ANK3, ANKH, ANKK1, ANKRD1, ANKRD11, ANKRD26, ANKS1A, ANKS1B, ANO10, ANO5, ANO6, ANO7, ANTXR1, ANTXR2, ANXA11, ANXA5, AOAH, AOC1, AP1S1, AP1S2, AP3B1, AP3B2, AP4B1, AP4E1, AP4M1, AP4S1, AP5Z1, APAF1, APBA2, APBB1, APBB3, APC, APCDD1, APEX1, APH1A, APH1B, APLNR, APOA1, APOA2, APOA4, APOA5, APOB, APOBEC3B, APOBEC3G, APOBEC3H, APOC1, APOC2, APOC3, APOC4, APOD, APOE, APOH, APOL1, APOL3, APOM, APP, APRT, APTX, AQP1, AQP2, AQP3, AQP4, AQP5, AQP7, AR, AREL1, ARF4, ARFGEF2, ARG1, ARHGAP24, ARHGAP31, ARHGAP6, ARHGAP9, ARHGEF10, ARHGEF11, ARHGEF12, ARHGEF6, ARHGEF7, ARHGEF9, ARID1A, ARID1B, ARL11, ARL13B, ARL14EP, ARL6, ARL6IP5, ARMS2, ARPC3, ARSA, ARSB, ARSE, ARSF, ART4, ARVCF, ARX, AS3MT, ASAH1, ASAH2, ASB10, ASCC1, ASCC3, ASCL1, ASIC3, ASIP, ASL, ASMT, ASMTL, ASNS, ASPA, ASPM, ASPN, ASPRV1, ASS1, ASTN2, ASXL1, ATCAY, ATF1, ATF3, ATF5, ATF6, ATG16L1, ATG7, ATIC, ATL1, ATM, ATN1, ATOH7, ATP10A, ATP10D, ATP13A2, ATP13A4, ATP1A2, ATP1A3, ATP1B1, ATP2A1, ATP2A2, ATP2A3, ATP2B2, ATP2B4, ATP2C1, ATP5E, ATP6AP2, ATP6V0A1, ATP6V0A2, ATP6V0A4, ATP6V1B1, ATP7A, ATP7B, ATP8A2, ATP8B1, ATPAF2, ATR, ATRNL1, ATRX, ATXN1, ATXN10, ATXN2, ATXN3, ATXN3L, ATXN7, ATXN8OS, AUH, AURKA, AURKC, AUTS2, AVP, AVPR1A, AVPR1B, AVPR2, AXIN1, AXIN2, AXL, B2M, B3GALNT1, B3GALT6, B3GAT3, B3GNT3, B4GALT1, B4GALT7, B9D1, B9D2, BAALC, BAAT, BACE1, BAG3, BAG6, BANF1, BANK1, BAP1, BARD1, BAX, BBS1, BBS10, BBS12, BBS2, BBS4, BBS5, BBS7, BBS9, BCAM, BCAP31, BCAT1, BCAT2, BCHE, BCKDHA, BCKDHB, BCL10, BCL11A, BCL2, BCL2A1, BCL2L11, BCL2L2, BCL9, BCOR, BCORL1, BCR, BCS1L, BDKRB2, BDNF, BEAN1, BEST1, BEX4, BFSP1, BFSP2, BHLHA9, BHLHE41, BHMT, BICC1, BICD1, BIN1, BIRC5, BLK, BLM, BLMH, BLNK, BLOC1S3, BLOC1S6, BLVRA, BMI1, BMP1, BMP10, BMP15, BMP2, BMP2K, BMP4, BMP5, BMP7, BMPER, BMPR1A, BMPR1B, BMPR2, BNC2, BOLA3, BPGM, BPI, BPIFA1, BPY2, BRAF, BRAP, BRAT1, BRCA1, BRCA2, BRCC3, BRD1, BRIP1, BRSK2, BRWD1, BRWD3, BSCL2, BSG, BSND, BST1, BTAF1, BTBD9, BTC, BTD, BTK, BTLA, BTN1A1, BTN2A1, BTNL2, BTRC, BUB1B, C12orf10, C12orf57, C12orf65, C19orf12, C1GALT1, C1QA, C1QB, C1QC, C1QTNF5, C1R, C1S, C2, C2orf71, C3, C3AR1, C4A, C4B, C4BPA, C5, C5AR2, C5orf42, C6, C6orf15, C7, C8A, C8B, C8orf37, C9, C9orf72, CA1, CA12, CA2, CA4, CA6, CA8, CABIN1, CABP4, CACNA1A, CACNA1C, CACNA1D, CACNA1E, CACNA1F, CACNA1G, CACNA1H, CACNA1S, CACNA2D1, CACNA2D3, CACNA2D4, CACNB2, CACNB4, CACNG2, CADM1, CALCA, CALCR, CALHM1, CALM1, CALM3, CALR, CALR3, CAMK4, CAMKK1, CAMKK2, CAMP, CAMTA1, CANT1, CAPN10, CAPN13, CAPN3, CARD14, CARD8, CARD9, CARTPT, CASC16, CASK, CASP1, CASP10, CASP12, CASP2, CASP3, CASP5, CASP8, CASP9, CASQ2, CASR, CAST, CAT, CATSPER1, CATSPER2, CATSPER3, CATSPER4, CAV1, CAV3, CBFB, CBL, CBLB, CBR1, CBR3, CBS, CBX2, CBX4, CC2D1A, CC2D2A, CCBE1, CCDC103, CCDC14, CCDC170, CCDC22, CCDC28B, CCDC39, CCDC40, CCDC50, CCDC8, CCDC88C, CCHCR1, CCK, CCKAR, CCKBR, CCL11, CCL13, CCL17, CCL2, CCL22, CCL26, CCL3L1, CCL4L1, CCL5, CCL7, CCM2, CCNA2, CCND1, CCPG1, CCR1, CCR2, CCR3, CCR5, CCR6, CCR7, CCRL2, CCT5, CD109, CD14, CD151, CD177, CD19, CD1A, CD1E, CD207, CD209, CD22, CD226, CD24, CD244, CD247, CD27, CD2AP, CD320, CD36, CD38, CD3D, CD3E, CD3EAP, CD3G, CD4, CD40, CD40LG, CD44, CD46, CD5, CD55, CD58, CD59, CD72, CD79A, CD79B, CD81, CD86, CD8A, CD96, CDA, CDAN1, CDC42BPB, CDC6, CDC73, CDH1, CDH12, CDH13, CDH15, CDH23, CDH3, CDH5, CDH8, CDHR1, CDK11A, CDK4, CDK5R1, CDK5RAP2, CDK5RAP3, CDK7, CDKL3, CDKL5, CDKN1A, CDKN1B, CDKN1C, CDKN2A, CDKN2B, CDKN2B-AS1, CDKN2C, CDON, CDSN, CDT1, CDY1, CDY2A, CEACAM16, CEBPA, CEBPE, CEL, CELSR1, CENPJ, CEP135, CEP152, CEP290, CEP41, CEP57, CEP63, CEP68, CEP85L, CER1, CERKL, CERS6, CES1, CES2, CETP, CFB, CFC1, CFD, CFH, CFHR1, CFHR2, CFHR3, CFHR4, CFHR5, CFI, CFL2, CFLAR, CFP, CFTR, CGA, CHAT, CHD1L, CHD2, CHD3, CHD6, CHD7, CHD8, CHDH, CHEK2, CHFR, CHGA, CHGB, CHI3L1, CHI3L2, CHIA, CHIT1, CHKB, CHL1, CHM, CHMP2B, CHMP4B, CHN1, CHRDL1, CHRFAM7A, CHRM1, CHRM2, CHRM3, CHRNA1, CHRNA2, CHRNA3, CHRNA4, CHRNA5, CHRNA7, CHRNA9, CHRNB1, CHRNB2, CHRNB4, CHRND, CHRNE, CHRNG, CHST14, CHST3, CHST6, CHST8, CHSY1, CHUK, CIB2, CIC, CIDEA, CIDEC, CIITA, CILP, CISD2, CISH, CITED2, CKM, CLCA1, CLCA2, CLCF1, CLCN1, CLCN2, CLCN5, CLCN7, CLCNKA, CLCNKB, CLDN1, CLDN14, CLDN16, CLDN19, CLEC11A, CLEC2D, CLEC4M, CLEC7A, CLIC2, CLK2, CLMP, CLN3, CLN5, CLN6, CLN8, CLNK, CLOCK, CLPS, CLPTM1, CLRN1, CLSTN2, CLTCL1, CLU, CLUL1, CLYBL, CMA1, CMPK1, CNBP, CNDP1, CNGA1, CNGA3, CNGB1, CNGB3, CNKSR1, CNKSR2, CNNM2, CNNM4, CNOT3, CNOT4, CNPY3, CNR1, CNR2, CNTF, CNTN1, CNTN4, CNTNAP2, CNTNAP4, CNTNAP5, COA5, COCH, COG1, COG4, COG5, COG6, COG7, COG8, COL10A1, COL11A1, COL11A2, COL12A1, COL17A1, COL18A1, COL1A1, COL1A2, COL25A1, COL2A1, COL3A1, COL4A1, COL4A2, COL4A3, COL4A4, COL4A5, COL4A6, COL5A1, COL5A2, COL6A1, COL6A2, COL6A3, COL6A4P2, COL6A5, COL7A1, COL8A2, COL9A1, COL9A2, COL9A3, COLEC11, COLQ, COMMD1, COMP, COMT, COQ2, COQ4, COQ5, COQ6, COQ9, CORIN, CORO1A, COX10, COX14, COX15, COX4I1, COX4I2, COX6B1, COX7A1, COX7A2, CP, CPA4, CPA6, CPB2, CPE, CPLX2, CPN1, CPOX, CPS1, CPT1A, CPT1B, CPT2, CPZ, CR1, CR2, CRADD, CRB1, CRBN, CREB1, CREB3L3, CREBBP, CRELD1, CRH, CRHR1, CRISP2, CRK, CRKL, CRLF1, CRP, CRTAP, CRX, CRYAA, CRYAB, CRYBA1, CRYBA4, CRYBB1, CRYBB2, CRYBB3, CRYGB, CRYGC, CRYGD, CRYGEP, CRYGS, CRYM, CSAG1, CSDE1, CSF1, CSF1R, CSF2, CSF2RA, CSF2RB, CSF3R, CSGALNACT1, CSH1, CSMD1, CSMD3, CSNK1A1L, CSNK1D, CSNK1E, CSNK2A2, CSNK2A3, CSRP3, CST3, CSTA, CSTB, CSTF2T, CTC1, CTDP1, CTF1, CTGF, CTH, CTHRC1, CTLA4, CTNNA3, CTNNB1, CTNND1, CTNS, CTRC, CTSA, CTSB, CTSC, CTSD, CTSG, CTSK, CTSZ, CTTNBP2, CUBN, CUL3, CUL4B, CUL5, CUL7, CX3CR1, CXCL10, CXCL11, CXCL12, CXCL16, CXCL5, CXCR1, CXCR3, CXCR4, CYB5A, CYB5R3, CYBA, CYBB, CYBRD1, CYCS, CYLD, CYP11A1, CYP11B1, CYP11B2, CYP17A1, CYP19A1, CYP1A1, CYP1A2, CYP1B1, CYP21A2, CYP24A1, CYP26A1, CYP26B1, CYP27A1, CYP27B1, CYP2A13, CYP2A6, CYP2B6, CYP2C18, CYP2C19, CYP2C8, CYP2C9, CYP2D6, CYP2E1, CYP2F1, CYP2G1P, CYP2J2, CYP2R1, CYP2W1, CYP3A4, CYP3A43, CYP3A5, CYP3A7, CYP46A1, CYP4A11, CYP4A22, CYP4B1, CYP4F12, CYP4F2, CYP4F22, CYP4F3, CYP4V2, CYP7A1, CYP7B1, CYSLTR1, CYSLTR2, D2HGDH, DAG1, DAO, DAOA, DAPK1, DARS2, DAZ1, DAZ2, DAZ3, DAZL, DBH, DBI, DBT, DCAF13, DCAF17, DCC, DCDC2, DCK, DCLK1, DCLRE1C, DCN, DCP1B, DCTD, DCTN1, DCX, DCXR, DDAH1, DDAH2, DDB2, DDC, DDOST, DDR1, DDR2, DDX11, DDX20, DDX25, DDX39B, DDX3Y, DDX5, DDX53, DDX58, DEAF1, DEC1, DECR1, DEFB1, DEFB126, DEFB4A, DES, DGAT1, DGCR2, DGCR5, DGCR6, DGCR8, DGKD, DGUOK, DHCR24, DHCR7, DHDDS, DHFR, DHH, DHODH, DHRS4L1, DHX16, DHX36, DHX37, DIABLO, DIAPH1, DIAPH2, DIAPH3, DICER1, DIO1, DIO2, DIP2A, DIP2B, DIP2C, DIRC2, DIS3L2, DISC1, DISP1, DKC1, DKK2, DKK3, DLAT, DLD, DLG3, DLG5, DLGAP2, DLGAP3, DLL1, DLL3, DLX3, DLX5, DLX6, DMBT1, DMC1, DMD, DMGDH, DMP1, DMPK, DMRT1, DMXL1, DNAAF1, DNAAF2, DNAAF3, DNAH11, DNAH5, DNAH9, DNAI1, DNAI2, DNAJA4, DNAJB2, DNAJB6, DNAJC19, DNAJC5, DNAJC6, DNAL1, DNASE1, DNASE1L3, DNASE2, DND1, DNM1, DNM1L, DNM2, DNMT1, DNMT3A, DNMT3B, DNMT3L, DOC2A, DOCK3, DOCK4, DOCK6, DOCK8, DOCK9, DOK7, DOLK, DPAGT1, DPM1, DPM3, DPP10, DPP6, DPY19L2, DPYD, DPYS, DPYSL2, DRD1, DRD2, DRD3, DRD4, DRD5, DROSHA, DRP2, DSC2, DSC3, DSCAM, DSCR8, DSG1, DSG2, DSG4, DSP, DSPP, DST, DTNA, DTNBP1, DUOX2, DUOXA2, DUSP23, DUX4, DXO, DYM, DYNC1H1, DYNC2H1, DYRK1A, DYSF, EARS2, EBAG9, EBP, ECE1, ECI1, ECM1, ECM2, ECSIT, EDA, EDA2R, EDAR, EDARADD, EDN1, EDN2, EDN3, EDNRA, EDNRB, EEF1B2, EEF2K, EFCAB5, EFEMP1, EFEMP2, EFHC1, EFHC2, EFNB1, EFR3A, EFTUD2, EGF, EGFR, EGLN1, EGR2, EGR3, EHD2, EHMT1, EHMT2, EIF2AK3, EIF2B1, EIF2B2, EIF2B3, EIF2B4, EIF2B5, EIF3H, EIF4E, EIF4G1, ELAC2, ELANE, ELAVL2, ELF4, ELK1, ELN, ELOVL4, ELP2, ELP4, EMC4, EMD, EME1, EMG1, EMX2, EN2, ENAM, ENG, ENO1, ENO3, ENPP1, ENSA, ENTPD1, EOMES, EP300, EPAS1, EPB41, EPB41L1, EPB42, EPC2, EPCAM, EPHA2, EPHA3, EPHA5, EPHA7, EPHB2, EPHB6, EPHX1, EPHX2, EPM2A, EPO, EPOR, EPX, ERAP1, ERAP2, ERBB2, ERBB3, ERBB4, ERCC1, ERCC2, ERCC3, ERCC4, ERCC5, ERCC6, ERCC8, ERLIN2, ERMAP, ESAM, ESCO2, ESPN, ESR1, ESR2, ESRRB, ESRRG, ETFA, ETFB, ETFDH, ETHE1, ETNPPL, ETS1, EVC, EVC2, EVI5, EXO1, EXO5, EXOC4, EXOSC3, EXT1, EXT2, EXTL3, EYA1, EYA4, EYS, EZH2, F10, F11, F12, F13A1, F13B, F2, F2R, F2RL1, F3, F5, F7, F8, F9, FA2H, FAAH, FAAH2, FABP1, FABP2, FABP3, FABP4, FABP6, FABP7, FADD, FADS2, FAH, FAM120A, FAM126A, FAM161A, FAM205A, FAM20A, FAM20C, FAM83H, FAM8A1, FAM91A1, FANCA, FANCB, FANCC, FANCD2, FANCE, FANCF, FANCG, FANCI, FANCL, FANCM, FARS2, FAS, FASLG, FASN, FASTKD2, FBLIM1, FBLN1, FBLN5, FBN1, FBN2, FBN3, FBP1, FBXL6, FBXO10, FBXO18, FBXO7, FBXW11, FBXW4, FBXW7, FCAR, FCER1A, FCER2, FCGR1A, FCGR2A, FCGR2B, FCGR3A, FCGR3B, FCGRT, FCN2, FCN3, FCRL3, FDFT1, FECH, FEM1A, FEN1, FERMT1, FERMT3, FEV, FEZF2, FFAR1, FFAR4, FGA, FGB, FGD1, FGD3, FGD4, FGF1, FGF10, FGF14, FGF2, FGF20, FGF23, FGF3, FGF8, FGF9, FGFBP1, FGFR1, FGFR2, FGFR3, FGFR4, FGFRL1, FGG, FH, FHL1, FHL2, FIG4, FIGLA, FIP1L1, FKBP10, FKBP14, FKBP5, FKBP6, FKBPL, FKRP, FKTN, FLCN, FLG, FLNA, FLNB, FLNC, FLT1, FLT3, FLT4, FLVCR1, FLVCR2, FMN1, FMN2, FMO1, FMO2, FMO3, FMO4, FMO5, FMO6P, FMR1, FN1, FN3K, FOLH1, FOLR1, FOXA1, FOXA2, FOXA3, FOXC1, FOXC2, FOXD3, FOXE1, FOXE3, FOXF1, FOXF2, FOXG1, FOXH1, FOXI1, FOXL2, FOXN1, FOXP1, FOXP2, FOXP3, FOXRED1, FPGS, FPR1, FPR2, FRA10AC1, FRAS1, FREM1, FREM2, FREM3, FRG1, FRK, FRMD6, FRMD7, FRMPD4, FRY, FRZB, FSCB, FSCN2, FSHB, FSHR, FTCD, FTH1, FTHL17, FTL, FTO, FTSJ1, FUCA1, FURIN, FUS, FUT1, FUT2, FUT3, FUT6, FUT7, FUT8, FUZ, FXN, FXYD2, FXYD6, FYCO1, FZD1, FZD3, FZD4, FZD6, FZD9, G6PC, G6PC2, G6PC3, G6PD, GAA, GAB2, GABBR1, GABRA1, GABRA5, GABRA6, GABRB3, GABRD, GABRG1, GABRG2, GABRG3, GABRR2, GAD1, GAD2, GADD45B, GAK, GAL3ST3, GALC, GALE, GALK1, GALNS, GALNT12, GALNT18, GALNT2, GALNT3, GALNT9, GALNTL5, GALP, GALT, GAMT, GAN, GAP43, GARS, GAS1, GAS2L2, GAS6, GATA1, GATA2, GATA3, GATA4, GATA6, GATAD1, GATM, GBA, GBA3, GBE1, GBGT1, GC, GCDH, GCGR, GCH1, GCK, GCKR, GCLC, GCLM, GCM2, GCNT2, GCSH, GDAP1, GDF1, GDF15, GDF3, GDF5, GDF6, GDF9, GDI1, GDNF, GEMIN2, GEMIN4, GFAP, GFER, GFI1, GFI1B, GFM1, GFPT1, GFPT2, GFRA1, GGCX, GGH, GGT5, GH1, GH2, GHR, GHRH, GHRHR, GHRL, GHSR, GIF, GIGYF2, GIMAP8, GIP, GIPC3, GIPR, GIT1, GJA1, GJA3, GJA4, GJA5, GJA8, GJB1, GJB2, GJB3, GJB4, GJB6, GJC2, GJC3, GJD2, GK, GLA, GLB1, GLCCI1, GLDC, GLE1, GLI1, GLI2, GLI3, GLIS2, GLIS3, GLMN, GLO1, GLP1R, GLRA1, GLRB, GLRX5, GLS, GLUD1, GLUD2, GLUL, GLYCTK, GM2A, GMIP, GNA14, GNAI2, GNAQ, GNAS, GNAS-AS1, GNAT1, GNAT2, GNB1L, GNB3, GNB5, GNE, GNMT, GNPAT, GNPTAB, GNPTG, GNRH1, GNRHR, GNS, GOLGA3, GOLGA5, GON4L, GORAB, GOSR2, GOT1, GP1BA, GP1BB, GP2, GP6, GP9, GPAM, GPANK1, GPATCH8, GPBAR1, GPC3, GPC4, GPC6, GPD1, GPD1L, GPD2, GPHN, GPI, GPIHBP1, GPR1, GPR12, GPR139, GPR143, GPR179, GPR33, GPR55, GPR68, GPS1, GPSM2, GPT, GPX1, GPX4, GRB10, GREM1, GRHL2, GRHPR, GRIA3, GRID1, GRIK1, GRIK2, GRIK3, GRIK4, GRIN1, GRIN2A, GRIN2B, GRIN3A, GRIP1, GRK1, GRK4, GRK5, GRM1, GRM3, GRM5, GRM6, GRM7, GRM8, GRN, GRPR, GRXCR1, GSC, GSDMA, GSDMB, GSE1, GSK3B, GSN, GSPT1, GSPT2, GSR, GSS, GSTA1, GSTA2, GSTA3, GSTK1, GSTM1, GSTM3, GSTM4, GSTO1, GSTO2, GSTP1, GSTT1, GSTT2, GSTT2B, GSTZ1, GTF2E1, GTF2H1, GTF2H5, GTF2IRD1, GTF2IRD2, GUCA1A, GUCA1B, GUCY2C, GUCY2D, GUSB, GYG1, GYPA, GYPB, GYPC, GYPE, GYS1, GYS2, GZMB, H19, H2BFWT, H6PD, HABP2, HACE1, HADH, HADHA, HADHB, HAL, HAMP, HAND1, HAND2, HARS, HARS2, HAS1, HAVCR1, HAX1, HBA1, HBA2, HBB, HBD, HBE1, HBEGF, HBG1, HBG2, HBM, HBS1L, HBZ, HCCS, HCK, HCLS1, HCN1, HCN2, HCN4, HCP5, HCRT, HCRTR1, HCRTR2, HDAC4, HDAC8, HDAC9, HDC, HDLBP, HDX, HELQ, HEPACAM, HERC2, HES6, HES7, HESX1, HEXA, HEXB, HEY1, HFE, HFE2, HGD, HGF, HGSNAT, HHEX, HHIP, HIBCH, HIF1A, HIF1AN, HIGD2A, HIP1, HIST1H2AE, HIST3H3, HK1, HK2, HLA-A, HLA-B, HLA-C, HLA-DMB, HLA-DOA, HLA-DPB1, HLA-DPB2, HLA-DQA1, HLA-DQB1, HLA-DRA, HLA-DRB1, HLA-DRB5, HLA-E, HLA-G, HLCS, HLX, HMBS, HMCN1, HMGA1, HMGA2, HMGCL, HMGCR, HMGCS2, HMOX1, HMOX2, HMSD, HMX1, HMX2, HNF1A, HNF1B, HNF4A, HNMT, HNRNPH3, HNRNPU, HOGA1, HOMER2, HOXA1, HOXA10, HOXA11, HOXA13, HOXA2, HOXA4, HOXB13, HOXB6, HOXD10, HOXD13, HOXD4, HP, HPD, HPGD, HPRT1, HPS1, HPS3, HPS4, HPS5, HPS6, HPSE2, HR, HRAS, HRC, HRG, HRH2, HRH3, HS1BP3, HS6ST1, HSD11B1, HSD11B2, HSD17B1, HSD17B10, HSD17B2, HSD17B3, HSD17B4, HSD3B1, HSD3B2, HSD3B7, HSF4, HSFY1, HSP90AA1, HSP90B1, HSPA1A, HSPA1B, HSPA1L, HSPA5, HSPA8, HSPA9, HSPB1, HSPB3, HSPB6, HSPB7, HSPB8, HSPD1, HSPG2, HTN3, HTR1A, HTR1B, HTR2A, HTR2B, HTR2C, HTR3A, HTR3B, HTR3C, HTR3E, HTR5A, HTR6, HTR7, HTRA1, HTRA2, HTT, HUS1B, HUWE1, HVCN1, HYAL1, HYDIN, HYLS1, HYMAI, IAPP, IBSP, ICAM1, ICAM4, ICAM5, ICK, ICOS, ID3, IDE, IDH1, IDH2, IDH3B, IDO1, IDS, IDUA, IER3IP1, IFI30, IFI44L, IFIH1, IFITM3, IFITM5, IFNA10, IFNA17, IFNA2, IFNAR1, IFNAR2, IFNG, IFNGR1, IFNGR2, IFNL3, IFRD1, IFT122, IFT140, IFT43, IFT80, IGBP1, IGF1, IGF1R, IGF2, IGF2R, IGFALS, IGFBP1, IGFBP3, IGFBP5, IGFBP7, IGHA1, IGHG2, IGHM, IGHMBP2, IGLL1, IHH, IKBIP, IKBKG, IL10, IL10RA, IL10RB, IL11RA, IL12A, IL12B, IL12RB1, IL12RB2, IL13, IL16, IL17A, IL17F, IL17RA, IL17RB, IL17REL, IL18, IL18R1, IL18RAP, IL19, IL1A, IL1B, IL1R1, IL1RAPL1, IL1RL1, IL1RN, IL2, IL20RA, IL20RB, IL21, IL21R, IL23R, IL2RA, IL2RG, IL3, IL31RA, IL36RN, IL4, IL4R, IL5, IL6, IL6R, IL7, IL7R, IL9, IL9R, ILDR1, ILK, IMMP2L, IMMT, IMPA2, IMPAD1, IMPDH1, IMPDH2, IMPG2, INF2, INHA, INMT, INPP4A, INS, INSIG1, INSIG2, INSL3, INSL6, INSR, INVS, IQCB1, IQGAP1, IQGAP2, IQGAP3, IQSEC2, IRAK1, IRAK3, IRAK4, IRF1, IRF2, IRF4, IRF5, IRF6, IRF7, IRF8, IRGM, IRS1, IRS2, IRS4, IRX4, IRX5, ISCU, ISL1, ISPD, ISYNA1, ITCH, ITGA11, ITGA2, ITGA2B, ITGA3, ITGA4, ITGA6, ITGA7, ITGA9, ITGAE, ITGAM, ITGB2, ITGB3, ITGB4, ITIH1, ITIH3, ITIH4, ITIH6, ITK, ITM2B, ITPA, ITPKC, ITPR1, ITPR3, ITSN2, IVD, IYD, JAG1, JAG2, JAK2, JAK3, JAM3, JMJD1C, JPH2, JPH3, JRK, JUN, JUP, KALRN, KANK1, KANSL1, KARS, KAT6B, KATNAL2, KBTBD13, KCNA1, KCNA3, KCNA5, KCNAB2, KCNC3, KCND2, KCND3, KCNE1, KCNE2, KCNE3, KCNE4, KCNH2, KCNIP4, KCNJ1, KCNJ10, KCNJ11, KCNJ13, KCNJ15, KCNJ2, KCNJ3, KCNJ5, KCNJ6, KCNJ8, KCNJ9, KCNK18, KCNK6, KCNK9, KCNMA1, KCNMB1, KCNMB3, KCNN3, KCNQ1, KCNQ2, KCNQ3, KCNQ4, KCNS1, KCNS3, KCNV2, KCTD13, KCTD7, KDM4C, KDM5A, KDM5C, KDM5D, KDM6A, KDM6B, KDR, KEL, KERA, KHDC3L, KHK, KIAA0100, KIAA0232, KIAA0319, KIAA0513, KIF11, KIF17, KIF18A, KIF1A, KIF1B, KIF21A, KIF22, KIF5A, KIF5B, KIF6, KIF7, KIFAP3, KIR2DL1, KIR2DL3, KIR2DL4, KIR3DL1, KIR3DL2, KIRREL3, KISS1, KISS1R, KIT, KITLG, KL, KLB, KLF1, KLF10, KLF11, KLF5, KLF6, KLF7, KLHDC8B, KLHL10, KLHL3, KLHL7, KLHL9, KLK1, KLK12, KLK15, KLK3, KLK4, KLK7, KLKB1, KLRK1, KMT2C, KMT2D, KMT2E, KNG1, KPNA1, KRAS, KRIT1, KRT1, KRT10, KRT12, KRT13, KRT14, KRT16, KRT17, KRT18, KRT2, KRT3, KRT31, KRT37, KRT38, KRT4, KRT5, KRT6A, KRT6B, KRT6C, KRT74, KRT75, KRT8, KRT81, KRT83, KRT85, KRT86, KRT9, KRTAP1-1, KYNU, L1CAM, L2HGDH, L3MBTL1, LAMA1, LAMA2, LAMA3, LAMA4, LAMA5, LAMB1, LAMB2, LAMB3, LAMC1, LAMC2, LAMC3, LAMP2, LAMTOR2, LARS2, LBP, LBR, LCA5, LCAT, LCE3B, LCE3C, LCE5A, LCN10, LCT, LDB3, LDHA, LDHB, LDLR, LDLRAD4, LDLRAP1, LEFTY2, LEMD3, LEP, LEPR, LETM1, LFNG, LGALS13, LGALS2, LGALS3, LGI1, LHB, LHCGR, LHFPL5, LHX1, LHX3, LHX4, LHX8, LIAS, LIF, LIFR, LIG1, LIG3, LIG4, LILRA3, LIM2, LIMK1, LIN28A, LIN28B, LIPA, LIPC, LIPE, LIPG, LIPH, LIPI, LIPN, LITAF, LLGL1, LMAN1, LMBR1, LMBRD1, LMF1, LMNA, LMNB1, LMNB2, LMTK3, LMX1B, LNX2, loc344967, LOR, LOX, LOXHD1, LOXL1, LOXL2, LPA, LPAR1, LPAR6, LPIN1, LPIN2, LPIN3, LPL, LPP, LRAT, LRCH1, LRFN5, LRP1, LRP2, LRP4, LRP5, LRP6, LRP8, LRPAP1, LRPPRC, LRRC4, LRRC6, LRRC8A, LRRK2, LRSAM1, LRTOMT, LTA, LTBP1, LTBP2, LTBP3, LTBP4, LTC4S, LTF, LTK, LTN1, LUM, LY96, LYN, LYST, LYZ, LZTFL1, LZTS1, MACROD2, MAD1L1, MAD2L1, MADD, MAF, MAFB, MAGEE2, MAGEL2, MAGI2, MAGT1, MAK, MAMLD1, MAN1A2, MAN1B1, MAN2B1, MANBA, MAOA, MAOB, MAP2, MAP2K1, MAP2K2, MAP2K3, MAP2K4, MAP3K1, MAP3K15, MAP4K5, MAP6, MAP7D3, MAPK10, MAPK8IP1, MAPT, MARS2, MARVELD2, MASP1, MASP2, MAST4, MASTL, MAT1A, MATN3, MATR3, MAVS, MAX, MBD1, MBD3, MBD4, MBD5, MBL2, MBTPS2, MC1R, MC2R, MC3R, MC4R, MCCC1, MCCC2, MCEE, MCF2L2, MCFD2, MCHR1, MCL1, MCM3AP, MCM4, MCM5, MCM6, MCOLN1, MCPH1, MDH1, MDM2, MDM4, MDN1, MECP2, MED12, MED13, MED13L, MED17, MED23, MED25, MEF2A, MEF2C, MEFV, MEGF10, MEGF11, MEIS1, MEIS2, MEN1, MEP1B, MERTK, MESP2, MEST, MET, MFF, MFGE8, MFN2, MFRP, MFSD2A, MFSD8, MGAT1, MGAT2, MGAT4C, MGEA5, MGLL, MGMT, MGP, MGST2, MGST3, MIA3, MIAT, MICA, MICB, MID1, MIF, MIIP, MINPP1, MIP, MIPOL1, MIR106B, MIR124-1, MIR125A, MIR126, MIR140, MIR146A, MIR16-1, MIR17, MIR17HG, MIR182, MIR184, MIR191, MIR196A2, MIR206, MIR24-1, MIR27A, MIR2861, MIR30C1, MIR34B, MIR499A, MIR502, MIR510, MIR890, MIR892B, MIR934, MIR96, MIRLET7E, MITF, MKKS, MKL1, MKRN3, MKS1, MLC1, MLH1, MLH3, MLLT3, MLPH, MLXIPL, MLYCD, MMAA, MMAB, MMACHC, MMADHC, MME, MMEL1, MMP1, MMP10, MMP12, MMP13, MMP14, MMP2, MMP20, MMP3, MMP7, MMP8, MMP9, MNX1, MOCOS, MOCS1, MOCS2, MOG, MOGS, MOK, MPDU1, MPDZ, MPG, MPHOSPH8, MPI, MPL, MPLKIP, MPO, MPP3, MPP4, MPP6, MPP7, MPST, MPV17, MPZ, MR1, MRAP, MRC1, MRE11A, MRPL3, MRPL48, MRPS16, MRPS22, MRRF, MS4A1, MS4A12, MS4A2, MS4A3, MS4A6A, MS4A6E, MSH2, MSH3, MSH4, MSH5, MSH6, MSMB, MSMO1, MSR1, MSRB3, MST1, MST1R, MSTN, MSX1, MSX2, MT1A, MT2A, MTAP, MTCH2, MTFMT, MTHFD1, MTHFD1L, MTHFR, MTHFS, MTM1, MTMR14, MTMR2, MTMR9, MTNR1A, MTNR1B, MTO1, MTPAP, MTR, MTRR, MTTP, MTUS1, MUC1, MUC13, MUC2, MUC3A, MUC4, MUC5B, MUC6, MUC7, MUSK, MUT, MUTYH, MVK, MX1, MYB, MYBL2, MYBPC1, MYBPC3, MYC, MYCL, MYCN, MYD88, MYF6, MYH11, MYH13, MYH14, MYH15, MYH2, MYH3, MYH6, MYH7, MYH8, MYH9, MYL2, MYL3, MYLIP, MYLK, MYLK2, MYO15A, MYO18B, MYO1A, MYO1C, MYO1E, MYO1F, MYO3A, MYO5A, MYO5B, MYO6, MYO7A, MYO7B, MYO9B, MYOC, MYOCD, MYOM1, MYOT, MYOZ2, MYPN, MYT1, MYT1L, NAA10, NAGA, NAGLU, NAGPA, NAGS, NAIP, NAMPT, NARS2, NAT1, NAT2, NAT8L, NAV2, NBAS, NBEA, NBEAL2, NBN, NBPF1, NCALD, NCAM1, NCAN, NCAPD2, NCF1, NCF2, NCF4, NCKAP1, NCOA1, NCOA3, NCR3, NCS1, NCSTN, NDE1, NDN, NDOR1, NDP, NDRG1, NDST1, NDUFA1, NDUFA10, NDUFA11, NDUFA12, NDUFA13, NDUFA2, NDUFA4, NDUFA6, NDUFA7, NDUFA8, NDUFA9, NDUFAF1, NDUFAF2, NDUFAF3, NDUFAF4, NDUFAF5, NDUFAF6, NDUFAF7, NDUFB1, NDUFB3, NDUFB6, NDUFB9, NDUFC2, NDUFS1, NDUFS2, NDUFS3, NDUFS4, NDUFS5, NDUFS6, NDUFS7, NDUFS8, NDUFV1, NDUFV2, NDUFV3, NEB, NEBL, NEDD4L, NEDD9, NEFH, NEFL, NEFM, NEGR1, NEIL1, NEIL2, NEK1, NEK8, NELFA, NELL1, NEU1, NEU2, NEUROD1, NEUROG3, NEXN, NF1, NF2, NFATC4, NFE2L2, NFIA, NFIX, NFKB1, NFKBIA, NFKBIL1, NFU1, NGF, NGFR, NHEJ1, NHLRC1, NHP2, NHS, NID1, NINJ1, NIPA1, NIPAL4, NIPBL, NIPSNAP1, NIPSNAP3A, NKAIN2, NKX2-1, NKX2-3, NKX2-5, NKX2-6, NKX3-1, NKX3-2, NLGN1, NLGN2, NLGN3, NLGN4X, NLGN4Y, NLRP1, NLRP12, NLRP14, NLRP2, NLRP3, NLRP7, NLRX1, NMB, NME1, NME8, NMNAT1, NMT2, NMU, NOBOX, NOD1, NOD2, NODAL, NOG, NOP10, NOP56, NOS1, NOS1AP, NOS2, NOS3, NOTCH1, NOTCH2, NOTCH3, NOTCH4, NPAP1, NPAS2, NPAS3, NPAT, NPC1, NPC1L1, NPC2, NPFFR2, NPHP1, NPHP3, NPHP4, NPHS1, NPHS2, NPL, NPM1, NPPA, NPPB, NPPC, NPR1, NPR2, NPR3, NPSR1, NPTN, NPY, NPY1R, NPY2R, NQO1, NQO2, NR0B1, NR0B2, NR1H2, NR1H3, NR1H4, NR1I2, NR1I3, NR2E1, NR2E3, NR2F1, NR3C1, NR3C2, NR4A2, NR5A1, NRAS, NRG1, NRG3, NRIP1, NRL, NRP2, NRTN, NRXN1, NRXN2, NRXN3, NSD1, NSDHL, NSMF, NSUN2, NSUN7, NT5C1B, NT5C3A, NT5E, NTF3, NTF4, NTHL1, NTNG1, NTRK1, NTRK2, NTRK3, NUAK1, NUBPL, NUDC, NUDT1, NUDT6, NUMBL, NUP155, NUP62, NXF3, NXF5, NXNL1, NYX, OAS1, OAS2, OAT, OAZ1, OBSCN, OBSL1, OCA2, OCLN, OCRL, ODC1, OFD1, OGG1, OLFM2, OLR1, OMG, OPA1, OPA3, OPCML, OPHN1, OPN1LW, OPN1MW, OPN1SW, OPN4, OPRD1, OPRK1, OPRL1, OPRM1, OPTC, OPTN, OR13G1, OR7D4, ORAI1, ORC1, ORC4, ORC6, ORMDL3, OSMR, OSR1, OSTM1, OTC, OTOA, OTOF, OTOR, OTX2, OVGP1, OXCT1, OXTR, P2RX1, P2RX4, P2RX5, P2RX7, P2RY11, P2RY12, P2RY4, PABPC4L, PABPN1, PACRG, PAFAH1B1, PAFAH1B3, PAH, PAK3, PALB2, PALLD, PANK2, PAPD7, PAPSS2, PARD3B, PARD6A, PARK7, PARL, PARP1, PARP2, PASK, PAWR, PAX1, PAX2, PAX3, PAX4, PAX5, PAX6, PAX7, PAX8, PAX9, PC, PCBD1, PCCA, PCCB, PCDH11X, PCDH15, PCDH18, PCDH19, PCDH9, PCDHA10, PCDHA13, PCDHB4, PCK1, PCK2, PCM1, PCMT1, PCNT, PCOLCE, PCSK1, PCSK2, PCSK9, PDCD1, PDCD10, PDCD5, PDE10A, PDE11A, PDE4B, PDE4D, PDE6A, PDE6B, PDE6C, PDE6G, PDE6H, PDE7B, PDE8B, PDGFB, PDGFC, PDGFRA, PDHA1, PDHB, PDHX, PDLIM3, PDLIM5, PDP1, PDPK1, PDSS1, PDSS2, PDX1, PDXK, PDYN, PDZD7, PEAR1, PECAM1, PECR, PEMT, PENK, PEPD, PER1, PER2, PER3, PEX1, PEX10, PEX11B, PEX12, PEX13, PEX14, PEX16, PEX19, PEX2, PEX26, PEX3, PEX5, PEX6, PEX7, PFAS, PFKM, PFN1, PGAM1, PGAM2, PGAM5, PGBD1, PGC, PGD, PGK1, PGM1, PGR, PGRMC1, PHB, PHEX, PHF11, PHF2, PHF3, PHF6, PHF8, PHGDH, PHIP, PHKA1, PHKA2, PHKB, PHKG2, PHLDA2, PHLPP2, PHOX2A, PHOX2B, PHYH, PI3, PICALM, PICK1, PIEZO2, PIF1, PIGA, PIGL, PIGM, PIGN, PIGO, PIGR, PIGV, PIK3C2G, PIK3C3, PIK3CA, PIK3CB, PIK3CD, PIK3CG, PIK3R1, PIK3R2, PIK3R4, PIK3R5, PIKFYVE, PIN1, PINK1, PIP4K2A, PIP5K1B, PIP5K1C, PITPNM3, PITX1, PITX2, PITX3, PIWIL3, PKD1, PKD1L1, PKD2, PKHD1, PKLR, PKM, PKN3, PKP1, PKP2, PLA2G10, PLA2G2A, PLA2G2D, PLA2G4A, PLA2G4C, PLA2G5, PLA2G6, PLA2G7, PLAGL1, PLAT, PLAU, PLAUR, PLCB1, PLCB4, PLCD1, PLCE1, PLCG2, PLCZ1, PLD2, PLEC, PLEKHG4, PLEKHG5, PLEKHM1, PLG, PLIN1, PLIN4, PLN, PLOD1, PLOD2, PLOD3, PLP1, PLP2, PLTP, PLXND1, PMAIP1, PML, PMM2, PMP22, PMPCA, PMS1, PMS2, PMS2P3, PNKD, PNKP, PNMT, PNP, PNPLA1, PNPLA2, PNPLA3, PNPLA6, PNPO, POF1B, POGZ, POLB, POLD1, POLE2, POLG, POLG2, POLH, POLL, POLR1C, POLR1D, POLR2E, POLR2M, POLR3A, POLR3B, POLRMT, POMC, POMGNT1, POMP, POMT1, POMT2, PON1, PON2, PON3, POP1, POR, PORCN, POU1F1, POU3F4, POU4F3, POU5F1, POU5F1B, POU6F2, PPARA, PPARD, PPARG, PPARGC1A, PPARGC1B, PPAT, PPIA, PPIB, PPIG, PPM1D, PPOX, PPP1R1A, PPP1R3A, PPP1R3C, PPP2R1A, PPP2R1B, PPP2R2B, PPP2R2C, PPT1, PQBP1, PRB1, PRB3, PRB4, PRCD, PRCP, PRDM2, PRDM5, PRDM9, PREPL, PRF1, PRG4, PRICKLE1, PRICKLE2, PRKAA2, PRKACA, PRKAG2, PRKAG3, PRKAR1A, PRKCB, PRKCG, PRKCH, PRKCSH, PRKD3, PRKDC, PRKRA, PRL, PRLHR, PRLR, PRM1, PRM2, PRMT3, PRMT7, PRND, PRNP, PROC, PROCR, PRODH, PROK1, PROK2, PROKR1, PROKR2, PROM1, PROP1, PROS1, PROZ, PRPF3, PRPF31, PRPF6, PRPF8, PRPH, PRPH2, PRPS1, PRRC2A, PRRT2, PRRX1, PRSS1, PRSS12, PRSS3P2, PRSS56, PRSS8, PRX, PRY, PRY2, PSAP, PSAT1, PSCA, PSEN1, PSEN2, PSENEN, PSMA6, PSMB8, PSMB9, PSMC3IP, PSMD7, PSPH, PSPN, PSTPIP1, PTAFR, PTCD1, PTCH1, PTCH2, PTCHD1, PTCHD3, PTCSC3, PTEN, PTF1A, PTGDR, PTGDR2, PTGDS, PTGER2, PTGER4, PTGES2, PTGIR, PTGIS, PTGS1, PTGS2, PTH, PTH1R, PTHLH, PTK7, PTPN1, PTPN11, PTPN13, PTPN14, PTPN2, PTPN21, PTPN22, PTPN6, PTPRB, PTPRC, PTPRCAP, PTPRD, PTPRF, PTPRJ, PTPRK, PTPRO, PTPRQ, PTPRT, PTS, PUS1, PVR, PVT1, PWRN1, PXDN, PYCR1, PYGL, PYGM, PYY, PZP, QDPR, QKI, RAB11FIP5, RAB18, RAB23, RAB27A, RAB28, RAB2A, RAB39B, RAB3GAP1, RAB3GAP2, RAB40AL, RAB7A, RABGGTA, RABL6, RAC1, RAC2, RAD21, RAD21L1, RAD23B, RAD50, RAD51, RAD51C, RAD51D, RAD52, RAD54B, RAD54L, RAD9A, RAET1L, RAF1, RAG1, RAG2, RAI1, RALGAPA1, RALGDS, RANBP2, RANGRF, RAPSN, RARS2, RASA1, RASGRP2, RASSF1, RAX, RAX2, RB1, RB1CC1, RBBP8, RBFOX1, RBM10, RBM15, RBM20, RBM28, RBM8A, RBMXL2, RBMY1A1, RBP3, RBP4, RCAN1, RD3, RDH12, RDH5, RDX, RECQL4, REEP1, RELN, REN, REPS2, RET, RETN, REV3L, RFC2, RFT1, RFWD2, RFX2, RFX5, RFX6, RFX8, RFXANK, RFXAP, RGMA, RGR, RGS2, RGS6, RGS7, RGS9, RGS9BP, RHAG, RHBDF2, RHCE, RHD, RHO, RHOB, RHOG, RHPN2, RIMS1, RIMS3, RIN2, RIPK3, RIPK4, RLBP1, RMI1, RMRP, RNASE3, RNASEH2A, RNASEH2B, RNASEH2C, RNASEL, RNASET2, RNF113A, RNF114, RNF135, RNF139, RNF168, RNF170, RNF213, RNLS, RNU4ATAC, ROBO1, ROBO2, ROBO3, ROCK1, ROCK2, ROGDI, ROM1, ROPN1L, ROR2, RORA, ROS1, RP1, RP1L1, RP2, RP9, RPA1, RPA4, RPE65, RPGR, RPGRIP1, RPGRIP1L, RPH3AL, RPIA, RPL10, RPL11, RPL21, RPL35A, RPL36, RPL5, RPN2, RPS10, RPS15, RPS17, RPS19, RPS24, RPS26, RPS27A, RPS3, RPS4Y2, RPS6KA3, RPS6KB1, RPS6KL1, RPS7, RPTOR, RRH, RRM1, RRM2B, RRP1B, RS1, RSPH4A, RSPH9, RSPO1, RSPO4, RSRC1, RTN2, RTN4R, RUNX1, RUNX2, RUNX3, RUVBL1, RXFP2, RXRG, RYK, RYR1, RYR2, RYR3, S100A14, S100B, S1PR1, SAA1, SAA2, SACS, SAG, SAGE1, SALL1, SALL4, SAMD9, SAMHD1, SAR1B, SARDH, SARS2, SART1, SART3, SAT1, SATB2, SATL1, SBDS, SBF2, SBNO1, SCAP, SCARB1, SCARB2, SCARF2, SCG2, SCG3, SCGB1A1, SCGB1D2, SCGB3A2, SCN10A, SCN1A, SCN1B, SCN2A, SCN2B, SCN3A, SCN3B, SCN4A, SCN4B, SCN5A, SCN8A, SCN9A, SCNN1A, SCNN1B, SCNN1G, SCO1, SCO2, SCP2, SCRIB, SCT, SCUBE2, SDC3, SDCCAG8, SDHA, SDHAF1, SDHAF2, SDHB, SDHC, SDHD, SEC23A, SEC23B, SEC63, SECISBP2, SEL1L, SELE, SELL, SELP, SELPLG, SEMA3A, SEMA3E, SEMA4A, SEMA4C, SEMA4G, SEMA6D, SEMG1, SEPSECS, SEPT12, SEPT9, SERPINA1, SERPINA10, SERPINA3, SERPINA6, SERPINA7, SERPINB11, SERPINB3, SERPINB5, SERPINB6, SERPINC1, SERPIND1, SERPINE1, SERPINF1, SERPINF2, SERPING1, SERPINH1, SERPINI1, SERPINI2, SERTAD1, SESN2, SETBP1, SETD2, SETDB2, SETX, SEZ6, SEZ6L2, SF3B4, SFTPA1, SFTPA2, SFTPB, SFTPC, SFTPD, SGCA, SGCB, SGCD, SGCE, SGCG, SGK1, SGSH, SH2B1, SH2B3, SH2D1A, SH3BP2, SH3GL1, SH3PXD2B, SH3TC2, SHANK2, SHANK3, SHBG, SHH, SHMT1, SHOC2, SHOX, SHROOM3, SHROOM4, SI, SIAE, SIGLEC12, SIGLEC14, SIGLEC16, SIGMAR1, SIL1, SIM1, SIPA1, SIRT1, SIRT3, SIRT5, SIX1, SIX2, SIX3, SIX5, SIX6, SKI, SKIV2L, SLBP, SLC10A1, SLC10A2, SLC11A1, SLC11A2, SLC12A1, SLC12A3, SLC12A6, SLC13A2, SLC14A1, SLC14A2, SLC15A1, SLC16A1, SLC16A12, SLC16A2, SLC16A3, SLC17A1, SLC17A3, SLC17A5, SLC17A8, SLC18A1, SLC19A1, SLC19A2, SLC19A3, SLC1A1, SLC1A2, SLC1A3, SLC20A2, SLC22A1, SLC22A11, SLC22A12, SLC22A14, SLC22A2, SLC22A3, SLC22A4, SLC22A5, SLC22A6, SLC22A9, SLC23A1, SLC24A1, SLC24A2, SLC24A5, SLC25A12, SLC25A13, SLC25A15, SLC25A19, SLC25A20, SLC25A22, SLC25A3, SLC25A38, SLC25A39, SLC25A4, SLC26A1, SLC26A10, SLC26A2, SLC26A3, SLC26A4, SLC26A5, SLC26A6, SLC26A9, SLC27A4, SLC27A5, SLC28A1, SLC28A2, SLC28A3, SLC29A1, SLC29A2, SLC29A3, SLC29A4, SLC2A1, SLC2A10, SLC2A2, SLC2A4, SLC2A9, SLC30A10, SLC30A2, SLC30A5, SLC30A8, SLC31A1, SLC33A1, SLC34A1, SLC34A2, SLC34A3, SLC35A1, SLC35C1, SLC35D1, SLC35G2, SLC36A2, SLC37A4, SLC39A13, SLC39A4, SLC3A1, SLC40A1, SLC41A1, SLC44A2, SLC45A2, SLC46A1, SLC47A1, SLC47A2, SLC4A1, SLC4A10, SLC4A11, SLC4A3, SLC4A4, SLC52A1, SLC52A3, SLC5A1, SLC5A11, SLC5A2, SLC5A5, SLC5A7, SLC6A1, SLC6A11, SLC6A12, SLC6A13, SLC6A14, SLC6A18, SLC6A19, SLC6A2, SLC6A20, SLC6A3, SLC6A4, SLC6A5, SLC6A8, SLC7A1, SLC7A10, SLC7A2, SLC7A5, SLC7A7, SLC7A9, SLC8A1, SLC9A3R1, SLC9A6, SLC9A9, SLCO1A2, SLCO1B1, SLCO1B3, SLCO1C1, SLCO2A1, SLCO2B1, SLFN5, SLIT3, SLITRK1, SLURP1, SLX4, SMAD1, SMAD2, SMAD3, SMAD4, SMAD6, SMAD7, SMAD9, SMARCA2, SMARCA4, SMARCAD1, SMARCAL1, SMARCB1, SMARCE1, SMC1A, SMC1B, SMC3, SMG1, SMG6, SMN1, SMN2, SMNDC1, SMOC1, SMOC2, SMPD1, SMPX, SMS, SMUG1, SMYD3, SNAI2, SNAP29, SNAPC4, SNAPC5, SNCA, SNCAIP, SNCB, SNIP1, SNORD115-1, SNORD116-1, SNORD116-10, SNORD50A, SNRK, SNRNP200, SNRPN, SNTA1, SNTG2, SNURF, SNX10, SNX19, SNX3, SOBP, SOCS1, SOCS3, SOD1, SOD2, SOD3, SOGA3, SOHLH1, SORBS1, SORCS1, SORL1, SORT1, SOS1, SOST, SOX10, SOX17, SOX18, SOX2, SOX3, SOX5, SOX6, SOX8, SOX9, SP100, SP110, SP7, SP8, SPAG16, SPAG17, SPANXN5, SPAST, SPATA13, SPATA16, SPATA21, SPATA31C1, SPATA7, SPECC1L, SPG11, SPG21, SPG7, SPI1, SPINK1, SPINK5, SPINT2, SPP1, SPR, SPRED1, SPRN, SPRR3, SPRY2, SPRY3, SPTA1, SPTAN1, SPTB, SPTBN2, SPTBN5, SPTLC1, SPTLC2, SQSTM1, SRC, SRCAP, SRD5A2, SRD5A3, SREBF1, SREBF2, SREK1, SRGAP2, SRGAP3, SRI, SRP72, SRPX, SRPX2, SRR, SRY, SSH1, SST, SSTR2, SSTR5, SSX7, ST14, ST3GAL3, ST3GAL5, ST5, ST7, ST8SIA2, STAR, STAT1, STAT3, STAT5B, STAT6, STEAP3, STEAP4, STH, STIL, STIM1, STK10, STK11, STK19, STK3, STK32A, STK33, STK39, STK4, STMN1, STOX1, STRA6, STRADA, STRC, STS, STX11, STX16, STX1A, STXBP1, STXBP2, STXBP5, SUCLA2, SUCLG1, SUFU, SULF1, SULT1A1, SULT1A3, SULT1C2, SULT1E1, SULT2A1, SULT2B1, SULT4A1, SUMF1, SUMO1, SUMO4, SUN2, SUOX, SUPT16H, SURF1, SV2B, SYCP3, SYN1, SYN3, SYNE1, SYNE2, SYNGAP1, SYNGR1, SYNM, SYNPO, SYP, SYT11, SYT14, SYT2, SYTL3, SYTL5, T, TAAR6, TAAR9, TAB2, TAC3, TACO1, TACR3, TACSTD2, TAF1, TAF15, TAF1C, TAF1L, TAF2, TAF7L, TAL1, TALDO1, TAP1, TAP2, TAPBP, TARDBP, TAS1R1, TAS1R2, TAS1R3, TAS2R16, TAS2R19, TAS2R3, TAS2R38, TAS2R43, TAS2R46, TAS2R50, TAS2R9, TAT, TAZ, TBC1D1, TBC1D23, TBC1D24, TBC1D4, TBCE, TBK1, TBL1X, TBL1XR1, TBL1Y, TBP, TBX1, TBX10, TBX15, TBX19, TBX2, TBX20, TBX21, TBX22, TBX3, TBX4, TBX5, TBX6, TBXA2R, TBXAS1, TCAP, TCF21, TCF4, TCF7, TCF7L1, TCF7L2, TCIRG1, TCN1, TCN2, TCOF1, TCP1, TCTE1, TCTE3, TCTN1, TCTN2, TDGF1, TDO2, TDP1, TDRD7, TEAD1, TECR, TECTA, TEK, TEKT2, TENM4, TEP1, TERC, TERT, TET1, TEX14, TF, TFAM, TFAP2A, TFAP2B, TFB1M, TFCP2, TFF1, TFPI, TFR2, TFRC, TG, TGFB1, TGFB2, TGFB3, TGFBI, TGFBR1, TGFBR2, TGFBR3, TGFBRAP1, TGIF1, TGM1, TGM2, TGM5, TGM6, TH, THADA, THAP1, THBD, THBS1, THBS2, THBS4, THPO, THRA, THRB, THSD7A, TICAM1, TIMM44, TIMM8A, TIMP1, TIMP2, TIMP3, TINAG, TINF2, TIRAP, TJP2, TK2, TLK1, TLL1, TLR1, TLR10, TLR2, TLR3, TLR4, TLR5, TLR6, TLR7, TLR8, TLR9, TM4SF19, TMC1, TMC6, TMC8, TMCO1, TMEM114, TMEM126A, TMEM127, TMEM135, TMEM138, TMEM165, TMEM173, TMEM185A, TMEM187, TMEM2, TMEM216, TMEM237, TMEM39A, TMEM43, TMEM67, TMEM70, TMEM8A, TMEM9, TMIE, TMLHE, TMPO, TMPRSS11A, TMPRSS15, TMPRSS3, TMPRSS4, TMPRSS5, TMPRSS6, TNC, TNF, TNFAIP2, TNFAIP3, TNFRSF10A, TNFRSF10B, TNFRSF11A, TNFRSF11B, TNFRSF13B, TNFRSF13C, TNFRSF1A, TNFRSF1B, TNFRSF25, TNFRSF4, TNFSF10, TNFSF11, TNFSF13B, TNFSF14, TNFSF15, TNFSF4, TNFSF8, TNKS, TNNC1, TNNI2, TNNI3, TNNI3K, TNNT1, TNNT2, TNNT3, TNP1, TNR, TNS3, TNXB, TOMM40, TOP1MT, TOPBP1, TOPORS, TOR1A, TOX3, TP53, TP53AIP1, TP53BP1, TP53I3, TP53RK, TP63, TP73, TPCN2, TPH1, TPH2, TPI1, TPK1, TPM1, TPM2, TPM3, TPMT, TPO, TPP1, TPP2, TPRN, TPSB2, TPTE, TRAC, TRADD, TRAF3, TRAF3IP1, TRAF3IP2, TRAF6, TRAK2, TRAPPC10, TRAPPC2, TRAPPC9, TRB, TRBV9, TRD, TRDN, TREM2, TRERF1, TREX1, TRHR, TRIB1, TRIB3, TRIL, TRIM17, TRIM21, TRIM22, TRIM32, TRIM37, TRIM5, TRIO, TRIOBP, TRIP11, TRIP12, TRMT1, TRMU, TROAP, TROVE2, TRPA1, TRPC3, TRPC4, TRPC5, TRPC6, TRPM1, TRPM2, TRPM3, TRPM4, TRPM6, TRPM7, TRPS1, TRPV1, TRPV3, TRPV4, TRPV5, TRRAP, TSC1, TSC2, TSEN2, TSEN34, TSEN54, TSFM, TSHB, TSHR, TSHZ1, TSLP, TSPAN12, TSPAN17, TSPAN7, TSPO, TSPYL1, TSSC4, TSSK2, TSSK3, TSSK4, TST, TTBK2, TTC19, TTC21B, TTC37, TTC8, TTI2, TTLL11, TTN, TTPA, TTR, TTTY13, TUBA1A, TUBA8, TUBB1, TUBB2B, TUBB3, TUBGCP4, TUBGCP5, TUBGCP6, TUFM, TULP1, TULP3, TUSC3, TWIST1, TWIST2, TXN2, TXNRD2, TYK2, TYMP, TYMS, TYR, TYROBP, TYRP1, UBA1, UBA3, UBAC2, UBD, UBE2A, UBE2B, UBE2I, UBE2NL, UBE3A, UBE3C, UBIAD1, UBN2, UBQLN2, UBR1, UBR3, UBR7, UCHL1, UCP1, UCP2, UCP3, UGT1A1, UGT1A10, UGT1A3, UGT1A4, UGT1A5, UGT1A6, UGT1A7, UGT1A8, UGT1A9, UGT2A1, UGT2A3, UGT2B10, UGT2B15, UGT2B17, UGT2B28, UGT2B7, UHRF1BP1, UIMC1, UMOD, UMPS, UNC119, UNC13D, UNC5C, UNC5CL, UNC80, UNC93A, UNC93B1, UNG, UNKL, UPB1, UPF3B, UPK3A, UQCRB, UQCRQ, UROC1, UROD, UROS, USB1, USF1, USH1C, USH1G, USH2A, USP15, USP24, USP26, USP46, USP7, USP9X, USP9Y, UTF1, UTRN, UTS2, UVSSA, VAMP7, VANGL1, VANGL2, VAPB, VAX1, VCAM1, VCAN, VCL, VCP, VCX3A, VCY, VDR, VEGFA, VHL, VIM, VIP, VIPAS39, VIPR2, VKORC1, VLDLR, VMA21, VNN1, VPREB1, VPS13A, VPS13B, VPS33B, VPS35, VRK1, VSIG4, VSX1, VSX2, VTN, VWF, WAS, WASF3, WDFY3, WDFY4, WDPCP, WDR11, WDR13, WDR19, WDR35, WDR36, WDR4, WDR45, WDR45B, WDR62, WDR72, WDR81, WFS1, WIPF1, WISP3, WNK1, WNK4, WNT10A, WNT10B, WNT3, WNT4, WNT5A, WNT5B, WNT7A, WRAP53, WRN, WT1, WT1-AS, WWC1, WWOX, XBP1, XDH, XIAP, XIST, XK, XKR4, XKRY, XPA, XPC, XPNPEP2, XPNPEP3, XRCC1, XRCC2, XRCC3, XRCC4, XRCC5, XRCC6, XYLT1, XYLT2, YAP1, YARS, YARS2, YBX2, YTHDF2, YWHAE, YY1, ZAN, ZAP70, ZBTB16, ZBTB18, ZBTB24, ZBTB40, ZBTB41, ZC3H14, ZC3H3, ZC3HAV1, ZCCHC12, ZCCHC13, ZCCHC8, ZDHHC15, ZDHHC8, ZDHHC9, ZEB1, ZEB2, ZFAT, ZFHX3, ZFHX4, ZFP36, ZFP36L1, ZFP57, ZFP90, ZFPM2, ZFY, ZFYVE26, ZFYVE27, ZHX3, ZIC1, ZIC2, ZIC3, ZMPSTE24, ZMYND11, ZNF202, ZNF213, ZNF224, ZNF24, ZNF335, ZNF350, ZNF365, ZNF385B, ZNF41, ZNF469, ZNF480, ZNF507, ZNF513, ZNF526, ZNF592, ZNF627, ZNF644, ZNF674, ZNF711, ZNF750, ZNF75D, ZNF80, ZNF804A, ZNF81, ZNHIT6, ZNRF1, ZPBP, ZPBP2.*

# Supplementary Table S2. Primers used for site-directed mutagenesis to generate the *MUC13* missense variant (p.Arg503Ser)

| Forward primer | 5'- CAGATGCAAAATCCCTATTCAAGCCACAGCAGCATGCC -3' |
| --- | --- |
| Reverse primer | 5’- GGCATGCTGCTGTGgCTTGAATAGGGATTTTGCATCTG -3’ |

*RefSeq NM_033049.4:c.1509A>C; NP_149038.3:p.Arg503Ser; Ensembl ENST00000311075.3:c.1506A>C, p.Arg502Ser

# Supplementary Table S3. Descriptive statistics of biochemical parameters in the study cohort

|  | Ferritin  (microg/L) | Iron (mg/L) | Transferrin  (g/L) | Transferrin  saturation (%) | Alkaline phosphate (U/L) | ALAT (U/L) | ASAT (U/L) | GGT (U/L) | C-reactive protein  (mg/L) |
| --- | --- | --- | --- | --- | --- | --- | --- | --- | --- |
| Median | 120 | 0.91 | 2.56 | 26 | 74 | 33 | 29 | 44 | 2.3 |
| 25th percentile | 43 | 0.70 | 2.28 | 19 | 58 | 21 | 20 | 23 | 0.9 |
| 75th percentile | 270 | 1.22 | 2.89 | 34 | 99 | 54 | 38 | 81 | 4.0 |
| Minimum | 2 | 0.16 | 1.24 | 4 | 22 | 5 | 9 | 5 | 0.0 |
| Maximum | 4270 | 5.00 | 4.15 | 94 | 868 | 184 | 189 | 1239 | 9.9 |
| Missing | 0 | 46 | 94 | 95 | 128 | 95 | 95 | 149 | 202 |

Note. Ferritin (µg/L), iron (mg/L), iron-binding capacity saturation (%), transferrin (g/L), aspartate aminotransferases (ASAT) (U/L), alanine aminotransferases (ALAT) (U/L), γ-glutamyltransferase (U/L), alkaline phosphatase (U/L);

# Supplementary Table S4. Results of the clinical exome-based association study reporting the top loci associated with plasma ferritin levels in combined multivariate and collapsing regression analysis

| Gene | Chr | Start* | Stop* | Strand | *P*-Value | Bonferroni  *P*-value | # Markers  Total | # in  Bin=0 | # in  Bin=1 | Bin 0  Beta | Bin 0  Beta SE | Bin 1  Beta | Bin 1  Beta SE |
| --- | --- | --- | --- | --- | --- | --- | --- | --- | --- | --- | --- | --- | --- |
| *SOGA3* | 6 | 127759551 | 127840169 | - | 1.82E-23 | 7.39E-20 | 5 | 5 | 0 | 460.3 | 41.6 |  |  |
| *PRKAG3* | 2 | 219687104 | 219696526 | - | 3.71E-15 | 1.50E-11 | 14 | 11 | 3 | 454.8 | 52.1 | -13.7 | 20.8 |
| *STAT5B* | 17 | 40351195 | 40428409 | - | 5.21E-13 | 2.11E-09 | 4 | 4 | 0 | 531.5 | 69.8 |  |  |
| *POU4F3* | 5 | 145718402 | 145721291 | + | 1.49E-12 | 6.03E-09 | 3 | 3 | 0 | 713.5 | 95.7 |  |  |
| *GBA* | 1 | 155204243 | 155214418 | - | 2.55E-12 | 1.03E-08 | 11 | 10 | 1 | -74.1 | 39.9 | 711.4 | 95.3 |
| *PICALM* | 11 | 85668218 | 85780902 | - | 2.91E-12 | 1.18E-08 | 8 | 8 | 0 | 433.0 | 59.0 |  |  |
| *SULT1E1* | 4 | 70706930 | 70725863 | - | 8.69E-12 | 3.53E-08 | 2 | 1 | 1 | 418.1 | 56.5 | -49.2 | 26.7 |
| *NCR3* | 6 | 31556672 | 31560783 | - | 1.41E-11 | 5.73E-08 | 7 | 4 | 3 | 714.3 | 97.5 | -1.5 | 30.2 |
| *KNG1* | 3 | 186435148 | 186462199 | + | 4.27E-11 | 1.73E-07 | 12 | 6 | 6 | 478.3 | 66.6 | -153.9 | 161.1 |
| *MSTN* | 2 | 190920426 | 190927455 | - | 5.91E-11 | 2.40E-07 | 6 | 5 | 1 | 18.1 | 60.7 | 313.4 | 43.5 |
| *SLC46A1* | 17 | 26721661 | 26733230 | - | 1.01E-10 | 4.08E-07 | 8 | 7 | 1 | 435.1 | 61.2 | 46.0 | 93.3 |
| *AGK* | 7 | 141251210 | 141355044 | + | 1.13E-10 | 4.59E-07 | 5 | 5 | 0 | 556.5 | 82.7 |  |  |
| *SAT1* | X | 23801275 | 23804340 | + | 1.16E-10 | 4.72E-07 | 2 | 2 | 0 | 588.9 | 87.5 |  |  |
| *SPINT2* | 19 | 38755213 | 38783255 | + | 1.21E-10 | 4.90E-07 | 8 | 7 | 1 | 329.2 | 47.8 | -61.7 | 33.5 |
| *ZBTB41* | 1 | 197122812 | 197170423 | - | 1.25E-10 | 5.06E-07 | 4 | 1 | 3 | 44.1 | 92.8 | 246.0 | 34.8 |
| *FGFBP1* | 4 | 15937200 | 15940363 | - | 1.32E-10 | 5.37E-07 | 3 | 3 | 0 | 417.1 | 62.2 |  |  |
| *ZNF674* | X | 46357160 | 46404856 | - | 1.76E-10 | 7.15E-07 | 3 | 3 | 0 | 387.6 | 58.2 |  |  |
| *ZNF224* | 19 | 44598514 | 44613979 | + | 2.06E-10 | 8.37E-07 | 6 | 5 | 1 | 233.5 | 96.1 | 262.6 | 40.5 |
| *PQBP1* | X | 48755213 | 48760420 | + | 2.24E-10 | 9.09E-07 | 3 | 2 | 1 | 611.1 | 88.1 | 37.6 | 21.7 |
| *GRIK2* | 6 | 101841584 | 102517958 | + | 2.63E-10 | 1.06E-06 | 9 | 4 | 5 | 356.2 | 78.9 | -134.3 | 34.0 |
| *POLR1D* | 13 | 28194880 | 28241548 | + | 2.68E-10 | 1.09E-06 | 1 | 0 | 1 |  |  | 513.2 | 78.0 |
| *NDUFS2* | 1 | 161167207 | 161184185 | + | 1.36E-09 | 5.50E-06 | 5 | 3 | 2 | 492.9 | 77.7 | -25.3 | 21.9 |
| *LPA* | 6 | 160952514 | 161085307 | - | 1.52E-09 | 6.15E-06 | 22 | 15 | 7 | 182.8 | 28.3 | -63.9 | 24.5 |
| *AIP* | 11 | 67250497 | 67258574 | + | 1.99E-09 | 8.08E-06 | 4 | 2 | 2 | 524.6 | 82.5 | -93.8 | 51.8 |
| *GSTM4* | 1 | 110198721 | 110208119 | + | 2.41E-09 | 9.79E-06 | 4 | 4 | 0 | 383.1 | 61.9 |  |  |
| *ABHD5* | 3 | 43732362 | 43775863 | + | 2.55E-09 | 1.03E-05 | 3 | 3 | 0 | 243.8 | 39.4 |  |  |
| *DNAJC9-AS1* | 10 | 75007125 | 75036742 | + | 1.10E-08 | 4.46E-05 | 2 | 2 | 0 | 501.6 | 84.8 |  |  |
| *MRPS16* | 10 | 75008607 | 75012402 | - | 1.10E-08 | 4.46E-05 | 2 | 2 | 0 | 501.6 | 84.8 |  |  |
| *TNFRSF1A* | 12 | 6437937 | 6451242 | - | 1.52E-08 | 6.18E-05 | 9 | 6 | 3 | 359.3 | 62.0 | -93.4 | 31.2 |
| *SARS2* | 19 | 39405906 | 39421403 | - | 2.35E-08 | 9.52E-05 | 13 | 11 | 2 | 262.6 | 44.9 | 21.1 | 20.9 |
| *ZNF644* | 1 | 91380861 | 91487592 | - | 2.82E-08 | 1.14E-04 | 11 | 10 | 1 | 5.8 | 49.8 | 188.2 | 30.8 |
| *CASP8* | 2 | 202098166 | 202152434 | + | 2.92E-08 | 1.18E-04 | 5 | 2 | 3 | 415.4 | 118.8 | -128.8 | 24.8 |
| *PON1* | 7 | 94926988 | 94953844 | - | 3.31E-08 | 1.34E-04 | 8 | 6 | 2 | -143.1 | 85.1 | -199.3 | 34.9 |
| *PROCR* | 20 | 33759875 | 33765165 | + | 4.01E-08 | 1.63E-04 | 5 | 4 | 1 | 474.5 | 78.6 | 14.1 | 32.4 |
| *PDCD1* | 2 | 242792036 | 242801046 | - | 4.34E-08 | 1.76E-04 | 5 | 4 | 1 | -282.9 | 65.6 | 758.2 | 183.1 |
| *RANBP2* | 2 | 109335938 | 109402265 | + | 4.71E-08 | 1.91E-04 | 26 | 23 | 3 | -98.6 | 28.5 | -107.4 | 22.0 |
| *IGHMBP2* | 11 | 68671359 | 68708069 | + | 6.82E-08 | 2.76E-04 | 15 | 8 | 7 | 362.4 | 65.1 |  |  |
| *IGF1R* | 15 | 99191768 | 99507759 | + | 7.31E-08 | 2.97E-04 | 9 | 9 | 0 | 425.7 | 76.7 |  |  |
| *EFTUD2* | 17 | 42927316 | 42976813 | - | 7.66E-08 | 3.11E-04 | 5 | 3 | 2 | 74.2 | 68.6 | -184.0 | 31.2 |
| *PPT1* | 1 | 40537133 | 40562924 | - | 1.08E-07 | 4.40E-04 | 6 | 5 | 1 | 355.1 | 61.4 | 44.1 | 38.5 |
| *ITK* | 5 | 156607851 | 156682195 | + | 1.31E-07 | 5.32E-04 | 4 | 3 | 1 | 384.9 | 66.5 | 47.2 | 82.2 |
| *KIAA0100* | 17 | 26941458 | 26972177 | - | 1.42E-07 | 5.74E-04 | 19 | 17 | 2 | 194.5 | 34.0 | 6.5 | 21.9 |
| *MMP20* | 11 | 102447563 | 102496063 | - | 1.95E-07 | 7.92E-04 | 8 | 5 | 3 | 324.6 | 63.6 | 119.9 | 50.6 |
| *NT5DC1* | 6 | 116422016 | 116570660 | + | 2.01E-07 | 8.13E-04 | 6 | 4 | 2 | 10.9 | 116.5 | -124.8 | 21.8 |
| *STEAP3* | 2 | 119981410 | 120023228 | + | 2.09E-07 | 8.48E-04 | 5 | 4 | 1 | -199.0 | 61.8 | -261.4 | 50.9 |
| *PIP5K1C* | 19 | 3630181 | 3700466 | - | 2.31E-07 | 9.38E-04 | 8 | 8 | 0 | 290.7 | 54.6 |  |  |
| *ACP5* | 19 | 11685475 | 11689790 | - | 2.34E-07 | 9.48E-04 | 7 | 5 | 2 | 422.5 | 82.4 | 59.6 | 24.3 |
| *TRPM4* | 19 | 49661049 | 49715093 | + | 2.49E-07 | 1.01E-03 | 23 | 22 | 1 | -41.1 | 32.3 | 294.7 | 54.4 |
| *CCDC170* | 6 | 151815152 | 151942328 | + | 2.62E-07 | 1.06E-03 | 13 | 7 | 6 | 22.0 | 75.1 | -488.4 | 86.1 |
| *ANGPT2* | 8 | 6357153 | 6420766 | - | 2.92E-07 | 1.18E-03 | 3 | 2 | 1 | 498.1 | 118.8 | 211.9 | 52.5 |
| *PPARGC1B* | 5 | 149109873 | 149234585 | + | 3.53E-07 | 1.43E-03 | 19 | 13 | 6 | 295.1 | 58.8 | 36.8 | 22.0 |
| *DOC2A* | 16 | 30016835 | 30024917 | - | 3.70E-07 | 1.50E-03 | 8 | 7 | 1 | 267.1 | 51.3 | 20.8 | 22.1 |
| *SERPINF1* | 17 | 1665354 | 1680859 | + | 3.73E-07 | 1.51E-03 | 12 | 9 | 3 | 267.9 | 57.0 | -64.8 | 23.3 |
| *KRAS* | 12 | 25358180 | 25403863 | - | 4.36E-07 | 1.77E-03 | 5 | 4 | 1 | 391.7 | 71.0 | -43.6 | 55.5 |
| *WDPCP* | 2 | 63346694 | 63815611 | - | 4.38E-07 | 1.78E-03 | 15 | 13 | 2 | -140.9 | 31.0 | -84.1 | 22.2 |
| *JPH2* | 20 | 42735230 | 42815828 | - | 5.04E-07 | 2.04E-03 | 6 | 5 | 1 | 313.1 | 56.5 | -10.8 | 23.7 |
| *SLC14A1* | 18 | 43304146 | 43332485 | + | 5.09E-07 | 2.06E-03 | 8 | 4 | 4 | -23.6 | 105.9 | -468.4 | 84.6 |
| *KCNJ10* | 1 | 160007257 | 160039950 | - | 5.26E-07 | 2.13E-03 | 5 | 3 | 2 | 437.8 | 79.2 | 0.5 | 37.6 |
| *LIPE* | 19 | 42905666 | 42931540 | - | 7.66E-07 | 3.11E-03 | 12 | 11 | 1 | 258.3 | 52.7 | -235.8 | 100.2 |
| *SLC5A5* | 19 | 17982754 | 18005983 | + | 8.09E-07 | 3.28E-03 | 8 | 7 | 1 | 218.0 | 42.6 | 50.0 | 22.2 |
| *CASK* | X | 41374187 | 41782807 | - | 1.05E-06 | 4.27E-03 | 5 | 1 | 4 | 130.0 | 31.4 | 6.6 | 28.0 |
| *MEFV* | 16 | 3292027 | 3306633 | - | 1.14E-06 | 4.64E-03 | 26 | 19 | 7 | -134.9 | 40.7 | -136.9 | 30.5 |
| *SLC45A2* | 5 | 33944728 | 33984798 | - | 1.16E-06 | 4.70E-03 | 9 | 6 | 3 | -331.7 | 61.7 | 295.8 | 62.2 |
| *GLCCI1* | 7 | 8008427 | 8128710 | + | 1.25E-06 | 5.06E-03 | 2 | 2 | 0 | 366.6 | 73.8 |  |  |
| *CCRL2* | 3 | 46448750 | 46451014 | + | 1.42E-06 | 5.75E-03 | 11 | 4 | 7 | -191.6 | 91.1 | 142.6 | 27.9 |
| *LOXL2* | 8 | 23154410 | 23261633 | - | 1.52E-06 | 6.18E-03 | 12 | 10 | 2 | 227.5 | 42.8 | 14.7 | 23.0 |
| *CBS* | 21 | 44473301 | 44496983 | - | 1.59E-06 | 6.43E-03 | 9 | 8 | 1 | 366.7 | 70.1 | 5.8 | 33.0 |
| *TBX6* | 16 | 30097114 | 30103245 | - | 1.63E-06 | 6.60E-03 | 5 | 4 | 1 | 306.9 | 57.9 | 19.7 | 44.9 |
| *ATP8A2* | 13 | 25946112 | 26599989 | + | 1.64E-06 | 6.67E-03 | 10 | 8 | 2 | 243.3 | 55.0 | -65.5 | 23.1 |
| *EIF2B3* | 1 | 45316194 | 45452267 | - | 1.66E-06 | 6.73E-03 | 6 | 4 | 2 | 131.7 | 110.7 | 312.7 | 59.1 |
| *TMPRSS4* | 11 | 117947793 | 117992605 | + | 1.75E-06 | 7.10E-03 | 7 | 3 | 4 | 303.9 | 64.5 | 69.2 | 24.1 |
| *IL10RA* | 11 | 117857109 | 117872198 | + | 1.76E-06 | 7.12E-03 | 30 | 25 | 5 | -114.8 | 29.5 | -80.3 | 24.4 |
| *TGFB1* | 19 | 41836228 | 41859827 | - | 1.97E-06 | 8.01E-03 | 9 | 5 | 4 | 232.8 | 48.3 | 66.7 | 24.7 |
| *MIP* | 12 | 56843286 | 56848441 | - | 2.09E-06 | 8.50E-03 | 4 | 2 | 2 |  |  | 295.2 | 60.8 |
| *TBL1XR1* | 3 | 176737132 | 176915270 | - | 2.59E-06 | 1.05E-02 | 4 | 2 | 2 | -142.9 | 166.8 | 122.6 | 23.6 |
| *CBR3-AS1* | 21 | 37504065 | 37528606 | - | 2.62E-06 | 1.06E-02 | 8 | 4 | 4 | 156.2 | 169.9 | -368.9 | 72.2 |
| *CBR3* | 21 | 37507377 | 37518860 | + | 2.62E-06 | 1.06E-02 | 8 | 4 | 4 | 156.2 | 169.9 | -368.9 | 72.2 |
| *IDH3B* | 20 | 2639041 | 2644862 | - | 2.63E-06 | 1.07E-02 | 8 | 6 | 2 | -259.0 | 53.9 | 28.3 | 22.6 |
| *NFKBIA* | 14 | 35870719 | 35873955 | - | 2.77E-06 | 1.12E-02 | 4 | 3 | 1 | -32.1 | 83.5 | -110.6 | 21.5 |
| *NEXN* | 1 | 78354309 | 78409580 | + | 2.91E-06 | 1.18E-02 | 6 | 5 | 1 | -111.0 | 119.8 | 116.6 | 23.4 |
| *CCR2* | 3 | 46395602 | 46402431 | + | 3.13E-06 | 1.27E-02 | 3 | 2 | 1 | 276.3 | 91.9 | -146.6 | 30.0 |
| *CD2AP* | 6 | 47445525 | 47594999 | + | 3.78E-06 | 1.53E-02 | 8 | 8 | 0 | 234.0 | 49.5 |  |  |
| *TBCD* | 17 | 80709941 | 80903790 | + | 4.13E-06 | 1.68E-02 | 8 | 7 | 1 | -218.1 | 70.3 | -133.1 | 31.5 |
| *ZNF750* | 17 | 80787310 | 80797898 | - | 4.13E-06 | 1.68E-02 | 8 | 7 | 1 | -218.1 | 70.3 | -133.1 | 31.5 |
| *RPS6KA3* | X | 20168029 | 20285027 | - | 5.15E-06 | 2.09E-02 | 1 | 1 | 0 | -286.3 | 61.4 |  |  |
| *SULT1A1* | 16 | 28616908 | 28634696 | - | 5.16E-06 | 2.09E-02 | 10 | 9 | 1 | -243.2 | 63.1 | -63.3 | 61.1 |
| *OAZ1* | 19 | 2269485 | 2273487 | + | 5.47E-06 | 2.22E-02 | 7 | 7 | 0 | 307.1 | 66.1 |  |  |
| *PCK1* | 20 | 56136166 | 56143177 | + | 5.56E-06 | 2.26E-02 | 13 | 9 | 4 | -191.9 | 47.8 | -90.1 | 28.0 |
| *NSDHL* | X | 151999607 | 152038273 | + | 5.89E-06 | 2.39E-02 | 1 | 1 | 0 | 336.3 | 72.7 |  |  |
| *GLA* | X | 100652791 | 100662913 | - | 6.91E-06 | 2.80E-02 | 2 | 1 | 1 |  |  | 211.4 | 46.0 |
| *TBX3* | 12 | 115108060 | 115121980 | - | 7.06E-06 | 2.86E-02 | 2 | 2 | 0 | 382.0 | 83.2 |  |  |
| *PREPL* | 2 | 44544746 | 44589001 | - | 7.11E-06 | 2.88E-02 | 14 | 11 | 3 | 186.9 | 43.3 | 42.7 | 22.0 |
| *GJB3* | 1 | 35246815 | 35251965 | + | 7.32E-06 | 2.97E-02 | 4 | 3 | 1 | -2.6 | 93.9 | 270.7 | 54.4 |
| *ZNF202* | 11 | 123594622 | 123612368 | - | 7.50E-06 | 3.04E-02 | 8 | 6 | 2 | -35.4 | 56.9 | 203.0 | 40.8 |
| *LCT* | 2 | 136545420 | 136594754 | - | 7.73E-06 | 3.13E-02 | 11 | 8 | 3 | 230.2 | 50.7 | 53.5 | 23.3 |
| *SCN8A* | 12 | 51985017 | 52206648 | + | 8.61E-06 | 3.49E-02 | 7 | 6 | 1 | -180.3 | 62.4 | -276.3 | 67.3 |
| *EPHA3* | 3 | 89156771 | 89531284 | + | 8.97E-06 | 3.64E-02 | 6 | 4 | 2 | 380.9 | 77.5 | -11.1 | 22.0 |
| *GNAT2* | 1 | 110145713 | 110162238 | - | 9.69E-06 | 3.93E-02 | 4 | 3 | 1 | 386.4 | 79.7 | -57.1 | 64.6 |
| *TRIM37* | 17 | 57060010 | 57184241 | - | 9.77E-06 | 3.96E-02 | 14 | 11 | 3 | 59.7 | 66.1 | 110.5 | 22.5 |
| *MUC13* | 3 | 124624289 | 124653595 | - | 9.83E-06 | 3.99E-02 | 7 | 4 | 3 | 286.7 | 58.5 | 11.1 | 32.4 |
| *DSPP* | 4 | 88529681 | 88538025 | + | 1.04E-05 | 4.21E-02 | 9 | 7 | 2 | 246.6 | 55.0 | 66.9 | 26.4 |
| *MPP4* | 2 | 202509597 | 202563367 | - | 1.06E-05 | 4.29E-02 | 12 | 8 | 4 | 215.8 | 56.6 | 74.7 | 31.2 |
| *TK2* | 16 | 66541906 | 66584194 | - | 1.07E-05 | 4.36E-02 | 4 | 4 | 0 | 190.1 | 42.3 |  |  |
| *CHRNA7* | 15 | 32322718 | 32465219 | + | 1.17E-05 | 4.73E-02 | 4 | 4 | 0 | 375.1 | 83.9 |  |  |
| *ATP2A1* | 16 | 28889809 | 28915787 | + | 1.20E-05 | 4.87E-02 | 10 | 7 | 3 | 330.8 | 68.3 | 10.1 | 22.3 |
| *SLC6A4* | 17 | 28521337 | 28562715 | - | 1.21E-05 | 4.89E-02 | 3 | 2 | 1 | 22.1 | 167.2 | 251.8 | 51.7 |

Abbreviations: Chr, chromosome; AAF, alternative allele frequency; Bonf-*P*, Bonferroni-corrected *P* value; SE, standard error.

* Genomic positions are reported according to GRCh37.

# Supplementary Table S5. Results of the Bayesian analysis of variance for the association between the 11 cross-validated genetic variants and plasma iron concentration

| Variant | Chr | Position | Ref/Alt | dbSNP  ID | Gene | AAF | ACMG | BF(m) | Ref-Ref  (AA) | | Ref-Alt  (AB) | | Alt-Alt  (BB) | |
| --- | --- | --- | --- | --- | --- | --- | --- | --- | --- | --- | --- | --- | --- | --- |
|  |  |  |  |  |  |  |  |  | **N** | **Mean,**  **Iron (mg/L)**  **(95% Cred. I)** | **N** | **Mean,**  **Iron (mg/L)**  **(95% Cred. I)** | **N** | **Mean,**  **Iron (mg/L)**  **(95% Cred. I)** |
| 3:124627024-SNV | 3 | 124627024 | T/G | rs1127233 | *MUC13* | 0.239 | B | 4000 | 249 | 0.98  (0.92–1.03) | 151 | 0.91  (0.84–0.99) | 37 | 1.36  (1.18–1.55) |
| 1:91404256-SNV | 1 | 91404256 | C/A | rs41286761 | *ZNF644* | 0.022 | B | 0.54 | 367 | 0.97  (0.92–1.02) | 70 | 1.08  (0.97–1.18) | — | — |
| 3:176765269-SNV | 3 | 176765269 | T/C | rs3749234 | *TBL1XR1* | 0.211 | B | 0.05 | 280 | 0.98  (0.92–1.04) | 137 | 1.00  (0.94–1.07) | 20 | 1.00  (0.77–1.22) |
| 17:57141769-Del | 17 | 57141769 | A/- | rs367700401 | *TRIM37* | 0.254 | VUS | 0.36 | 315 | 0.97  (0.92–1.01) | 122 | 1.04  (0.93–1.16) | — | — |
| 1:78392446-SNV | 1 | 78392446 | G/A | rs1166698 | *NEXN* | 0.182 | B | 0.51 | 280 | 1.02  (0.96–1.09) | 140 | 0.91  (0.84–0.98) | 17 | 1.01  (0.89–1.14) |
| 3:43743914-SNV | 3 | 43743914 | G/T | rs148743497 | *ABHD5* | 7.77×10^–3^ | B | 0.33 | 413 | 0.99  (0.95–1.04) | 24 | 0.89  (0.72–1.07) | — | — |
| 19:44605324-SNV | 19 | 44605324 | A/G | rs118149610 | *ZNF224* | 0.014 | B | 0.79 | 415 | 0.98  (0.93–1.03) | 22 | 1.16  (0.98–1.33) | — | —  — |
| 17:28548810-SNV | 17 | 28548810 | C/G | rs6355 | *SLC6A4* | 0.012 | B | 0.28 | 423 | 0.99  (0.94–1.03) | 14 | 1.00  (0.87–1.13) | — | — |
| 3:186443018-SNV | 3 | 186443018 | T/C | rs1656922 | *KNG1* | 0.537 | B | 0.56 | 146 | 1.06  (0.97–1.15) | 164 | 0.99  (0.91–1.06) | 127 | 0.91  (0.84–0.97) |
| 12:56848079-SNV | 12 | 56848079 | C/T | rs74641138 | *MIP* | 0.024 | B | 0.29 | 421 | 0.94  (0.94–1.04) | 16 | 0.93  (0.72–1.13) | — | — |
| 6:161006105-SNV | 6 | 161006105 | C/T | rs41272112 | *LPA* | 9.53×10^–3^ | B | 0.35 | 412 | 0.99  (0.95–1.04) | 25 | 0.89  (0.73–1.05) | — | — |

Abbreviations: Chr, chromosome; AAF, alternative allele frequency; ACMG, American College of Medical Genetics and Genomics; B, benign; LB, likely benign; VUS, variant of uncertain significance; BF(m), Bayes factor; Ref, reference allele; Alt, alternative allele; N, number of observations; 95% Cred. I, 95% credible interval.

* Genomic positions are reported according to GRCh37.

# Supplementary Table S6. Results of the Bayesian analysis of variance for the association between the 11 cross-validated genetic variants and transferrin saturation

| Variant | Chr | Position | Ref/Alt | dbSNP  ID | Gene | AAF | ACMG | BF(m) | Ref-Ref  (AA) | | Ref-Alt  (AB) | | Alt-Alt  (BB) | |
| --- | --- | --- | --- | --- | --- | --- | --- | --- | --- | --- | --- | --- | --- | --- |
|  |  |  |  |  |  |  |  |  | **N** | **Mean, Transferrin saturation (%)**  **(95% Cred. I)** | **N** | **Mean, Transferrin saturation (%)**  **(95% Cred. I)** | **N** | **Mean, Transferrin saturation (%)**  **(95% Cred. I)** |
| 3:124627024-SNV | 3 | 124627024 | T/G | rs1127233 | *MUC13* | 0.239 | B | 376 | 217 | 27  (26–29) | 139 | 27  (24–29) | 32 | 39  (32–45) |
| 1:91404256-SNV | 1 | 91404256 | C/A | rs41286761 | *ZNF644* | 0.022 | B | 0.89 | 324 | 27  (26–29) | 64 | 31  (28–34) | — | — |
| 3:176765269-SNV | 3 | 176765269 | T/C | rs3749234 | *TBL1XR1* | 0.211 | B | 0.09 | 257 | 28  (26–29) | 113 | 29  (27–31) | 18 | 26  (21–32) |
| 17:57141769-Del | 17 | 57141769 | A/- | rs367700401 | *TRIM37* | 0.254 | VUS | 0.14 | 286 | 28  (26–29) | 102 | 29  (25–32) | — | — |
| 1:78392446-SNV | 1 | 78392446 | G/A | rs1166698 | *NEXN* | 0.182 | B | 0.43 | 243 | 29  (27–31) | 130 | 26  (24–28) | 15 | 31  (26–36) |
| 3:43743914-SNV | 3 | 43743914 | G/T | rs148743497 | *ABHD5* | 7.77×10^–3^ | B | 0.39 | 368 | 28  (27–30) | 20 | 25  (19–31) | — | — |
| 19:44605324-SNV | 19 | 44605324 | A/G | rs118149610 | *ZNF224* | 0.014 | B | 0.55 | 368 | 28  (26–29) | 20 | 32  (27–37) | — | — |
| 17:28548810-SNV | 17 | 28548810 | C/G | rs6355 | *SLC6A4* | 0.012 | B | 0.28 | 374 | 28  (27–29) | 14 | 29  (24–33) | — | — |
| 3:186443018-SNV | 3 | 186443018 | T/C | rs1656922 | *KNG1* | 0.537 | B | 0.57 | 124 | 30  (27–32) | 148 | 29  (26–31) | 116 | 25  (23–27) |
| 12:56848079-SNV | 12 | 56848079 | C/T | rs74641138 | *MIP* | 0.024 | B | 0.27 | 373 | 28  (27–29) | 15 | 28  (21–34) | — | — |
| 6:161006105-SNV | 6 | 161006105 | C/T | rs41272112 | *LPA* | 9.53×10^–3^ | B | 0.31 | 363 | 28  (26–29) | 25 | 30  (23–38) | — | — |

Abbreviations: Chr, chromosome; AAF, alternative allele frequency; ACMG, American College of Medical Genetics and Genomics; B, benign; LB, likely benign; VUS, variant of uncertain significance; BF(m), Bayes factor; Ref, reference allele; Alt, alternative allele; N, number of observations; 95% Cred. I, 95% credible interval.

* Genomic positions are reported according to GRCh37.

# Supplementary Table S7. Results of the Bayesian analysis of variance for the association between the 11 cross-validated genetic variants and plasma transferrin concentration

| Variant | Chr | Position | Ref/Alt | dbSNP  ID | Gene | AAF | ACMG | BF(m) | Ref-Ref  (AA) | | Ref-Alt  (AB) | | Alt-Alt  (BB) | |
| --- | --- | --- | --- | --- | --- | --- | --- | --- | --- | --- | --- | --- | --- | --- |
|  |  |  |  |  |  |  |  |  | **N** | **Mean,**  **Transferrin (g/L)**  **(95% Cred. I)** | **N** | **Mean,**  **Transferrin (g/L)**  **(95% Cred. I)** | **N** | **Mean,**  **Transferrin (g/L)**  **(95% Cred. I)** |
| 3:124627024-SNV | 3 | 124627024 | T/G | rs1127233 | *MUC13* | 0.239 | B | 0.06 | 217 | 2.62  (2.55–2.69) | 140 | 2.58  (2.49–2.66) | 32 | 2.55  (2.41–2.69) |
| 1:91404256-SNV | 1 | 91404256 | C/A | rs41286761 | *ZNF644* | 0.022 | B | 0.15 | 325 | 2.60  (2.55–2.65) | 64 | 2.60  (2.48–2.72) | — | — |
| 3:176765269-SNV | 3 | 176765269 | T/C | rs3749234 | *TBL1XR1* | 0.211 | B | 0.08 | 257 | 2.61  (2.55–2.67) | 114 | 2.56  (2.47–2.65) | 18 | 2.65  (2.41–2.88) |
| 17:57141769-Del | 17 | 57141769 | A/- | rs367700401 | *TRIM37* | 0.254 | VUS | 0.47 | 287 | 2.62  (2.57–2.68) | 102 | 2.53  (2.42–2.64) | — | — |
| 1:78392446-SNV | 1 | 78392446 | G/A | rs1166698 | *NEXN* | 0.182 | B | 0.11 | 243 | 2.59  (2.53–2.65) | 131 | 2.63  (2.54–2.72) | 15 | 2.45  (2.22–2.69) |
| 3:43743914-SNV | 3 | 43743914 | G/T | rs148743497 | *ABHD5* | 7.77×10^–3^ | B | 0.31 | 368 | 2.59  (2.54–2.64) | 21 | 2.68  (2.46–2.90) | — | — |
| 19:44605324-SNV | 19 | 44605324 | A/G | rs118149610 | *ZNF224* | 0.014 | B | 0.24 | 369 | 2.60  (2.55–2.65) | 20 | 2.58  (2.43–2.73) | — | — |
| 17:28548810-SNV | 17 | 28548810 | C/G | rs6355 | *SLC6A4* | 0.012 | B | 0.30 | 375 | 2.60  (2.55–2.65) | 14 | 2.54  (2.36–2.73) | — | — |
| 3:186443018-SNV | 3 | 186443018 | T/C | rs1656922 | *KNG1* | 0.537 | B | 0.21 | 125 | 2.61  (2.51–2.70) | 148 | 2.54  (2.46–2.62) | 116 | 2.67  (2.58–2.75) |
| 12:56848079-SNV | 12 | 56848079 | C/T | rs74641138 | *MIP* | 0.024 | B | 0.31 | 374 | 2.60  (2.55–2.65) | 15 | 2.53  (2.35–2.70) | — | — |
| 6:161006105-SNV | 6 | 161006105 | C/T | rs41272112 | *LPA* | 9.53×10^–3^ | B | 0.46 | 364 | 2.61  (2.56–2.66) | 25 | 2.48  (2.30–2.65) | — | — |

Abbreviations: Chr, chromosome; AAF, alternative allele frequency; ACMG, American College of Medical Genetics and Genomics; B, benign; LB, likely benign; VUS, variant of uncertain significance; BF(m), Bayes factor; Ref, reference allele; Alt, alternative allele; N, number of observations; 95% Cred. I, 95% credible interval.

* Genomic positions are reported according to GRCh37.

# Supplementary Table S8. Results of the Bayesian analysis of variance for the association between the 11 cross-validated genetic variants and body mass index

| Variant | Chr | Position | Ref/Alt | dbSNP  ID | Gene | AAF | ACMG | BF(m) | Ref-Ref  (AA) | | Ref-Alt  (AB) | | Alt-Alt  (BB) | |
| --- | --- | --- | --- | --- | --- | --- | --- | --- | --- | --- | --- | --- | --- | --- |
|  |  |  |  |  |  |  |  |  | **N** | **Mean,**  **Body Mass Index (kg/m^2^)**  **(95% Cred. I)** | **N** | **Mean,**  **Body Mass Index (kg/m^2^)**  **(95% Cred. I)** | **N** | **Mean,**  **Body Mass Index (kg/m^2^)**  **(95% Cred. I)** |
| 3:124627024-SNV | 3 | 124627024 | T/G | rs1127233 | *MUC13* | 0.239 | B | 0.07 | 231 | 25.2  (24.5–25.8) | 146 | 25.0  (24.1–26.0) | 35 | 26.2  (24.8–27.7) |
| 1:91404256-SNV | 1 | 91404256 | C/A | rs41286761 | *ZNF644* | 0.022 | B | 1 | 357 | 25.0  (24.5–25.5) | 55 | 26.6  (24.9–28.3) | — | — |
| 3:176765269-SNV | 3 | 176765269 | T/C | rs3749234 | *TBL1XR1* | 0.211 | B | 0.06 | 253 | 25.2  (24.6–25.9) | 141 | 25.3  (24.6–26.0) | 18 | 24.3  (21.0–27.6) |
| 17:57141769-Del | 17 | 57141769 | A/- | rs367700401 | *TRIM37* | 0.254 | VUS | 0.15 | 297 | 25.1  (24.5–25.7) | 115 | 25.5  (24.7–26.3) | — | — |
| 1:78392446-SNV | 1 | 78392446 | G/A | rs1166698 | *NEXN* | 0.182 | B | 1 | 269 | 25.7  (25.1–26.3) | 127 | 24.3  (23.3–25.2) | 16 | 24.9  (23.3–26.6) |
| 3:43743914-SNV | 3 | 43743914 | G/T | rs148743497 | *ABHD5* | 7.77×10^–3^ | B | 0.93 | 398 | 25.1  (24.6–25.6) | 14 | 27.6  (26.0–29.1) | — | — |
| 19:44605324-SNV | 19 | 44605324 | A/G | rs118149610 | *ZNF224* | 0.014 | B | 0.24 | 392 | 25.2  (24.7–25.7) | 20 | 24.9  (22.5–27.4) | — | — |
| 17:28548810-SNV | 17 | 28548810 | C/G | rs6355 | *SLC6A4* | 0.012 | B | 0.32 | 395 | 25.2  (24.7–25.7) | 17 | 26.1  (25–27.2) | — | — |
| 3:186443018-SNV | 3 | 186443018 | T/C | rs1656922 | *KNG1* | 0.537 | B | 1 | 153 | 25.6  (24.8–26.4) | 145 | 24.3  (23.5–25.1) | 114 | 25.9  (24.9–27.0) |
| 12:56848079-SNV | 12 | 56848079 | C/T | rs74641138 | *MIP* | 0.024 | B | 0.29 | 398 | 25.2  (24.7–25.7) | 14 | 25.7  (24.7–26.6) | — | — |
| 6:161006105-SNV | 6 | 161006105 | C/T | rs41272112 | *LPA* | 9.53×10^–3^ | B | 0.38 | 387 | 25.1  (24.6–25.7) | 25 | 26.4  (25.0–27.7) | — | — |

Abbreviations: Chr, chromosome; AAF, alternative allele frequency; ACMG, American College of Medical Genetics and Genomics; B, benign; LB, likely benign; VUS, variant of uncertain significance; BF(m), Bayes factor; Ref, reference allele; Alt, alternative allele; N, number of observations; 95% Cred. I, 95% credible interval; BMI, body mass index.

* Genomic positions are reported according to GRCh37.

# Supplementary Table S9. Results of the Bayesian analysis of variance for the association between the 11 cross-validated genetic variants and alanine aminotransferase

| Variant | Chr | Position | Ref/Alt | dbSNP  ID | Gene | AAF | ACMG | BF(m) | Ref-Ref  (AA) | | Ref-Alt  (AB) | | Alt-Alt  (BB) | |
| --- | --- | --- | --- | --- | --- | --- | --- | --- | --- | --- | --- | --- | --- | --- |
|  |  |  |  |  |  |  |  |  | **N** | **Mean,**  **ALAT (UI/L)**  **(95% Cred. I)** | **N** | **Mean,**  **ALAT (UI/L)**  **(95% Cred. I)** | **N** | **Mean,**  **ALAT (UI/L)**  **(95% Cred. I)** |
| 3:124627024-SNV | 3 | 124627024 | T/G | rs1127233 | *MUC13* | 0.239 | B | 7 | 218 | 46  (41–50) | 140 | 36  (31–40) | 30 | 55  (37.6–71) |
| 1:91404256-SNV | 1 | 91404256 | C/A | rs41286761 | *ZNF644* | 0.022 | B | 5 | 324 | 41  (37–44) | 64 | 53  (43–64) | — | — |
| 3:176765269-SNV | 3 | 176765269 | T/C | rs3749234 | *TBL1XR1* | 0.211 | B | 0.17 | 243 | 44  (40–49) | 128 | 41  (35–47) | 17 | 31  (23–39) |
| 17:57141769-Del | 17 | 57141769 | A/- | rs367700401 | *TRIM37* | 0.254 | VUS | 0.13 | 280 | 43  (39–47) | 108 | 43  (36–49) | — | — |
| 1:78392446-SNV | 1 | 78392446 | G/A | rs1166698 | *NEXN* | 0.182 | B | 0.84 | 250 | 45  (41–50) | 121 | 37  (31–42) | 17 | 49  (35–63) |
| 3:43743914-SNV | 3 | 43743914 | G/T | rs148743497 | *ABHD5* | 7.77×10^–3^ | B | 0.23 | 364 | 43  (39–46) | 24 | 40  (31–49) | — | — |
| 19:44605324-SNV | 19 | 44605324 | A/G | rs118149610 | *ZNF224* | 0.014 | B | 0.23 | 365 | 43  (39–46) | 23 | 43  (34–51) | — | — |
| 17:28548810-SNV | 17 | 28548810 | C/G | rs6355 | *SLC6A4* | 0.012 | B | 0.26 | 372 | 43  (39–46) | 16 | 42  (30–53) | — | — |
| 3:186443018-SNV | 3 | 186443018 | T/C | rs1656922 | *KNG1* | 0.537 | B | 0.16 | 139 | 40  (35–46) | 133 | 41  (35–46) | 116 | 48  (41–55) |
| 12:56848079-SNV | 12 | 56848079 | C/T | rs74641138 | *MIP* | 0.024 | B | 0.26 | 372 | 43  (39–46) | 16 | 44  (27–60) | — | — |
| 6:161006105-SNV | 6 | 161006105 | C/T | rs41272112 | *LPA* | 9.53×10^–3^ | B | 4 | 367 | 42  (38–45) | 21 | 61  (42–79) | — | — |

Abbreviations: Chr, chromosome; AAF, alternative allele frequency; ACMG, American College of Medical Genetics and Genomics; B, benign; LB, likely benign; VUS, variant of uncertain significance; BF(m), Bayes factor; Ref, reference allele; Alt, alternative allele; N, number of observations; 95% Cred. I, 95% credible interval; ALAT, alanine aminotransferase.

* Genomic positions are reported according to GRCh37.

# Supplementary Table S10. Results of the Bayesian analysis of variance for the association between the 11 cross-validated genetic variants and C-reactive protein

| Variant | Chr | Position | Ref/Alt | dbSNP  ID | Gene | AAF | ACMG | BF(m) | Ref-Ref  (AA) | | Ref-Alt  (AB) | | Alt-Alt  (BB) | |
| --- | --- | --- | --- | --- | --- | --- | --- | --- | --- | --- | --- | --- | --- | --- |
|  |  |  |  |  |  |  |  |  | **N** | **Mean,**  **CRP (mg/L)**  **(95% Cred. I)** | **N** | **Mean,**  **CRP (mg/L)**  **(95% Cred. I)** | **N** | **Mean,**  **CRP (mg/L)**  **(95% Cred. I)** |
| 3:124627024-SNV | 3 | 124627024 | T/G | rs1127233 | *MUC13* | 0.239 | B | 0.09 | 163 | 2.8  (2.5–3.1) | 105 | 2.8  (2.4–3.3) | 13 | 3.4  (2.4–4.5) |
| 1:91404256-SNV | 1 | 91404256 | C/A | rs41286761 | *ZNF644* | 0.022 | B | 0.27 | 247 | 2.9  (2.6–3.2) | 34 | 2.5  (1.7–3.4) | — | — |
| 3:176765269-SNV | 3 | 176765269 | T/C | rs3749234 | *TBL1XR1* | 0.211 | B | 3 | 176 | 3.1  (2.8–3.5) | 94 | 2.3  (1.9–2.7) | 11 | 2.7  (1.4–4) |
| 17:57141769-Del | 17 | 57141769 | A/- | rs367700401 | *TRIM37* | 0.254 | VUS | 0.23 | 211 | 2.9  (2.6–3.2) | 70 | 2.6  (2.2–3.1) | — | — |
| 1:78392446-SNV | 1 | 78392446 | G/A | rs1166698 | *NEXN* | 0.182 | B | 0.08 | 183 | 2.8  (2.5–3.1) | 88 | 2.9  (2.3–3.4) | 10 | 3.1  (1.3–4.9) |
| 3:43743914-SNV | 3 | 43743914 | G/T | rs148743497 | *ABHD5* | 7.77×10^–3^ | B | 35 | 263 | 3.0  (2.7–3.2) | 18 | 1.2  (0.5–1.8) | — | — |
| 19:44605324-SNV | 19 | 44605324 | A/G | rs118149610 | *ZNF224* | 0.014 | B | 4 | 262 | 2.9  (2.7–3.2) | 19 | 1.6  (0.6–2.7) | — | — |
| 17:28548810-SNV | 17 | 28548810 | C/G | rs6355 | *SLC6A4* | 0.012 | B | 0.33 | 268 | 2.9  (2.6–3.1) | 13 | 2.5  (1.6–3.4) | — | — |
| 3:186443018-SNV | 3 | 186443018 | T/C | rs1656922 | *KNG1* | 0.537 | B | 0.05 | 95 | 2.8  (2.3–3.2) | 97 | 2.8  (2.3–3.2) | 89 | 3.0  (2.5–3.5) |
| 12:56848079-SNV | 12 | 56848079 | C/T | rs74641138 | *MIP* | 0.024 | B | 0.42 | 273 | 2.9  (2.6–3.1) | 8 | 2.3  (1.0–3.6) | — | — |
| 6:161006105-SNV | 6 | 161006105 | C/T | rs41272112 | *LPA* | 9.53×10^–3^ | B | 0.30 | 269 | 2.8  (2.6–3.1) | 12 | 2.7  (1.5–3.9) | — | — |

Abbreviations: Chr, chromosome; AAF, alternative allele frequency; ACMG, American College of Medical Genetics and Genomics; B, benign; LB, likely benign; VUS, variant of uncertain significance; BF(m), Bayes factor; Ref, reference allele; Alt, alternative allele; N, number of observations; 95% Cred. I, 95% credible interval; CRP, C-reactive protein.

* Genomic positions are reported according to GRCh37.

# Supplementary Table S11. Baseline characteristics of patients with unexplained hyperferritinemia in the replication cohort

| Variable | No. of patients | Value |
| --- | --- | --- |
| Age, y, median (25th–75th percentile) | 263 | 66 (55–74) |
| Male sex, No. (%) (95% CI) | 263 | 203 (77.2) (72.1–82.3) |
| Female sex, No. (%) (95% CI) | 263 | 59 (22.4) (17.4–27.5) |
| Ferritin, µg/L, median (25th–75th percentile) | 263 | 935 (612–1350) |
| Transferrin saturation, %, median (25th–75th percentile) | 222 | 55 (39–74) |
| Transferrin saturation >45%, No. (%) (95% CI) | 222 | 151 (68.0) (61.8–74.2) |
| Transferrin saturation >55%, No. (%) (95% CI) | 222 | 105 (47.3) (40.7–53.9) |
| Transferrin saturation >60%, No. (%) (95% CI) | 222 | 85 (38.3) (31.8–44.7) |
| MRI-derived liver iron, µmol/g, median (25th–75th percentile) | 98 | 120 (70–190) |
| Liver iron >36 µmol/g, No. (%) (95% CI) | 98 | 95 (96.9) (93.5–100) |
| Liver iron >80 µmol/g, No. (%) (95% CI) | 98 | 69 (70.4) (61.2–79.6) |
| Liver iron >160 µmol/g, No. (%) (95% CI) | 98 | 33 (33.7) (24.1–43.2) |
| *MUC13* p.Arg503Ser genotype* |  |  |
| Arg/Arg, No. (%) (95% CI) | 263 | 140 (53.2) (47.2–59.3) |
| Arg/Ser, No. (%) (95% CI) | 263 | 95 (36.1) (30.3–42.0) |
| Ser/Ser, No. (%) (95% CI) | 263 | 28 (10.6) (6.9–14.4) |

Abbreviations: CI, confidence interval; MRI, magnetic resonance imaging.

*Genotype frequencies were tested for Hardy-Weinberg equilibrium; no significant deviation was observed (P = 0.07).

# Supplementary Table S12. Distribution of genetic, demographic, and biochemical characteristics according to transferrin saturation in the replication cohort

| Variable | Transferrin saturation ≤55% (n=117),  % (95% CI) | Transferrin saturation >55% (n=105),  % (95% CI) |
| --- | --- | --- |
| *MUC13* p.Arg503Ser genotype |  |  |
| Arg/Arg | 55.6 (46.4–64.7) | 50.5 (40.8–60.2) |
| Arg/Ser | 40.2 (31.2–49.2) | 34.3 (25.1–43.5) |
| Ser/Ser | 4.3 (0.6–8.0) | 15.2 (8.3–22.2) |
| Sex |  |  |
| Female | 17.1 (10.2–24.0) | 29.5 (20.7–38.4) |
| Male | 82.9 (76.0–89.8) | 70.5 (61.6–79.3) |
| Ferritin >1500 µg/L | 16.2 (9.5–23.0) | 27.6 (18.9–36.3) |
| Age strata |  |  |
| <40 y | 3.4 (0.1–6.8) | 9.5 (3.8–15.2) |
| 40–59 y | 27.4 (19.2–35.5) | 27.6 (18.9–36.3) |
| ≥60 y | 69.2 (60.7–77.7) | 62.9 (53.5–72.3) |

Abbreviation: CI, confidence interval.

Values are expressed as percentage (95% CI).

# Supplementary Table S13. Multivariable logistic regression analysis of predictors of elevated transferrin saturation (greater than 55%) in the replication cohort

| Variable | Odds ratio | 95% CI | *P* value |
| --- | --- | --- | --- |
| *MUC13* p.Arg503Ser Ser/Ser genotype | 4.52 | 1.55–13.19 | 0.006 |
| Female sex | 2.37 | 1.21–4.63 | 0.012 |
| Ferritin >1500 µg/L | 2.30 | 1.16–4.56 | 0.017 |
| Age (per year) | 0.98 | 0.96–1.00 | 0.079 |

Abbreviation: CI, confidence interval.

Model performance: Nagelkerke R² = 0.13; Hosmer-Lemeshow goodness-of-fit test *P* = 0.26; area under the receiver operating characteristic curve = 0.66 (95% CI, 0.59-0.72).

# Supplementary Table S14. Assessment of iron metabolism markers and liver histopathology in *Muc13*^−/−^ and *Muc13*^+/+^ mice

|  | *Muc13^+/+^* mice  (n = 7) | *Muc13^–/–^* mice  (n = 6) | *P-value* |
| --- | --- | --- | --- |
| Iron (µmol/L) — median (IQR, 25^th^–75^th^) | 22.2 (21.6–25.6) | 17.2 (15.8–20.0) | 0.03 |
| Transferrin saturation (%) — median (IQR, 25^th^–75^th^) | 22.1 (19.8–22.4) | 15.9 (14.9–17.8) | 0.02 |
| Transferrin (g/L) — median (IQR, 25^th^–75^th^) | 0.80 (0.75–0.80) | 0.70 (0.70–0.78) | 0.55 |
| Ferritin (µg/L) — median (IQR, 25^th^–75^th^) | 614.0 (358.6–742.0) | 624.9 (441.6–724.9) | 0.89 |
| Hepcidin (ng/mL) — median (IQR, 25^th^–75^th^) | 15.80 (7.60–20.65) | 11.85 (4.28–37.2) | 0.77 |
| Hepcidin ≤ 4.5 ng/mL — % (n/N) | 0% (0/7) | 50% (3/6) | 0.07 |
| Iron in liver (µmol/L) — median (IQR, 25^th^–75^th^) | 2.40 (1.75–3.75) | 1.20 (0.73–2.35) | 0.53 |

Abbreviations: IQR, interquartile range.

Values are expressed as median (IQR, 25th-75th percentile). Comparisons were performed using the Mann-Whitney U test.

# Supplementary Table S15. High-confidence protein candidates identified in association with *MUC13* overexpression using Ingenuity Pathway Analysis

Raw data are provided in the accompanying Excel file “Supplementary Table S15”.

Analysis was performed using Ingenuity Pathway Analysis (IPA; QIAGEN Inc., https://digitalinsights.qiagen.com/IPA).

# Supplementary Table S16. Proteins associated with the integrin signaling pathway identified by Ingenuity Pathway Analysis

| Symbol | Entrez Gene Name | Entrez Gene | Expr Fold Change | Expected | Location | Type(s) |
| --- | --- | --- | --- | --- | --- | --- |
| ARPC5 | actin related protein 23 complex subunit 5 | 10092 | 100 | Up | Cytoplasm | other |
| ARPC1A | actin related protein 23 complex subunit 1A | 10552 | 100 | Up | Extracellular Space | other |
| ARPC5L | actin related protein 23 complex subunit 5 like | 81873 | 100 | Up | Cytoplasm | other |
| PFN2 | profilin 2 | 5217 | 100 | Up | Cytoplasm | enzyme |
| RAC3 | Rac family small GTPase 3 | 5881 | 100 | Up | Cytoplasm | enzyme |
| RRAS2 | RAS related 2 | 22800 | 100 | Up | Plasma Membrane | enzyme |
| ITGA6 | **integrin subunit alpha 6** | **3655** | **46.423** | **Up** | **Plasma Membrane** | **transmembrane receptor** |
| ITGB4 | **integrin subunit beta 4** | **3691** | **38.328** | **Up** | **Plasma Membrane** | **transmembrane receptor** |
| VASP | vasodilator stimulated phosphoprotein | 7408 | 25.611 | Down | Plasma Membrane | other |
| MPRIP | myosin phosphatase Rho interacting protein | 23164 | 14.545 |  | Cytoplasm | other |
| RAP2A | RAP2A, member of RAS oncogene family | 5911 | 13.729 | Up | Plasma Membrane | enzyme |
| PPP1R12A | protein phosphatase 1 regulatory subunit 12A | 4659 | 9.934 |  | Cytoplasm | phosphatase |
| ARPC2 | actin related protein 23 complex subunit 2 | 10109 | 5.38 | Up | Cytoplasm | other |
| ARPC3 | actin related protein 23 complex subunit 3 | 10094 | 3.917 | Up | Cytoplasm | other |
| ACTR2 | actin related protein 2 | 10097 | 3.474 | Up | Plasma Membrane | other |
| ARPC4 | actin related protein 23 complex subunit 4 | 10093 | 2.807 | Up | Cytoplasm | other |
| MYL12B | myosin light chain 12B | 103910 | 2.374 | Up | Cytoplasm | other |
| ACTA1 | actin alpha 1, skeletal muscle | 58 | 2.307 | Up | Cytoplasm | other |
| ARPC1B | actin related protein 23 complex subunit 1B | 10095 | 2.277 | Up | Cytoplasm | other |
| ARF3 | ADP ribosylation factor 3 | 377 | 2.23 | Down | Cytoplasm | enzyme |
| ACTG1 | actin gamma 1 | 71 | 2.187 | Up | Cytoplasm | other |
| ACTN1 | actinin alpha 1 | 87 | 2.179 | Up | Cytoplasm | transcription regulator |
| ACTR3 | actin related protein 3 | 10096 | 2.173 | Up | Plasma Membrane | other |
| PPP1CB | protein phosphatase 1 catalytic subunit beta | 5500 | 2.154 |  | Cytoplasm | phosphatase |
| ACTN4 | actinin alpha 4 | 81 | 1.506 | Up | Cytoplasm | transcription regulator |
| CDC42 | cell division cycle 42 | 998 | -1.057 | Up | Cytoplasm | enzyme |

Analysis was performed using Ingenuity Pathway Analysis (IPA; QIAGEN Inc., https://digitalinsights.qiagen.com/IPA).

Bold text indicates integrin subunits (ITGA6 and ITGB4).

**SUPPLEMENTARY FIGURES**

# Supplementary Figure S1. Flow diagram of patient selection

# Supplementary Figure S2. Principal component analysis according to patient ancestry

Ancestry inference was performed using principal component analysis (PCA) on study samples merged with 1092 subjects from the 1000 Genomes Project Phase 1 super populations as reference populations: Africans (AFR, n = 246), Americans (AMR, n = 181), Asians (ASN, n = 286), and Europeans (EUR, n = 379). Data were retrieved from http://www.internationalgenome.org/data/.

# Supplementary Figure S3. The exome association study identified several genes associated with plasma ferritin levels.

Bayesian analysis of variance reporting the association between the 11 cross-validated genetic variants and plasma ferritin concentration (µg/L). The Bonferroni-corrected *P* value, the Bayes factor (BF[m]), and the HGNC-approved gene symbol are shown at the top of each panel. For each variant, we reported the mean and the 95% credible interval (95% Cr.I) for each genotype subgroup. As a metric of Bayesian inference, the Bayes factor (BF[m]) is a natural ratio to compare the marginal likelihoods of a null and an alternative hypothesis. AA: homozygous genotype for the reference allele; AB: heterozygous genotype; BB: homozygous genotype for the alternative allele.

# Supplementary Figure S4. Results of the Bayesian analysis of variance for the association between the 11 cross-validated genetic variants and plasma iron concentration

The Bonferroni-corrected *P* value, the Bayes factor [BF(m)], and the HGNC-approved gene symbol are reported at the top of each panel. For each variant, the mean and 95% credible interval (95% Cred. I) per genotype subgroup are shown. The Bayes factor [BF(m)], as a metric of Bayesian inference, constitutes a natural ratio to compare the marginal likelihoods between the null and alternative hypotheses. AA, homozygous genotype for the reference allele; AB, heterozygous genotype; BB, homozygous genotype for the alternative allele.

# Supplementary Figure S5. Results of the Bayesian analysis of variance for the association between the 11 cross-validated genetic variants and transferrin saturation

The Bonferroni-corrected *P* value, the Bayes factor [BF(m)], and the HGNC-approved gene symbol are reported at the top of each panel. For each variant, the mean and 95% credible interval (95% Cred. I) per genotype subgroup are shown. The Bayes factor [BF(m)], as a metric of Bayesian inference, constitutes a natural ratio to compare the marginal likelihoods between the null and alternative hypotheses. AA, homozygous genotype for the reference allele; AB, heterozygous genotype; BB, homozygous genotype for the alternative allele.

# Supplementary Figure S6. Results of the Bayesian analysis of variance for the association between the 11 cross-validated genetic variants and plasma transferrin concentration

The Bonferroni-corrected *P* value, the Bayes factor [BF(m)], and the HGNC-approved gene symbol are reported at the top of each panel. For each variant, the mean and 95% credible interval (95% Cred. I) per genotype subgroup are shown. The Bayes factor [BF(m)], as a metric of Bayesian inference, constitutes a natural ratio to compare the marginal likelihoods between the null and alternative hypotheses. AA, homozygous genotype for the reference allele; AB, heterozygous genotype; BB, homozygous genotype for the alternative allele.

# Supplementary Figure S7. Representative immunohistochemistry images of small intestine sections from *Muc13*^+/+^ and *Muc13*^−/−^ mice stained with anti-MUC13 antibody

# Supplementary Figure S8. Assessment of liver histopathology in *Muc13*^−/−^ and *Muc13*^+/+^ mice

Immunohistochemistry confirmed the reduced expression of Muc13 in *Muc13*^−/−^ liver samples (A, B). Hematoxylin-eosin-saffron staining (C, D). Perls Prussian blue (E, F) and Sirius red (G, H) staining did not reveal significant differences between *Muc13*^−/−^ and *Muc13*^+/+^ liver sections, suggesting the absence of iron deposition or significant liver fibrosis in *Muc13*^−/−^ mice.

# Supplementary Figure S9. Co-immunoprecipitation analysis of the interaction between *MUC13* and calreticulin

(A) Schematic diagram of the co-immunoprecipitation procedure. (B) LS513 cells were transfected with *MUC13* or control siRNA for 48 hours and subsequently treated for 72 hours with 320 μg/mL iron sucrose. Co-immunoprecipitation (co-IP) analysis was performed to assess the association between MUC13 and calreticulin. (C) Control and *MUC13*-overexpressing SW480 cells were treated for 72 hours with 320 μg/mL iron sucrose. Immunoprecipitation analysis was performed to evaluate the association between MUC13 and calreticulin.

# Supplementary Figure S10. Ingenuity Pathway Analysis of *MUC13*-associated proteins

Ingenuity Pathway Analysis (IPA) was performed to examine the proteins present exclusively in MUC13-overexpressing SW480 cells. IPA is based on the QIAGEN Knowledge Base (QIAGEN Inc., https://digitalinsights.qiagen.com/IPA), an extensive repository of biological interactions between proteins, RNAs, genes, isoforms, metabolites, complexes, cells, tissues, drugs, and diseases, manually curated by experts based on more than 3.58 million published studies.

# Supplementary Figure S11. *MUC13* promotes ferric iron uptake and increases ferritin heavy chain expression in response to iron sucrose treatment in intestinal epithelial cells

Panels A and B represent the continuation of those presented in Figure 4. (A) LS513 cells were transfected with *MUC13* or control siRNA for 48 hours and subsequently treated for 6 hours with the indicated concentrations of iron sucrose (Fe^3+^). Western blot analysis was performed on LS513 cell lysates for FTH1 (n = 2). (B) Control and *MUC13*-overexpressing SW480 cells were treated for 6 hours with the indicated concentrations of iron sucrose (Fe^3+^). Western blot analysis was performed on SW480 cell lysates for FTH1 (n = 2).

# Supplementary Figure S12. Effect of the *MUC13* p.Arg503Ser variant on ferritin heavy chain expression in response to iron treatment

Vector control, *MUC13*-overexpressing wild-type (WT), and *MUC13*-overexpressing mutant (p.Arg503Ser, MUT) SW480 human intestinal epithelial cells were either untreated or treated for 6 hours with 320 μg/mL iron sucrose (Fe^3+^). Western blot analysis was performed on SW480 cell lysates (n = 2) with the indicated antibodies.

# Extended references for the mucin-integrin-calreticulin/mobilferrin pathway

The following references provide evidence for the role of the mucin-integrin-calreticulin/mobilferrin pathway in intestinal ferric iron absorption ^37-61^.

# REFERENCES

1.	Alix T, Chery C, Josse T, et al. Predictors of the utility of clinical exome sequencing as a first-tier genetic test in patients with Mendelian phenotypes: results from a referral center study on 603 consecutive cases. *Hum Genomics.* 2023;17(1):5.

2.	Mergnac JP, Wiedemann A, Chery C, et al. Diagnostic yield of clinical exome sequencing as a first-tier genetic test for the diagnosis of genetic disorders in pediatric patients: results from a referral center study. *Hum Genet.* 2022;141(7):1269-1278.

3.	Silva Rodriguez M, Mulot M, Chery C, et al. Exome-based genotype-first reverse phenotyping using structured electronic health record data identifies novel SERPINA1 variants associated with liver markers and demonstrates a dominant effect for specific variants on liver phenotype. *Hepatol Res.* 2025;55(7):1075-1092.

4.	Oussalah A, Jeannesson-Thivisol E, Chery C, et al. Population and evolutionary genetics of the PAH locus to uncover overdominance and adaptive mechanisms in phenylketonuria: Results from a multiethnic study. *EBioMedicine.* 2020;51:102623.

5.	Wiedemann A, Chery C, Coelho D, et al. Mutations in MTHFR and POLG impaired activity of the mitochondrial respiratory chain in 46-year-old twins with spastic paraparesis. *J Hum Genet.* 2020;65(2):91-98.

6.	Mergnac JP, Wiedemann A, Chery C, et al. Diagnostic yield of clinical exome sequencing as a first-tier genetic test for the diagnosis of genetic disorders in pediatric patients: results from a referral center study. *Hum Genet 2021 DOI: 101007/s00439-021-02358-0.* 2021.

7.	Gueant JL, Chery C, Oussalah A, et al. APRDX1 mutant allele causes a MMACHC secondary epimutation in cblC patients. *Nat Commun.* 2018;9(1):67.

8.	Renard E, Chery C, Oussalah A, et al. Exome sequencing of cases with neural tube defects identifies candidate genes involved in one-carbon/vitamin B12 metabolisms and Sonic Hedgehog pathway. *Hum Genet.* 2019;138(7):703-713.

9.	Van der Auwera GA, Carneiro MO, Hartl C, et al. From FastQ data to high confidence variant calls: the Genome Analysis Toolkit best practices pipeline. *Curr Protoc Bioinformatics.* 2013;43:11 10 11-11 10 33.

10.	Schwarz JM, Cooper DN, Schuelke M, Seelow D. MutationTaster2: mutation prediction for the deep-sequencing age. *Nat Methods.* 2014;11(4):361-362.

11.	Shihab HA, Gough J, Cooper DN, et al. Predicting the functional, molecular, and phenotypic consequences of amino acid substitutions using hidden Markov models. *Hum Mutat.* 2013;34(1):57-65.

12.	Dong C, Wei P, Jian X, et al. Comparison and integration of deleteriousness prediction methods for nonsynonymous SNVs in whole exome sequencing studies. *Hum Mol Genet.* 2015;24(8):2125-2137.

13.	Kumar P, Henikoff S, Ng PC. Predicting the effects of coding non-synonymous variants on protein function using the SIFT algorithm. *Nat Protoc.* 2009;4(7):1073-1081.

14.	Choi Y, Chan AP. PROVEAN web server: a tool to predict the functional effect of amino acid substitutions and indels. *Bioinformatics.* 2015;31(16):2745-2747.

15.	Quang D, Chen Y, Xie X. DANN: a deep learning approach for annotating the pathogenicity of genetic variants. *Bioinformatics.* 2015;31(5):761-763.

16.	Richards S, Aziz N, Bale S, et al. Standards and guidelines for the interpretation of sequence variants: a joint consensus recommendation of the American College of Medical Genetics and Genomics and the Association for Molecular Pathology. *Genet Med.* 2015;17(5):405-424.

17.	Landrum MJ, Lee JM, Benson M, et al. ClinVar: improving access to variant interpretations and supporting evidence. *Nucleic Acids Res.* 2018;46(D1):D1062-D1067.

18.	Sheng YH, Lourie R, Linden SK, et al. The MUC13 cell-surface mucin protects against intestinal inflammation by inhibiting epithelial cell apoptosis. *Gut.* 2011;60(12):1661-1670.

19.	Lefebvre T, Dessendier N, Houamel D, et al. LC-MS/MS method for hepcidin-25 measurement in human and mouse serum: clinical and research implications in iron disorders. *Clin Chem Lab Med.* 2015;53(10):1557-1567.

20.	Gracz AD, Puthoff BJ, Magness ST. Identification, isolation, and culture of intestinal epithelial stem cells from murine intestine. *Methods Mol Biol.* 2012;879:89-107.

21.	Sheng YH, Triyana S, Wang R, et al. MUC1 and MUC13 differentially regulate epithelial inflammation in response to inflammatory and infectious stimuli. *Mucosal Immunol.* 2013;6(3):557-568.

22.	Sheng YH, He Y, Hasnain SZ, et al. MUC13 protects colorectal cancer cells from death by activating the NF-kappaB pathway and is a potential therapeutic target. *Oncogene.* 2017;36(5):700-713.

23.	Calvano SE, Xiao W, Richards DR, et al. A network-based analysis of systemic inflammation in humans. *Nature.* 2005;437(7061):1032-1037.

24.	Karczewski KJ, Francioli LC, Tiao G, et al. The mutational constraint spectrum quantified from variation in 141,456 humans. *Nature.* 2020;581(7809):434-443.

25.	Li B, Leal SM. Methods for detecting associations with rare variants for common diseases: application to analysis of sequence data. *Am J Hum Genet.* 2008;83(3):311-321.

26.	Lee Y, Park S, Moon S, et al. On the analysis of a repeated measure design in genome-wide association analysis. *Int J Environ Res Public Health.* 2014;11(12):12283-12303.

27.	Ware EB, Mukherjee B, Sun YV, Diez-Roux AV, Kardia SL, Smith JA. Comparative genome-wide association studies of a depressive symptom phenotype in a repeated measures setting by race/ethnicity in the Multi-Ethnic Study of Atherosclerosis. *BMC Genet.* 2015;16:118.

28.	Ronnegard L, McFarlane SE, Husby A, Kawakami T, Ellegren H, Qvarnstrom A. Increasing the power of genome wide association studies in natural populations using repeated measures - evaluation and implementation. *Methods Ecol Evol.* 2016;7(7):792-799.

29.	Stephens M, Balding DJ. Bayesian statistical methods for genetic association studies. *Nat Rev Genet.* 2009;10(10):681-690.

30.	Xu X, Pennell ML, Lu B, Murray DM. Efficient Bayesian joint models for group randomized trials with multiple observation times and multiple outcomes. *Stat Med.* 2012;31(24):2858-2871.

31.	van den Bergh D, Van Doorn J, Marsman M, et al. A tutorial on conducting and interpreting a Bayesian ANOVA in JASP. *LAnnee psychologique.* 2020;120(1):73-96.

32.	Dittrich D, Leenders RTA, Mulder J. Network autocorrelation modeling: A Bayes factor approach for testing (multiple) precise and interval hypotheses. *Sociological Methods & Research.* 2019;48(3):642-676.

33.	Love J, Selker R, Marsman M, et al. JASP: Graphical statistical software for common statistical designs. *Journal of Statistical Software.* 2019;88(1):1-17.

34.	Blom N, Gammeltoft S, Brunak S. Sequence and structure-based prediction of eukaryotic protein phosphorylation sites. *J Mol Biol.* 1999;294(5):1351-1362.

35.	Blom N, Sicheritz-Ponten T, Gupta R, Gammeltoft S, Brunak S. Prediction of post-translational glycosylation and phosphorylation of proteins from the amino acid sequence. *Proteomics.* 2004;4(6):1633-1649.

36.	Chen M, Zhang W, Gou Y, et al. GPS 6.0: an updated server for prediction of kinase-specific phosphorylation sites in proteins. *Nucleic Acids Res.* 2023;51(W1):W243-W250.

37.	Conrad ME, Umbreit JN, Moore EG, Peterson RD, Jones MB. A newly identified iron binding protein in duodenal mucosa of rats. Purification and characterization of mobilferrin. *J Biol Chem.* 1990;265(9):5273-5279.

38.	Conrad ME, Umbreit JN, Moore EG, Harper KP. Iron absorption via the mucin-integrin mobilferrin pathway. *Trans Assoc Am Physicians.* 1992;105:133-148.

39.	Conrad ME, Umbreit JN, Moore EG, Rodning CR. Newly identified iron-binding protein in human duodenal mucosa. *Blood.* 1992;79(1):244-247.

40.	Conrad ME, Umbreit JN. A concise review: iron absorption--the mucin-mobilferrin-integrin pathway. A competitive pathway for metal absorption. *Am J Hematol.* 1993;42(1):67-73.

41.	Conrad ME, Umbreit JN, Moore EG. Regulation of iron absorption: proteins involved in duodenal mucosal uptake and transport. *J Am Coll Nutr.* 1993;12(6):720-728.

42.	Conrad ME, Umbreit JN, Moore EG. Rat duodenal iron-binding protein mobilferrin is a homologue of calreticulin. *Gastroenterology.* 1993;104(6):1700-1704.

43.	Conrad ME, Umbreit JN, Peterson RD, Moore EG, Harper KP. Function of integrin in duodenal mucosal uptake of iron. *Blood.* 1993;81(2):517-521.

44.	Wolf G, Wessling-Resnick M. An integrin-mobilferrin iron transport pathway in intestine and hematopoietic cells. *Nutr Rev.* 1994;52(11):387-389.

45.	Conrad ME, Umbreit JN, Moore EG, Heiman D. Mobilferrin is an intermediate in iron transport between transferrin and hemoglobin in K562 cells. *J Clin Invest.* 1996;98(6):1449-1454.

46.	Umbreit JN, Conrad ME, Moore EG, Desai MP, Turrens J. Paraferritin: a protein complex with ferrireductase activity is associated with iron absorption in rats. *Biochemistry.* 1996;35(20):6460-6469.

47.	Umbreit JN, Conrad ME, Berry MA, et al. The alternate iron transport pathway: mobilferrin and integrin in reticulocytes. *Br J Haematol.* 1997;96(3):521-529.

48.	Umbreit JN, Conrad ME, Moore EG, Latour LF. Iron absorption and cellular transport: the mobilferrin/paraferritin paradigm. *Semin Hematol.* 1998;35(1):13-26.

49.	Conrad ME, Umbreit JN, Moore EG. Iron absorption and transport. *Am J Med Sci.* 1999;318(4):213-229.

50.	Conrad ME, Umbreit JN. Iron absorption and transport-an update. *Am J Hematol.* 2000;64(4):287-298.

51.	Conrad ME, Umbreit JN, Moore EG, et al. Separate pathways for cellular uptake of ferric and ferrous iron. *Am J Physiol Gastrointest Liver Physiol.* 2000;279(4):G767-774.

52.	Conrad ME, Umbreit JN. Iron absorption: relative importance of iron transport pathways. *Am J Hematol.* 2001;67(3):215.

53.	Núñez MT, Osorio A, Tapia V, Vergara A, Mura CV. Iron-induced oxidative stress up-regulates calreticulin levels in intestinal epithelial (Caco-2) cells. *J Cell Biochem.* 2001;82(4):660-665.

54.	Conrad ME, Umbreit JN. Pathways of iron absorption. *Blood Cells Mol Dis.* 2002;29(3):336-355.

55.	Lee P, Gelbart T, West C, Halloran C, Beutler E. Seeking candidate mutations that affect iron homeostasis. *Blood Cells Mol Dis.* 2002;29(3):471-487.

56.	Simovich MJ, Conrad ME, Umbreit JN, Moore EG, Hainsworth LN, Smith HK. Cellular location of proteins related to iron absorption and transport. *Am J Hematol.* 2002;69(3):164-170.

57.	Umbreit JN, Conrad ME, Hainsworth LN, Simovich M. The ferrireductase paraferritin contains divalent metal transporter as well as mobilferrin. *Am J Physiol Gastrointest Liver Physiol.* 2002;282(3):G534-539.

58.	Simovich M, Hainsworth LN, Fields PA, Umbreit JN, Conrad ME. Localization of the iron transport proteins Mobilferrin and DMT-1 in the duodenum: the surprising role of mucin. *Am J Hematol.* 2003;74(1):32-45.

59.	Sharp P, Srai SK. Molecular mechanisms involved in intestinal iron absorption. *World J Gastroenterol.* 2007;13(35):4716-4724.

60.	Pinto JP, Ramos P, de Almeida SF, et al. Protective role of calreticulin in HFE hemochromatosis. *Free Radic Biol Med.* 2008;44(1):99-108.

61.	Oliveira SJ, de Sousa M, Pinto JP. ER Stress and Iron Homeostasis: A New Frontier for the UPR. *Biochem Res Int.* 2011;2011:896474.
